# Synthesis of spiroindolenines through a one-pot multistep process mediated by visible light

**DOI:** 10.3762/bjoc.20.230

**Published:** 2024-10-29

**Authors:** Francesco Gambuti, Jacopo Pizzorno, Chiara Lambruschini, Renata Riva, Lisa Moni

**Affiliations:** 1 Department of Chemistry and Industrial Chemistry, University of Genoa, Via Dodecaneso 31, 1646 Genova, Italy

**Keywords:** isocyanide, multicomponent reactions, one-pot reaction, oxidation, spiroindolenine, Ugi reaction, visible light

## Abstract

Spiro-heterocyclic indolenines are privileged scaffolds widely present in numerous indole alkaloids. Here, we develop a novel approach for the one-pot multistep synthesis of different spiro[indole-isoquinolines]. The protocol proposed involves the visible light mediated oxidation of *N*-aryl tertiary amines using bromochloroform with the generation of a reactive iminium species, which reacts with an isocyanide and an electron-rich aniline in a three-component Ugi-type reaction to give an α-aminoamidine. This compound might undergo an additional visible light-mediated oxidation to furnish a second iminium intermediate, which acts as electrophile in an intramolecular electrophilic aromatic substitution giving the final spiro-indolenine. The scope of the process has been investigated with respect to all three components. Simple operations, mild conditions, and good yields make this strategy a convenient and sustainable way to obtain novel spiro-indolenine derivatives.

## Introduction

**Figure 1:**
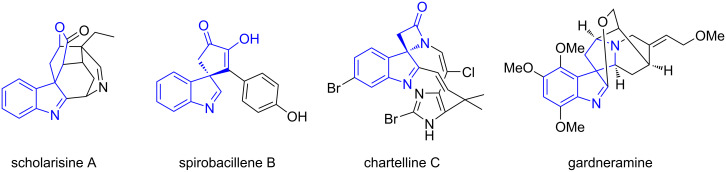
Selected natural products containing spiro-indolenines.

Diversity-oriented synthesis (DOS) is a successful approach to biologically active scaffolds directed to create an enormous exploratory space in pharmaceutical hit discovery [1–2]. Among the common compound collections for drug discovery, the introduction of spirocyclic elements presents an attractive strategy for increasing molecular complexity without increasing molecular weight, and at the same time, for introducing structural novelty for patentability [3]. However, the construction of spirocyclic motifs is often associated with increased synthetic effort and number of synthetic steps. Hence, developing efficient strategies to access spirocyclic targets with structural diversity is highly desirable and valuable. Among the bioactive compounds containing spiro atoms, the spiro-indolenines, specially the spiro-heterocyclic indolenines, can be considered as a privileged scaffold, present in several natural products with interesting biological activities, as depicted in Figure 1 [4–7].

**Scheme 1:**
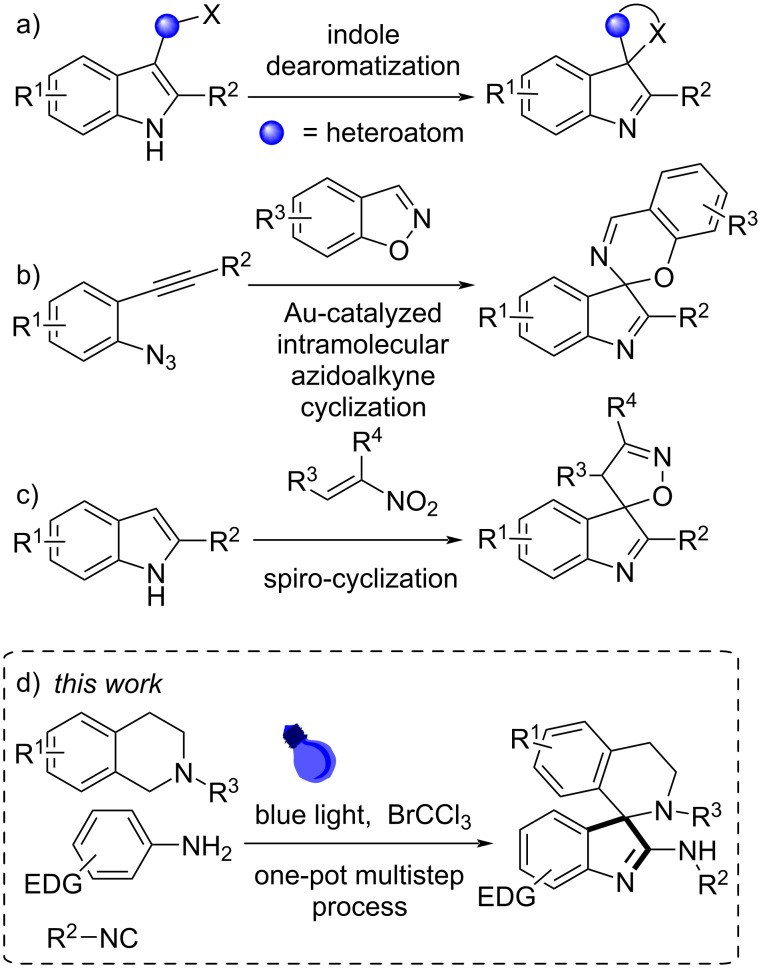
Synthesis of spiro[indole-heterocycles].

Among the known methods of synthesizing spiro-heterocyclic indolenines, the dearomative cyclization of the indole derivatives is the most popular (Scheme 1a). For instance, the efficient synthesis of spiro[indoline-3,2′-pyrrolidines] [8–10] or spiro-isoxazoles [11] through different dearomatization processes has been reported. Recently, Ramana and Dothe have proposed an elegant gold-catalyzed protocol to obtain spiro[benzo[*e*][1,3]oxazine-2,3’-indoles] starting from 2-alkynylphenyl azides and 1,2-benzisoxazoles [12] (Scheme 1b). However, all these processes generally involve the preparation of starting materials, often not trivial. On the other hand, in 2018 Aksenov and Rubin reported the acid-catalyzed [4 + 1] cycloaddition of commercially available substrates, as indoles and nitroalkenes to give spiro-isoxazoles (Scheme 1c), but acceptable yields were obtained just using nitrostyrenes [13–14].

Despite these examples, the highly efficient construction of structurally diverse spiro-heterocyclic indolenines from readily available starting materials under mild conditions and in a diversity-oriented fashion remains desirable.

In this contest, oxidative isocyanide-based multicomponent reactions (oxidative IMCRs) can be considered as a convenient tool [15]. Actually, the use of an external oxidant in combination with a multicomponent process allows to improve the scope of known MCRs by broadening the diversity and availability of starting materials, or by transforming unstable multicomponent adducts into stable compounds.

Following our research interest in developing new efficient methods for the synthesis of heterocycles by multicomponent processes and domino reactions [16–20], here we disclosed the oxidative one-pot four-step synthesis of 2-amino-3,3’-spiroindolenines using readily available tertiary amines, electron-rich anilines and isocyanides as starting materials (Scheme 1d). The synthetic procedure is achieved using blue light irradiation and bromotrichloromethane (BrCCl_3_), as a one-pot procedure, minimizing chemical wastes, avoiding purification of intermediates, and simplifying practical aspects.

## Results and Discussion

**Scheme 2:**
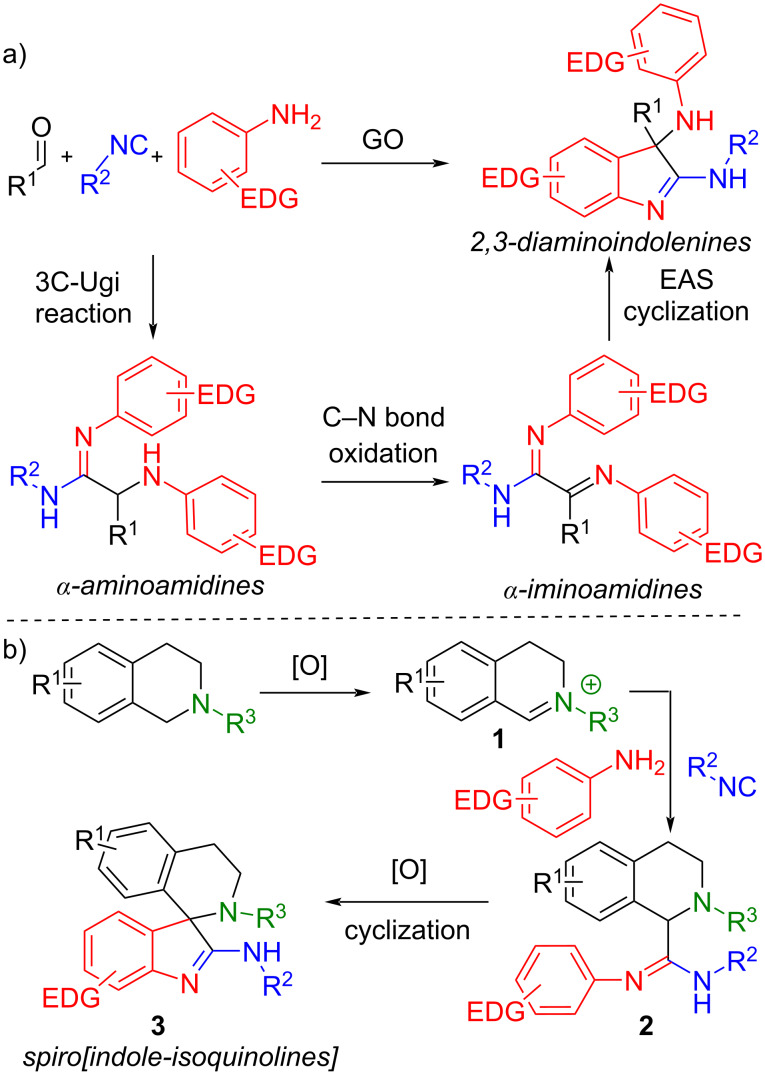
Synthetic strategy for the new synthesis of 2,3-diaminoindolenines [21] and spiro[indole-isoquinolines].

We have recently published the one-pot multistep synthesis of unprecedent 2,3-diaminoindolenines using graphene oxide (GO) as heterogeneous catalyst [21]. The protocol involves the three-component Ugi (3C-Ugi) reaction between aldehydes, isocyanides and 2 equivalents of electron-rich anilines to give α-aminoamidines, which undergo a C–N bond oxidation to generate α-iminoamidines and then a final cyclization takes place (Scheme 2a). Although the synthetic procedure allowed the preparation of highly substituted complex structures under mild reaction conditions and on gram scale, it suffers from a limited structural diversity. Since two anilines are incorporated in the final product, we speculated that the diversification of them could lead to different products.

Anyway, first attempts to use two different anilines in the reaction failed, giving complex mixtures of α-aminoamidines even using sequential or slow addition of reactants. Therefore, based on the previous studies on the oxidative 3-component Ugi–Joullié reaction [22–24] and our precedent experience [25–27], we speculated that cyclic imines could be good substrates for our reaction, thanks to their higher rigidity and stability.

As depicted in Scheme 2b, our plan provides: the oxidation of *N*-aryltetrahydroisoquinolines (*N*-Ar-THIQs) generating in situ the iminium ions **1**, the 3C Ugi-type reaction of an electron-rich aniline, an isocyanide and **1**; the subsequent oxidation of the Ugi-type product **2**; and the final cyclization to give the spiro[indole-THIQs] **3**.

Based on our experience on the use of graphene oxide (GO) as heterogeneous catalyst to promote MCRs and subsequent C–N bond oxidation [16,21], we first investigated the GO-promoted oxidation of *N*-Ph-THIQ and the subsequent 3C Ugi reaction to give α-aminoamidine **2a**. Applying the previously optimized reaction conditions, we observed good conversion of the starting material, but the desired compound was isolated in very poor yield, as the α-aminoamide **4a** resulted the main product (Table 1, entry 1). We were surprised about that, as, even if water is present as co-solvent in the reaction mixture, we have never noted the formation of the α-aminoamide in our previous work. Repeating the reaction in the absence of water suppressed the **4a** formation but with a concomitant drop of the conversion, resulting again in a poor yield of **2a** (Table 1, entry 2).

**Table 1:** Initial investigation of the GO-promoted oxidation/3C-Ugi reaction process.

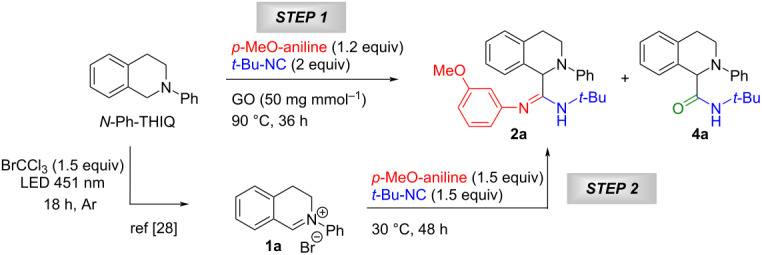				

Entry	Step 1	Step 2	Yield **2a**^a^	Yield **4a**^a^

1	CH_3_CN/H_2_O (4:1)	–	12%	26%
2^b^	CH_3_CN^b^	–	22%	–
3^b^	–	GO (30 mg mmol^-1^) MeOH	64%	–
4^b^	–	MeOH	54%	–
5^b^	–	CF_3_CH_2_OH	43%	–
6^b^	–	CH_3_CN	63%	–

^a^Isolated yield after column chromatography; ^b^reactions were carried out under argon atmosphere with dry solvents.

Thus, we demonstrated that GO was able to oxidize the substrate, which reacted with the 3-methoxyaniline and *tert*-butyl isocyanide affording the α*-*aminoamidine, albeit inefficiently. Besides, the role of GO in the 3C Ugi reaction remained unclear. Actually, the iminium ion formed after the oxidation is already quite activated to react with isocyanide without the presence of a catalyst. In order to establish the role of GO we carried out the 3C Ugi-type reaction starting from iminium ion **1a**, freshly prepared by visible light irradiation in the presence of bromochloroform [28]. This protocol resulted quite convenient as can be conducted under argon atmosphere to prevent the interaction of oxygen or reactive oxygen intermediates, which are not always compatible with the substrates, as well as the key intermediates. Moreover, BrCCl_3_ and its reduced form (chloroform) can be easily removed from the reaction mixture thanks to their low boiling point, allowing the easy purification of the products.

As expected, the reaction conducted in the absence of the carbocatalyst led to the obtainment of **2a** with comparable yield (Table 1, entries 3 and 4), demonstrating that **1a** is a sufficiently activated substrate for the 3C Ugi-type reaction to occur. Interestingly, different solvents, such as trifluoroethanol or acetonitrile (Table 1, entries 5 and 6), had a low impact, affording **2a** in acceptable yield.

The possibility of conducting the MCR in acetonitrile, the same solvent in which the oxidation of the C–N bond is performed, pushed us to perform the synthetic process in a one-pot manner, adding all the components from the very beginning. So, the blue-light-promoted reaction between *N*-Ph-THIQ, 3-methoxyaniline and *tert*-butyl isocyanide in the presence of BrCCl_3_ was extensively optimized by varying several parameters, such as solvent, relative quantity of components and temperature (Table 2). We firstly irradiated with blue LEDs (451 nm) the mixture of the three components in the presence of 1.5 equiv of BrCCl_3_ for 18 h at room temperature under argon atmosphere (Table 2, entry 1).

**Table 2:** Investigation of the one-pot four-step synthesis of spiro[indole-THIQ] **3a**.

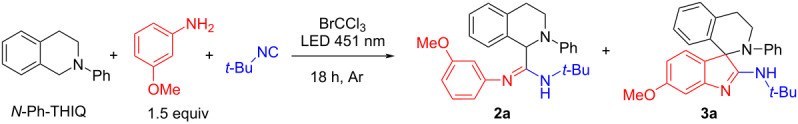						

Entry	*t*-Bu-NC	BrCCl_3_	Solvent (0.2 M)	Temp.	Yield **3a**^a^	Yield **2a**^a^

1	1.5 equiv	1.5 equiv	CH_3_CN	20 °C	43%	3%
2	2.2 equiv	2.2 equiv	CH_3_CN	20 °C	51%	–%
3	2.2 equiv	3 equiv	CH_3_CN	20 °C	56%	2%
4	2.2 equiv	3 equiv	EtOH	20 °C	40%	18%
5	2.2 equiv	3 equiv	EtOAc	20 °C	2%	13%
**6** **^b^**	**2.2 equiv**	**3 equiv**	**CH** **_3_** **CN**	**30 °C**	**61%**	**3%**
7	2.2 equiv	3 equiv	EtOH	30 °C	53%	5%

^a^Isolated yield after column chromatography with alumina; ^b^optimized reaction conditions: *N*-Ph-THIQ (1 equiv), 3-methoxyaniline (1.5 equiv), BrCCl_3_ (3 equiv) in CH_3_CN (0.2 M), *tert*-butyl isocyanide (2.2 equiv), argon atmosphere, 451 nm (blue LEDs), 30 °C, 24 h.

To our surprise, the reaction provided only traces of the expected α-aminoamidines **2a**, while the spiro-compound **3a** was the main product. **3a** was the result of a four-step one-pot process where an oxidation of *N*-Ph-THIQ, a 3C Ugi-type reaction, a second oxidation of the Ugi-type product **2a** and a final cyclization took place, as we had initially planned. Excited by this result, we optimized the reaction conditions to make the process more efficient.

Increasing the amount of isocyanide (Table 2, entry 2) and BrCCl_3_ (Table 2, entry 3) influenced the yield, obtaining **3a** up to a yield of 56% using a double quantity of polyhalomethane. Different solvents were detrimental (Table 2, entries 4, 5 and 7), while higher temperature improved the efficiency of the process (Table 2, entry 6). Under the optimized conditions we were able to obtain **3a** with a 61% isolated yield, which can be considered an excellent result, as it is related to a four-step process (an average yield of 88% each step).

Since, during the optimization of the reaction conditions, we have noticed a certain instability of the spiro-compounds through chromatography on silica gel, we have initially carried out the purifications using alumina as the stationary phase. However, during the scope studies, alumina proved to be ineffective as chromatographic stationary phase, providing the final products with insufficient purity, or not separating efficiently the side products. For this reason, we implemented the synthetic process treating the reaction mixture with 3 equivalents of Et_3_N before evaporating the solvent and using Et_3_N as additive for the eluent of the chromatography on silica gel. With these improvements, we have demonstrated that the yields are comparable to those obtained with purification on alumina but reaching significantly higher degrees of purity of the final products (see Supporting Information File 1).

**Scheme 3:**
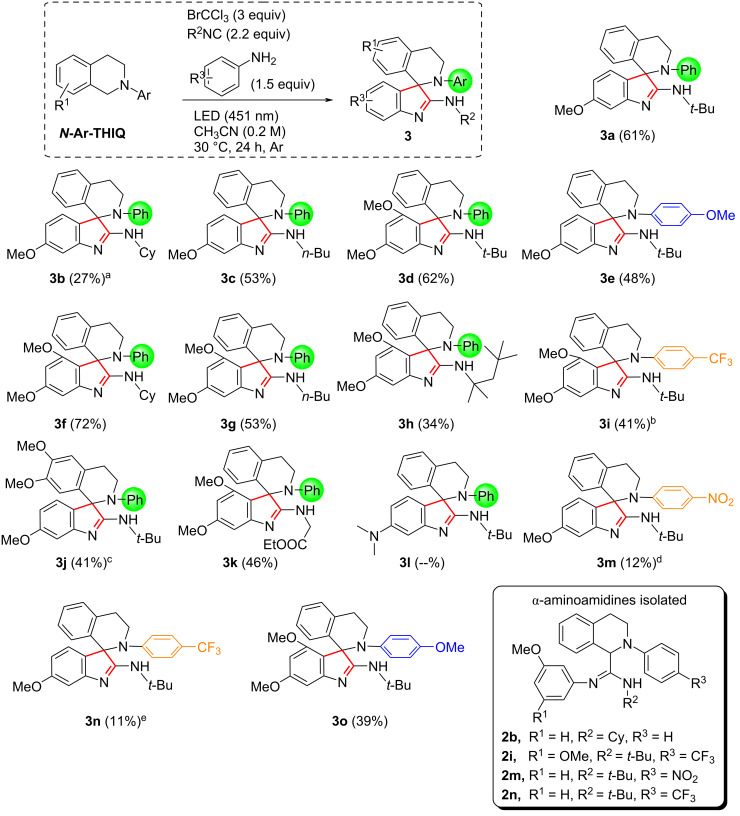
Scope of the synthesis of spiro[indole-THIQs]. ^a^α-aminoamidine **2b** has been isolated (54%) too; ^b^α-aminoamidine **2i** has been isolated (16%) too; ^c^white LEDs were used; ^d^α-aminoamidine **2m** has been isolated (28%) too; ^e^α-aminoamidine **2n** has been isolated (20%) too.

Having the optimized conditions in our hands, we moved on to establish the scope (Scheme 3).

Different *N*-arylated-THIQs, isocyanides and electron-rich anilines have been combined. The overall yields of the protocol are good considering its multistep behavior.

Very good results were obtained when performing the reaction with a combination of *N*-Ph-THIQ and 3-methoxyaniline (**3a** and **3c**) or 3,5-dimethoxyanaline (**3d**, **3f** and **3g**). When a THIQ with electron-rich aryl group was employed, the final spiroindolenines **3e** and **3o** were isolated in good yield.

On the other hand, when *p*-CF_3_-phenyl-THIQ or *p*-NO_2_-phenyl-THIQ were reacted, the conversion was not complete and the spiroindolenines (**3i**, **3m** and **3n**) were obtained in poor yield together with the corresponding α-aminoamidines **2i**, **2m** and **2n** (16%, 28% and 20%, respectively). Different isocyanides, such as linear, branched, or functionalized (**3k**) can be used. Surprisingly, the use of cyclohexyl isocyanide does not lead to the complete conversion of the α-aminoamidine **2b** into the corresponding spiro compound **3b**, which were isolated in 54% and 27% yields, respectively. Employing electron-rich *N*-Ar-THIQ, no reaction occurred using optimized conditions. Based on the literature [28], the formation of an electron donor–acceptor complex between bromochloroform and *N*-Ar-THIQ seems essential for the reaction to occur. Since we did not observe the formation of any color during the irradiation of the solution of BrCCl_3_ and this specific THIQ, we postulated that blue light was not the optimal one. Since, the absorption spectrum of the mixture showed better absorption at higher wavelengths (see Supporting Information File 1), we repeated the synthesis by irradiating with white LEDs, succeeding in isolating product **3j** in acceptable yield.

**Scheme 4:**
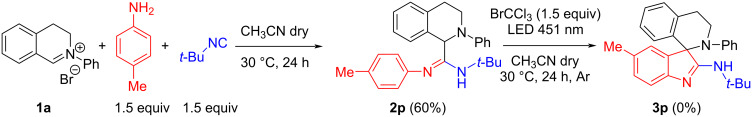
Two-step synthesis using *p*-methylaniline.

However, the synthetic protocol presents some limitations: employing non-electron-rich anilines (such as aniline or *p*-methylaniline) the reaction was unsuccessful, and the desired spiro-compounds were not obtained. To deeply investigate this point, we carried out the synthetic procedure with *p*-methylaniline step by step (Scheme 4).

While the 3C Ugi-type reaction between iminium ion **1a**, *p*-methylaniline and *tert*-butyl isocyanide gave product **2p** in good yield, when the α-aminoamidine was subjected to the oxidation–cyclization step, the starting material was completely converted, but **3p** was not isolated. Thus, we supposed that the second visible light-mediated oxidation properly occurred generating the iminium ion which underwent decomposition. Even using 3-(*N*,*N*-dimethylamino)aniline, the corresponding spiro compound **3l** was not obtained. In this case, it is assumed that the highly acidic environment of the reaction protonates the amino group, making the final cyclization less efficient.

Finally, we have tried to extend the synthetic protocol to other benzo-fused *N*-heterocycles. We investigated the benzo-fused 7-membered nitrogen heterocycles, both for our previous experience in applying them in MCRs [25,27] and for their known interest in medicinal chemistry.

**Scheme 5:**
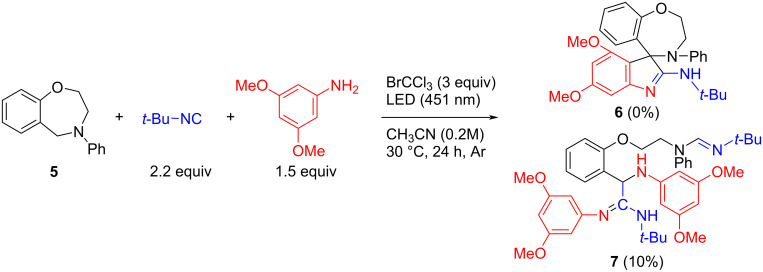
Investigation of the one-pot four-step synthetic protocol employing *N*-Ph-benzoxazepine **5**.

Towards this goal, *N*-phenylbenzoxazepine **5** was synthesized and subjected to the one-pot protocol within *tert*-butyl isocyanide and 3,5-dimethoxyaniline (Scheme 5). Unfortunately, the reaction mixture appeared very complex and the desired product **6** was not observed. Instead, among the several side-products formed, we were able to isolate α-aminoamidine **7**, as the result of a 3C Ugi-type reaction between an aldehyde obtained from an oxidation–hydrolysis process of compound **5**, *tert*-butyl isocyanide and 2 equivalents of aniline, and the simultaneous insertion of an isocyanide into the N–H bond. This evidence shows that **5** was able to be oxidized but the iminium ion formed was not stable enough to take part in the subsequent reaction, preferring to hydrolytically open.

In order to test the synthetic utility of this procedure we also carried out a gram scale reaction. First, the suitability of the one-pot protocol toward the synthesis of spiro-derivative **3d** was verified by applying optimized conditions (Table 3, entry 1). To do this a different photoreactor has been used (see Supporting Information File 1 for details) [29].

**Table 3:** Gram scale reaction for the synthesis of spiro[indole-THIQs] **3**.

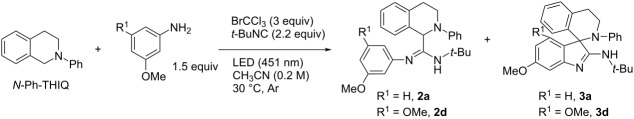					

Entry	*N*-Ph THIQ (g)	R^1^	Time (h)	Yield **3**^a^	Yield **2**^a^

1	1.00	OCH_3_	24	48% (**3d**)	28% (**2d**)
2	0.50	OCH_3_	48	68% (**3d**)	3% (**2d**)
3	1.00	H	48	51% (**3a**)	19% (**2a**)

^a^Isolated yield after column chromatography.

While the overall yield resulted even better (76% starting from 1 g of *N*-Ph-THIQ), a slight decrease of the conversion of α-aminoamidine **2d** into the spiro[indole-THIQ] **3d** was observed. As expected, the use of a larger-diameter reactor caused a low penetration of the irradiation, thus leading to lower efficiency.

Repeating the reaction on 500 mg for a longer reaction time a notable 68% isolated yield of **3d** was obtained (Table 3, entry 2). These slightly different conditions were applied to the gram scale synthesis of **3a** obtaining good results (Table 3, entry 3).

**Figure 2:**
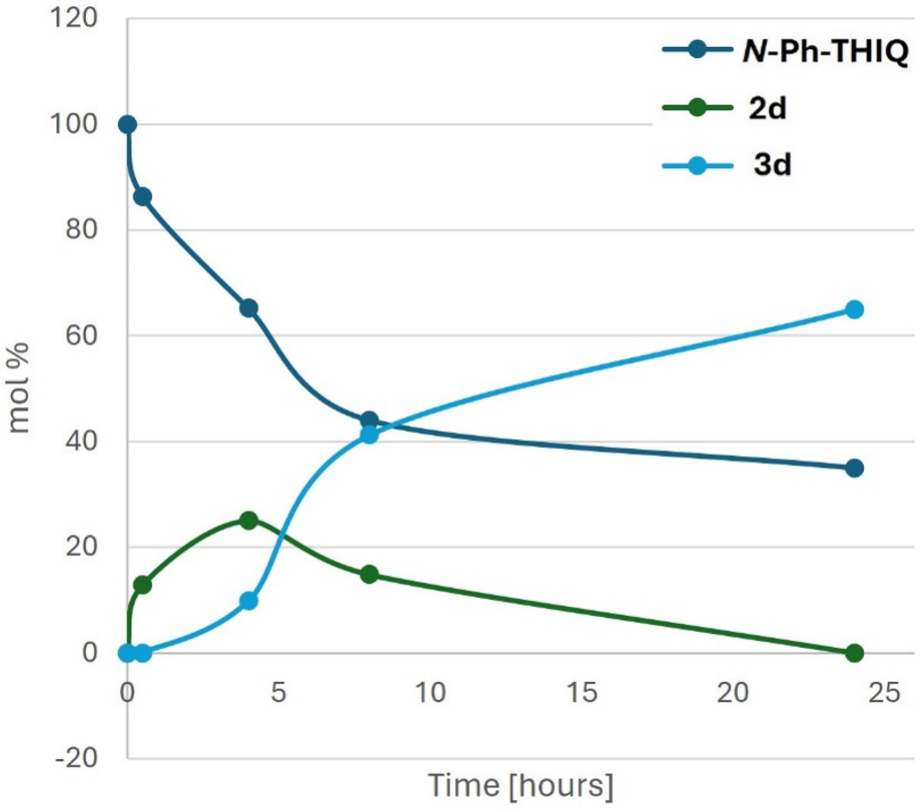
Time profile of the reaction of *N*-Ph-THIQ, 3,5-dimethoxyaniline and *t*-BuNC conducted under optimized conditions.

To obtain some information of the reaction mechanism and the intermediates involved, the relationship between the product conversions and the reaction time was investigated in the reaction between *N*-Ph-THIQ, 3,5-dimethoxyaniline and *t*-BuNC in the presence of BrCCl_3_ under light irradiation (Figure 2). This time profile shows that, as soon as the *N*-Ph-THIQ is transformed into iminium ion **1a**, this compound instantly takes part in the Ugi-type reaction with 3,5-dimethoxyaniline and *t*-BuNC giving the α-aminoamidine **2d**. After 4 h, **2d** starts to be converted slowly into the final product **3d**, thanks to a second oxidation and a final cyclization. This study indicates that the second oxidation is faster than the multicomponent reaction, as intermediate **2d** is not accumulated in the reaction mixture, but easily undergoes the C–N bond oxidation and the subsequent cyclization.

**Scheme 6:**
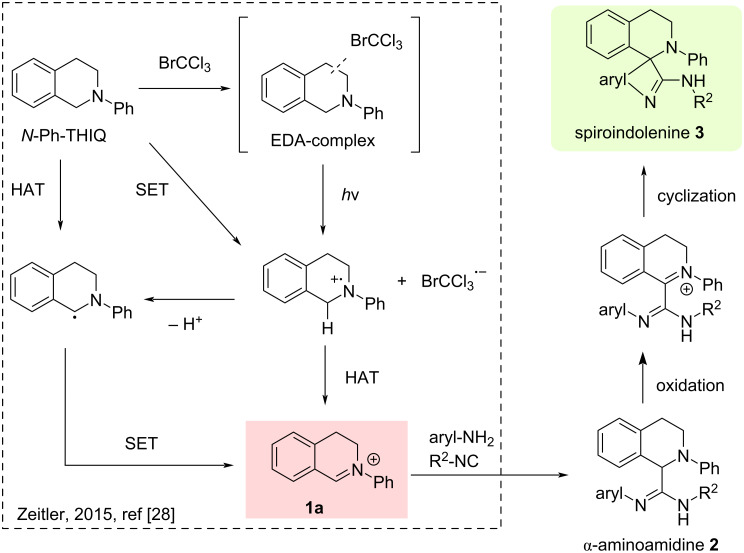
Proposed mechanism.

A probable mechanistic pathway for the formation of spiro-indolenine is outlined in Scheme 6. Based on the results reported by Zeitler [28], several mechanisms are involved in the oxidation of *N-*Ph-THIQ. The most probable involves the photoexcitation of the EDA (Electron Donor-Acceptor) complex promoting an electron transfer from *N*-Ph-THIQ to BrCCl_3_ to afford the amine radical cation and highly reactive BrCCl_3_ radical anion. Anyway, the *N*-Ph-THIQ can undergo numerous pathways towards the iminium ion **1a** (see reference [28] for details). The oxidation of compound **2d** may occur according to the same mechanism. However, alternative mechanisms, such as the direct hydride transfer from compound **2** to the iminium ion **1a**, cannot be ruled out (see Supporting Information File 1).

## Conclusion

In conclusion, we have developed a novel visible light-promoted synthesis of spiro[indole-isoquinolines] employing simple and readily available starting materials. Our one-pot four-step synthesis allows the preparation of complex structures without using catalysts, and purification of intermediates. Remarkably, the reaction can be performed even on gram scale without loss of efficiency. Studies directed toward evaluating the biological activities of the spiroindolenines are currently underway in our laboratory, and the results will be reported in due course.

## Experimental

### General methods

^1^H, ^13^C and ^19^F NMR spectra were recorded on a JEOL 400 spectrometer (at 400 MHz, 101 MHz and 376 MHz, respectively). Unless otherwise stated, NMR spectra were recorded using residual solvent as the internal standard; ^1^H NMR: TMS = 0.00; (CD_3_)_2_SO = 2.50; and ^13^C NMR: CDCl_3_ = 77.16; (CD_3_)_2_SO = 39.52. Data for ^1^H NMR spectra are reported as follows: chemical shift (δ ppm), multiplicity, coupling constants (Hz) and integration. Data for ^13^C NMR spectra are reported in terms of chemical shift (δ ppm). Interpretation of spectra has been made also with the aid of gCOSY, gHSQC and gHMBC experiments. The following abbreviations are used to indicate the multiplicity in NMR spectra: s, singlet; d, doublet; t, triplet; q, quartet; m, multiplet. For some compounds the extra peaks are due to residual of the starting material (<5%) due to difficulties in the purification. IR spectra were recorded as solid, oil, or foamy samples, with the ATR (attenuated total reflectance) method. TLC analyses were carried out on pre-coated Merck silica gel 60 F254 plates or Aluminum oxide on TLC-plates and viewed at UV (254 nm) and developed with Hanessian stain (dipping into a solution of (NH_4_)_4_MoO_4_·4H_2_O (21 g) and Ce(SO_4_)_2_·4H_2_O (1 g) in H_2_SO_4_ (31 mL) and H_2_O (469 mL) and warming). *R*_f_ were measured after an elution of 7–9 cm. Column chromatographies were done with the "flash" methodology using 220–400 mesh silica or 150 mesh aluminum oxide, activated, neutral, Brockmann I. Petroleum ether (40–60 °C) is abbreviated as PE. HPLC analysis was performed on Agilent HP 1100 equipped with a DAD detector (220 nm) and column ACE Excel 3 C18-AR (3 μm, 3 × 150 mm^2^). Mass analysis was performed on a Microsaic 4000 MiD^®^ mass spectrometer. HRMS analyses were performed by using the ionization method ESI+ with a 6230 TOF LC/MS, Agilent Technologies. Unless otherwise noted, analytical grade solvents and commercially available reactants were used without further purification. Common reagents were purchased from commercial sources and were used without further purification. Graphene oxide (GO) was purchased from Graphenea. All products were characterized by ^1^H,^13^C, ^19^F (when fluorine is present) NMR, IR and HRMS.

**General procedure for the one-pot synthesis of spiro[indole-isoquinoline] 3a:** A solution of *N*-phenyltetrahydroisoquinoline (1 equiv, 0.48 mmol, 100 mg), 3-methoxyaniline (1.5 equiv, 0.72 mmol, 81 µL) and BrCCl_3_ (3 equiv, 1.44 mmol, 140 µL) in CH_3_CN (2.4 mL, 0.2 M) in a vial was degassed with argon for 2 minutes, then *tert*-butyl isocyanide (2.2 equiv, 1.06 mmol, 120 μL) was added and the vial sealed. The mixture was irradiated at 30 °C at 451 nm (blue LEDs) under magnetic stirring for 24 h. The reaction mixture was treated with Et_3_N (3 equiv, 1.44 mmol, 200 µL), concentrated and the residue was purified by column chromatography on silica gel with PE/Et_2_O (from 85:15 to 75:25) to give **3a** (121 mg, 61%), as yellow oil; IR (ATR) ν̃ (cm^−1^): 3393, 3367, 3051, 2995, 2967, 2923, 2860, 2828, 1916, 1851, 1706, 1623, 1598, 1562, 1525, 1491, 1472, 1464, 1451, 1437, 1422, 1387, 1359, 1327, 1296, 1274, 1250, 1222, 1207, 1186, 1176, 1151, 1119, 1096, 1080, 1057, 1040, 1026, 1009, 972, 964, 951, 943, 929, 909, 898, 854, 818, 810, 805, 782, 761, 744, 716, 695, 663, 655, 640, 624, 607; ^1^H NMR (400 MHz, CDCl_3_) δ 7.19 (dd, *J* = 7.9, 1.6 Hz, 1H, H Ar), 7.14 (td, *J* = 7.3, 1.3 Hz, 1H, H Ar), 7.11–7.04 (m, 2H, 2H Ar), 7.03–6.96 (m, 2H, 2H Ar), 6.93–6.86 (m, 3H, 3H Ar), 6.81 (d, *J* = 2.4 Hz, 1H, H Ar), 6.66 (dd, *J* = 8.0, 1.3 Hz, 1H, H Ar), 6.31 (dd, *J* = 8.1, 2.4 Hz, 1H, H Ar), 4.73 (s, 1H, NH), 3.98 (ddd, *J* = 11.9, 10.7, 3.5 Hz, 1H, 1H of CH_2_ THIQ), 3.76 (s, 3H, OCH_3_), 3.58 (ddd, *J* = 12.4, 5.7, 3.4 Hz, 1H, 1H of CH_2_ THIQ), 3.31 (ddd, *J* = 16.1, 10.7, 5.5 Hz, 1H, 1H of CH_2_ THIQ), 3.03 (dt, *J* = 15.9, 3.4 Hz, 1H, 1H of CH_2_ THIQ), 1.25 (s, 9H, 3CH_3_ of *t*-Bu); ^13^C NMR (101 MHz, CDCl_3_) δ 175.3 (Cq amidine), 160.9 (Cq near O), 158.0 (Cq near N), 149.7 (Cq near N), 136.8 (Cq Ar), 133.9 (Cq Ar), 132.7 (Cq Ar), 129.0 (CH Ar), 128.5 (2 CH Ar), 126.9 (CH Ar), 126.9 (CH Ar), 126.5 (CH Ar), 123.5 (CH Ar), 123.3 (CH Ar), 123.2 (2 CH Ar), 107.1 (CH Ar), 103.5 (CH Ar), 75.9 (Cq spiro), 55.4 (OCH_3_), 52.1 (Cq *t*-Bu), 47.9 (CH_2_), 30.9 (CH_2_), 28.5 (3 CH_3_ of *t*-Bu); HRMS (ESI^+^) (*m*/*z*) [M + H]^+^: calcd for C_27_H_30_N_3_O, 412.238; found, 412.2395.

## Supporting Information

File 1 Additional optimization studies; description of photochemical equipment; characterization data of compounds (^1^H, ^13^C and ^19^F NMR spectra, IR and UV–vis spectra).

## Data Availability

All data that supports the findings of this study is available in the published article and/or the supporting information to this article.
