# Supplementary material for: Synthesis of spiroindolenines through a one-pot multistep process mediated by visible light
Source: Beilstein J Org Chem. 2024 Oct 29;20:2722–31. doi: 10.3762/bjoc.20.230 (PMC11533113; doi:10.3762/bjoc.20.230)

BEILSTEIN JOURNAL OF ORGANIC CHEMISTRY

Supporting Information

for

Synthesis of spiroindolenines through a one-pot multistep process mediated by visible light

Francesco Gambuti, Jacopo Pizzorno, Chiara Lambruschini, Renata Riva and Lisa Moni

Beilstein J. Org. Chem. **2024,** *20,* 2722–2731. doi:10.3762/bjoc.20.230

Additional optimization studies; description of photochemical equipment; characterization data of compounds (^{1}H, ^{13}C and ^{19}F NMR spectra, IR and UV–vis spectra)

License and Terms: This is a supporting information file under the terms of the Creative Commons Attribution License (https://creativecommons.org/licenses/by/4.0). Please note that the reuse, redistribution and reproduction in particular requires that the author(s) and source are credited and that individual graphics may be subject to special legal provisions.
The license is subject to the *Beilstein Journal of Organic Chemistry* terms and conditions: (https://www.beilstein-journals.org/bjoc/terms)

Table of contents

General methods......S2

Photochemical equipment......S3

UV–vis spectrum of bromochloroform, THIQs and their mixture in CH_3CN......S5

Optimization of the synthesis of spiro[indole-THIQs]......S7

Possible mechanism for the formation of compound 7......S8

Control experiments......S9

Copies of IR spectra of compounds......S12

Experimental procedures and spectroscopic characterization......S18

Synthesis of spiro[indole-tetrahydroisoquinolines] 3......S22

References......S42

Copies of ^{1}H, ^{13}C and ^{19}F NMR spectra of compounds......S43

General methods

^{1}H, ^{13}C and ^{19}F NMR spectra were recorded on JEOL 400 (at 400 MHz, 101 MHz and 376 MHz respectively). Unless otherwise stated, NMR spectra were recorded using residual solvent as the internal standard ^{1}H NMR: TMS = 0.00; $(CD_3)_2SO$ = 2.50; and ^{13}C NMR: $CDCl_3$ = 77.16; $(CD_3)_2SO$ = 39.52. Data for ^{1}H NMR spectra are reported as follows: chemical shift (δ ppm), integration, multiplicity and coupling constants (Hz). Data for ^{13}C NMR spectra are reported in terms of chemical shift (δ ppm). Interpretation of spectra has been made also with the aid of gCOSY, gHSQC and gHMBC experiments. The following abbreviations are used to indicate the multiplicity in NMR spectra: s, singlet; d, doublet; t, triplet; q, quartet; m, multiplet. For some compounds the extra peaks are due to residual of the starting material (<5%) due to difficulties in the purification.
IR spectra were recorded as solid, oil, or foamy samples, with the ATR (attenuated total reflectance) method.
TLC analyses were carried out on pre-coated Merck silica gel 60 F254 plates or Aluminum oxide on TLC-plates and viewed at UV (254 nm) and developed with Hanessian stain (dipping into a solution of $(NH_4)_4MoO_4\cdot 4H_2O$ (21 g) and $Ce(SO_4)_2\cdot 4H_2O$ (1 g) in H_2SO_4 (31 mL) and H_2O (469 mL) and warming). R_f were measured after an elution of 7–9 cm.
Column chromatographies were done with the "flash" methodology using 220–400 mesh silica or 150 mesh aluminum oxide, activated, neutral, Brockmann I. Petroleum ether (40–60 °C) is abbreviated as PE.
HPLC analysis was performed on Agilent HP 1100 equipped with a DAD detector (220 nm) and column ACE Excel 3 C18-AR (3 µm, 3 × 150 mm^2). Mass analysis was performed on a Microsaic 4000 MiD® mass spectrometer. HRMS analyses were performed by using the ionization method ESI+ with a 6230 TOF LC/MS from Agilent Technologies.
Unless otherwise noted, analytical grade solvents and commercially available reactants were used without further purification. Common reagents were purchased from commercial sources and were used without further purification. Graphene oxide (GO) was purchased from Graphenea. For data sheet see: https://www.graphenea.com/collections/graphene-oxide/products/graphene-oxide-powder.
All products were characterized by ^{1}H,^{13}C, ^{19}F (when fluorine is present) NMR, IR and HRMS.

Photochemical equipment

One-pot syntheses of 2-amino 3,3'-spiroindolenines were conducted in a dedicated apparatus consisting of an aluminum cooling plate (LWH 160 × 100 × 25 mm) with 6 OSRAM® Oslon SSL 80 LDCQ7P (nominal 450 nm, royal blue) LEDs mounted in series powered by a MeanWell® LPC-20-700 constant current power supply (700 mA) and an aluminum vessel holder. The vessel holder (LWH 170 × 110 × 38 mm) holds the sealed vials 10 mm over the LEDs in a fixed position and allows the temperature regulation (the temperature is kept at 30 °C). Both the LED plate and the vessel were custom built, while the vials were purchased from Wicom International Co. (WIC43005, 5 mL crimp top vial 38.5 × 22.0 mm; WIC44510 20 mm crimp caps with 3.0 mm PTFE septum).

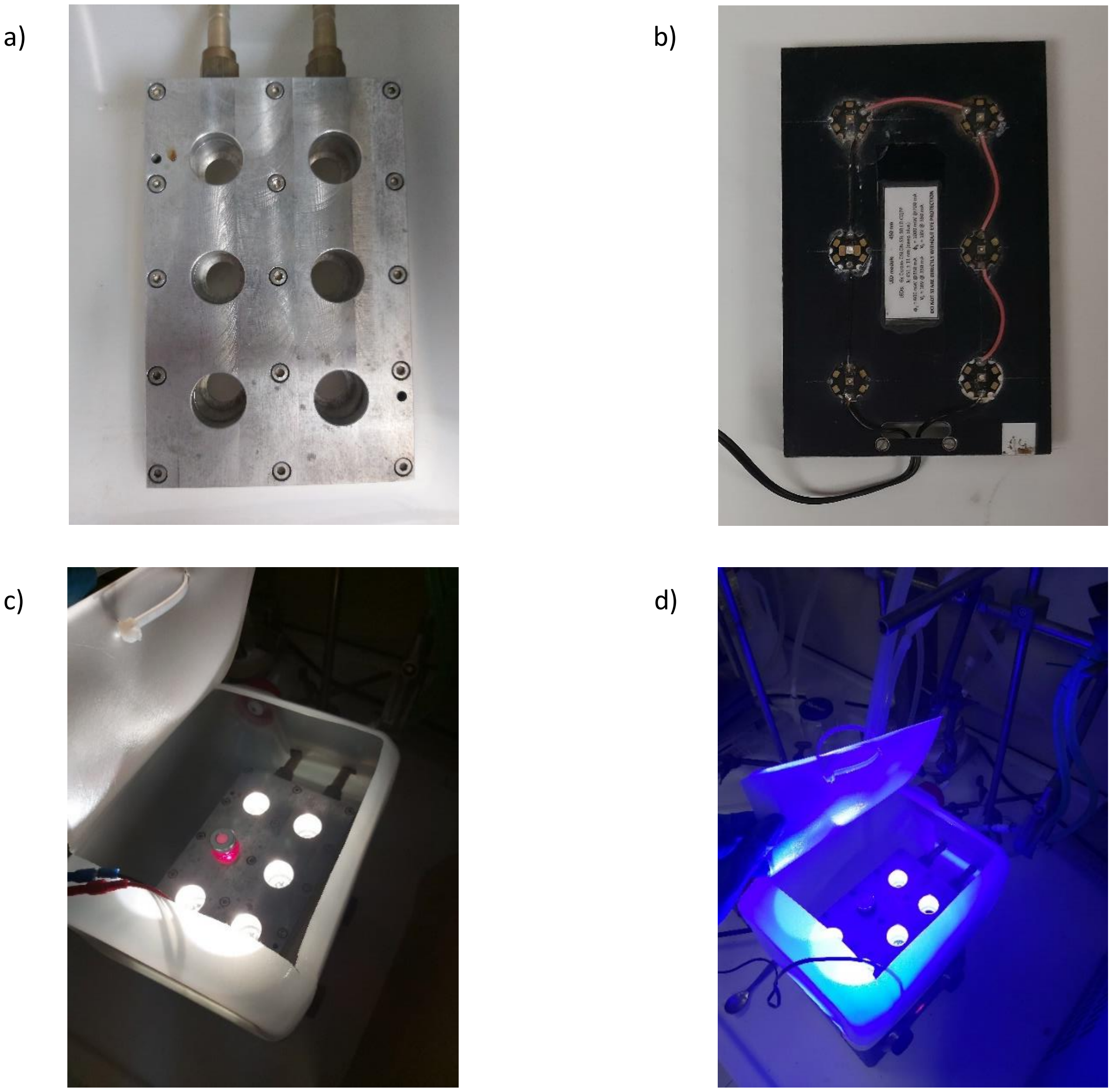

Figure S1 Setup for the photoreaction: a) aluminum cooling plate (LWH 160 × 100 × 25 mm); b) LED plate; c) photoreactor equipped with white LEDs; d) photoreactor equipped with blue LEDs.

Gram scale reactions were conducted in a custom-built photoreactor (Figure S2): a glass tube (internal diameter 23 mm) is placed in the middle of the irradiation system, which consists of 4 aluminum LEDs plates connected in series and mounted on a 200 × 200 mm wooden support. Each aluminum cooling plate (HWD 130 × 40 × 25 mm) bears 3 blue LEDs (40 mm apart) and is placed in the middle of each side of the square wooden support. To ensure proper heat dispersion of the LEDs plates, an 80 mm fan (powered independently with a 12V DC power supply) is placed in the middle of the wooden support in a hollow cavity to grant air circulation.

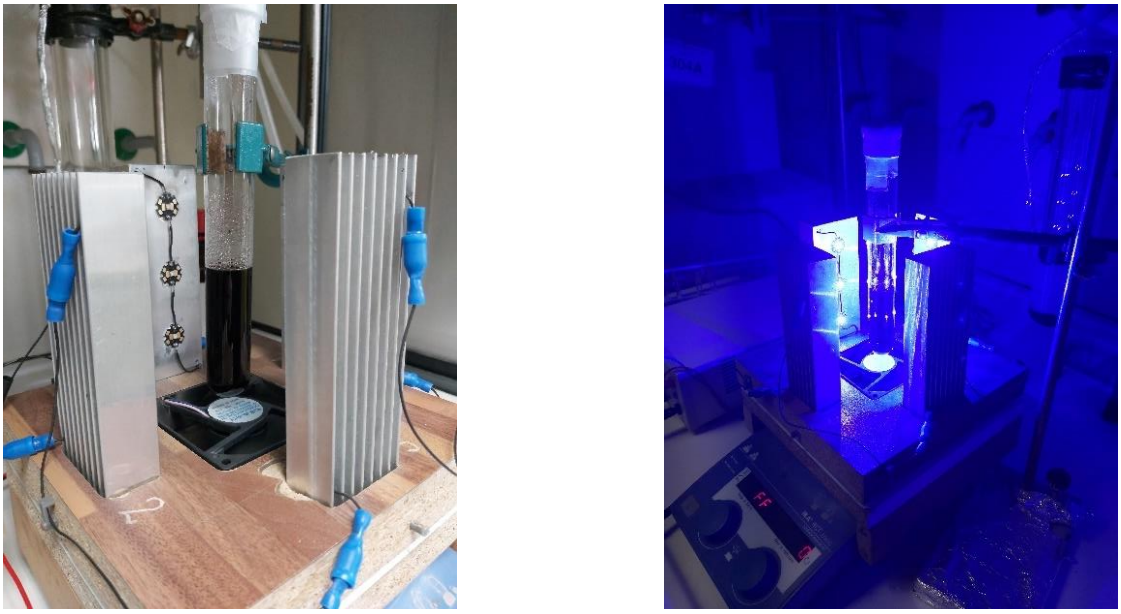

Figure S2 Photoreactor for gram scale reactions before (left) and during (right) the reaction.

UV–vis spectrum of bromochloroform, THIQs and their mixture in CH_3CN

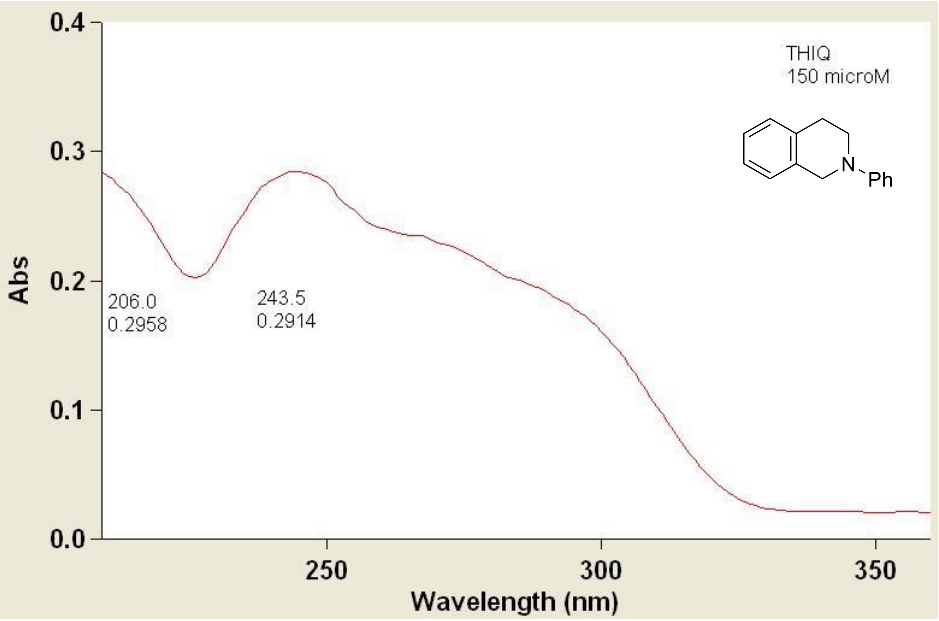

Figure S3 UV–vis spectra of *N*-Ph-THIQ in acetonitrile.

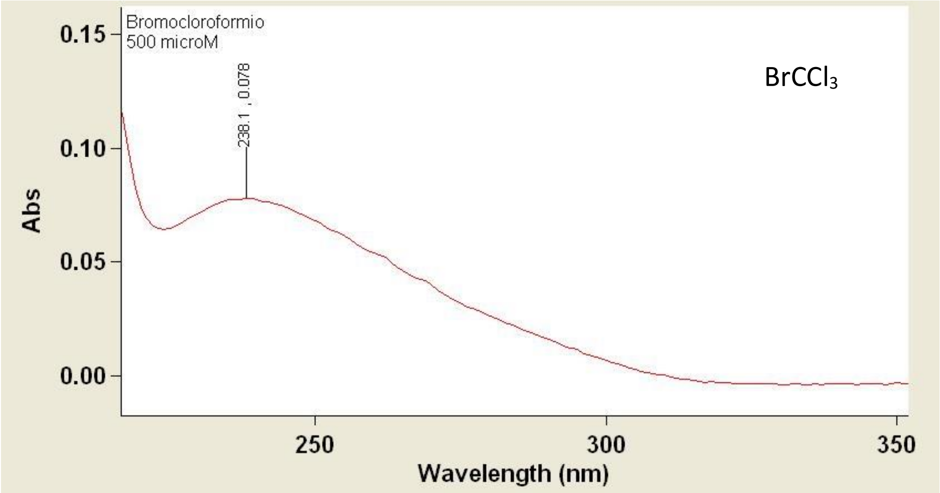

Figure S4 UV–vis spectra of $BrCCl_3$ in acetonitrile.

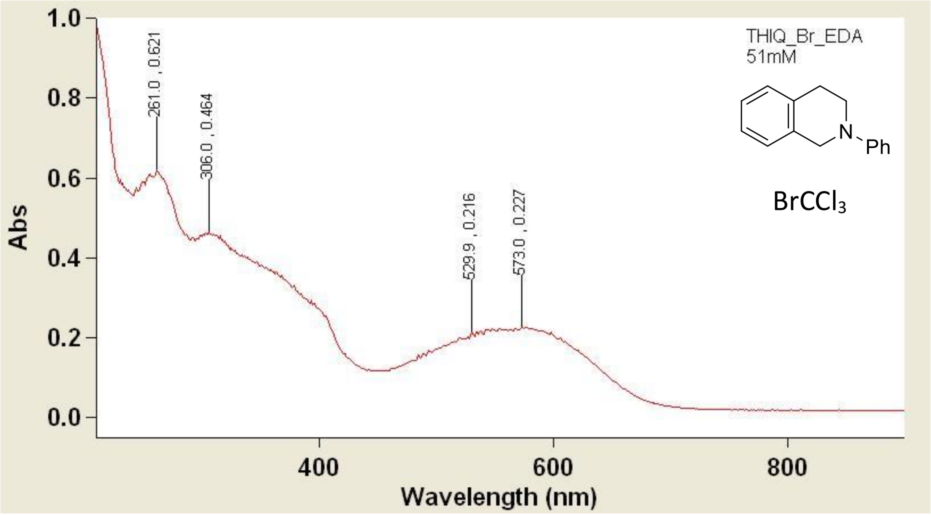

Figure S5 UV–vis spectra of *N*-Ph-THIQ and $BrCCl_3$ in acetonitrile.

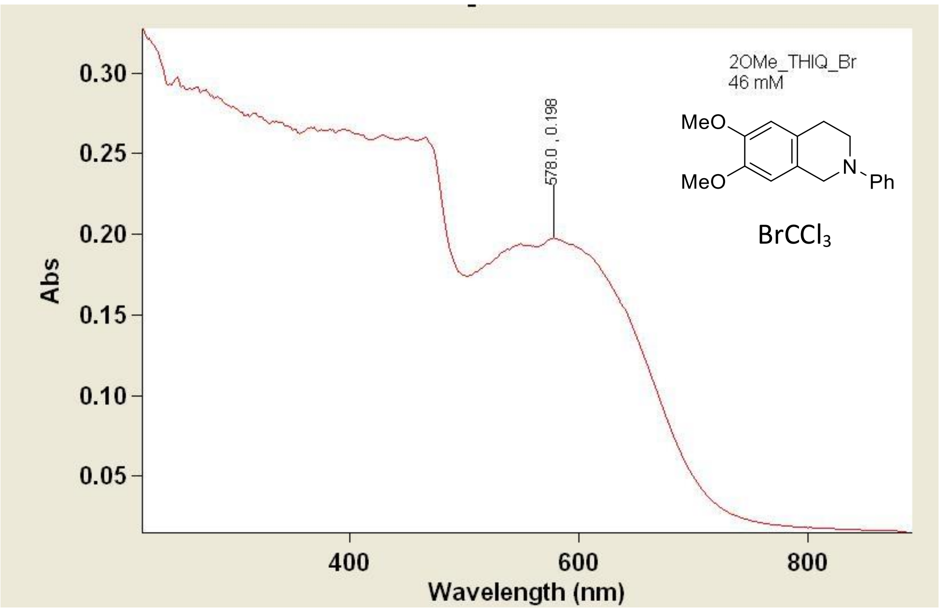

Figure S6 UV–vis spectra of **S4** and $BrCCl_3$ in acetonitrile.

Optimization of the synthesis of spiro[indole-THIQs]

Table S1: Optimization of the work up and purification of spiro[indole-THIQs] **3d**.

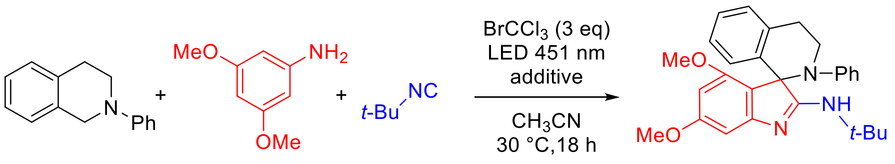

Entry	Additive	Work up	Stationary phase for chromatography	Yield **3d**[a]
1		--	Silica gel	53%
2		--	Silica gel; eluent with 1% Et_3N	60%
3		--	Alumine oxide; eluent with 1% Et_3N	68%[b]
4		Et_3N (3 equiv)	Silica gel; eluent with 1% Et_3N	62%
5	Et_3N (3 equiv)	Et_3N (3 equiv)	Silica gel; eluent with 1% Et_3N	51%
6	$CaCO_3$(1.5 equiv)	Et_3N (3 equiv)	Silica gel; eluent with 1% Et_3N	62%

[a] Isolated yield after column chromatography; [b] not pure by NMR analysis

Possible mechanism for the formation of compound 7

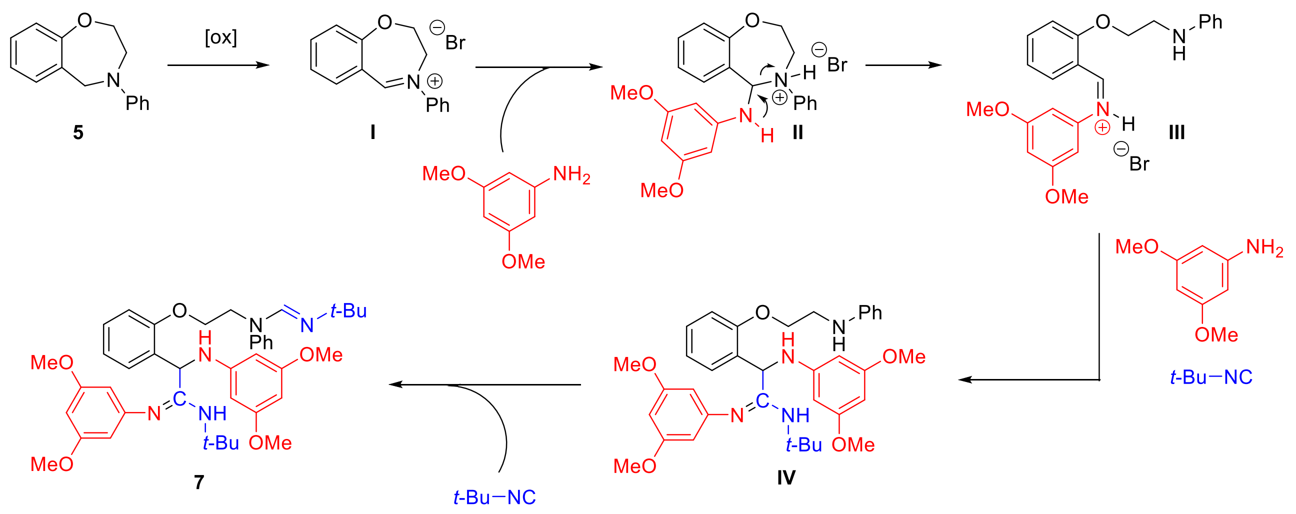

Scheme S1: the treatment of benzoxazepine **5** with $BrCCl_3$ and blue-light produces the imium ion **I**, which resulted less stable than that obtained from *N*-Ph-THIQ (analyzed by NMR). Thus, the attach of the aniline promotes the opening of the cycle with the formation of intermediate **III**, bearing a secondary amine and a new iminium ion. The last one is involved in an Ugi-type reaction giving the α-amino amidine **IV**, while the secondary amine undergoes a N–H insertion of the isocyanide forming compound **7**. The mechanism of the N–H insertion and the sequence of the two last steps are not clear. Experiments are underway to elucidate these aspects.

Control experiments

Table S2: Control experiments of the multistep one-pot synthesis of spiro[indole-isoquinolines]

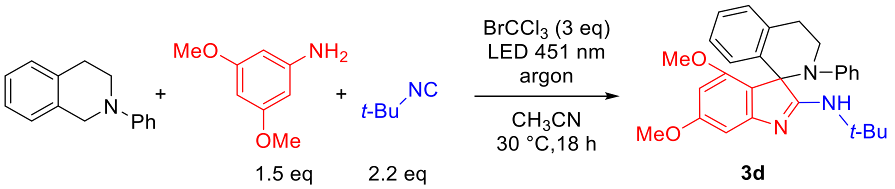

3d

Entry	Standard reaction conditions	Yield 3d[a]
1	3,5-diMeO-aniline (1.5 equiv), t-Bu-NC (2.2 equiv), $BrCCl_3$ (3 equiv), CH_3CN (0.2 M), LED (451 nm), 30 °C, 24 h, argon	62%
	**Variation from standard conditions**	
2	TEMPO (3 equiv)	nc[b]
3	No $BrCCl_3$, TEMPO (3 equiv)	--
4	No $BrCCl_3$, No light irradiation, TEMPO (3 equiv)	--
5	open air	47%

[a] Isolated yield after column chromatography; [b] product **3d** was not obtained pure.

To shed light on this multistep one-pot process, control experiments of the reaction were performed. Addition of TEMPO (2,2,6,6-tetramethylpiperidin-1-oxyl) to the reaction system did not prevent the formation of the desired product, even if caused a detrimental effect, as it has been isolated together with side-products and 1-hydroxy-2,2,6,6-tetramethylpiperidine, the reduced form of TEMPO (entry 2). We believed that TEMPO could be oxidized to the corresponding oxoammonium salt, and thus it mediated the oxidation of the substrate. However, the presence of $BrCCl_3$ is essential for the success of the process. When we used TEMPO as potential promoter in the absence of $BrCCl_3$ and light irradiation, the reaction did not proceed (see entries 3 and 4). The influence of oxygen was investigated performing the reaction open air (entry 5), obtaining a significant decrease in yield and a dirtier reaction crude.

Table S3: Control experiments of the conversion of α-aminoamidine **2d** into spiroindolenine **3d**

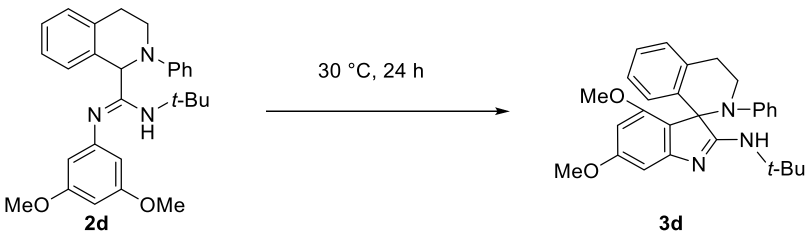

Entry	Solvent	atmosphere	Additive	Ratio 3d:2d[a]
1	CH_3CN	argon	$BrCCl_3$ (1.5 equiv) LED 451 nm	100:0
2	CH_3CN	argon	$BrCCl_3$ (1 equiv)	10:90
3	CH_3CN	argon	--	6:94
4	CH_3CN	air	--	8:92
5	THF	argon	--	7:93
6	CH_3CN	argon	**1a** (1equiv)	80:20[b]

[a] Calculated by NMR of the crude mixture; [b] **3d** was isolated in 75% yield through a column chromatography.

Control experiments were performed to investigate the role of $BrCCl_3$ and light irradiation on the conversion of α-aminoamidines **2** into spiro[indole-isoquinolines] **3**. When compound **2d** was irradiated at 451 nm in the presence of $BrCCl_3$, complete conversion was observed, confirming that α-aminoamidine is a good substrate for the photoinduced oxidation (entry 1). The formation of compound **3d** is almost completely suppressed in the dark (entry 2) or in the absence of $BrCCl_3$ (entry 3). Furthermore, neither the presence of oxygen (entry 4) nor acetonitrile (entry 5) affects the reactivity. To further investigate the influence of the presence of the reaction intermediates, we performed the reaction adding an equimolar amount of iminium ion **1a** (entry 6). Surprisingly we revealed a high conversion and product **3d** was also isolated in 75% yield. We currently are unsure of the mechanistic course of this

transformation. A literature precedent of related processes suggests a possible hydride transfer from **2d** to the iminium ion **1a**.[1] Anyway, a thorough study is required to elucidate the mechanism of this process.

Study of the reaction course through NMR spectroscopy

A solution of *N*-phenyltetrahydroisoquinoline (0.13 mmol, 27 mg), 3,5-dimethoxyaniline (0.20 mmol, 31 mg) and $BrCCl_3$ (0.39 mmol, 77 mg) in CH_3CN (0.7 mL) in a vial was degassed with argon for 2 minutes, then *tert*-butylisocyanide (0.29 mmol, 24 mg) was added and the vial sealed. The mixture was irradiated at 30 °C at 451 nm (blue LEDs) under magnetic stirring for different time (30 min, 4 h, 8 h and 24 h). The reaction mixture was treated with Et_3N (0.39 mmol), concentrated and the residue was analyzed by ^1H NMR spectroscopy. The mol % values were determined by integration of the following peaks: 4.41 ppm for *N*-Ph-THIQ, 5.30 ppm for **2d**, 5.88 ppm for **3d**.

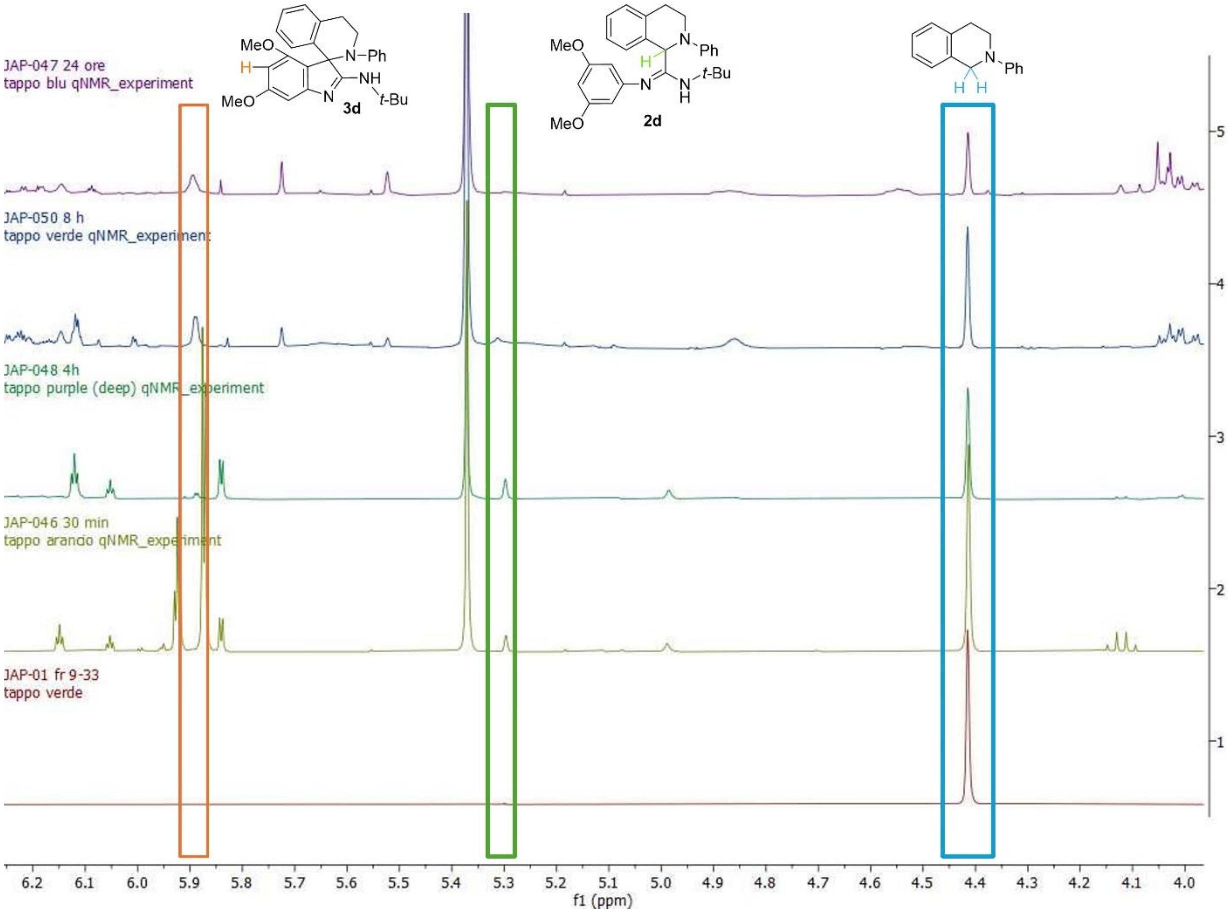

Figure S7: ^1H NMR spectra of the reaction mixture after 0 min, 30 min, 4 h, 8 h and 24 h.

Copies of IR Spectra of Compounds

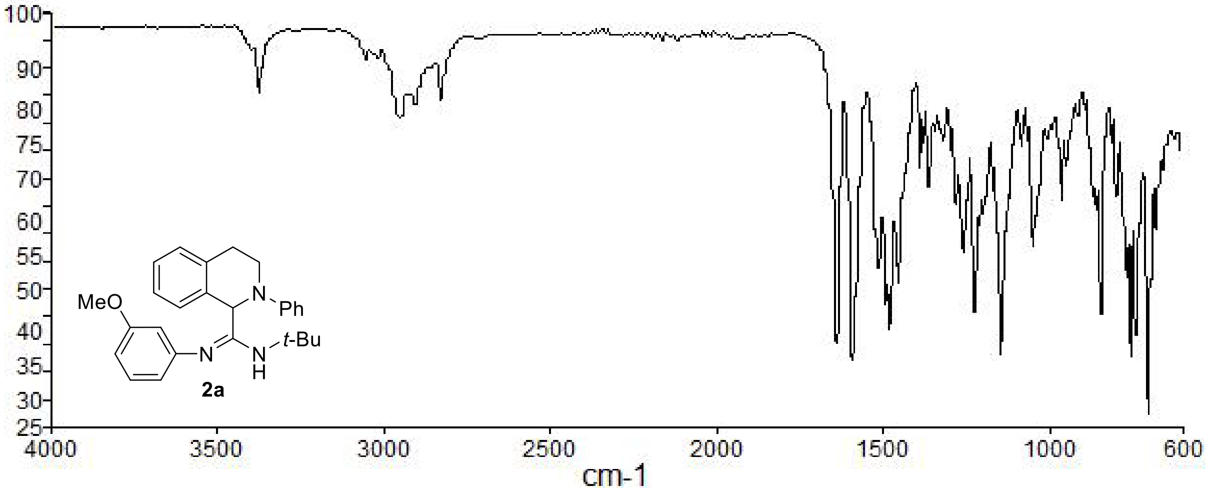

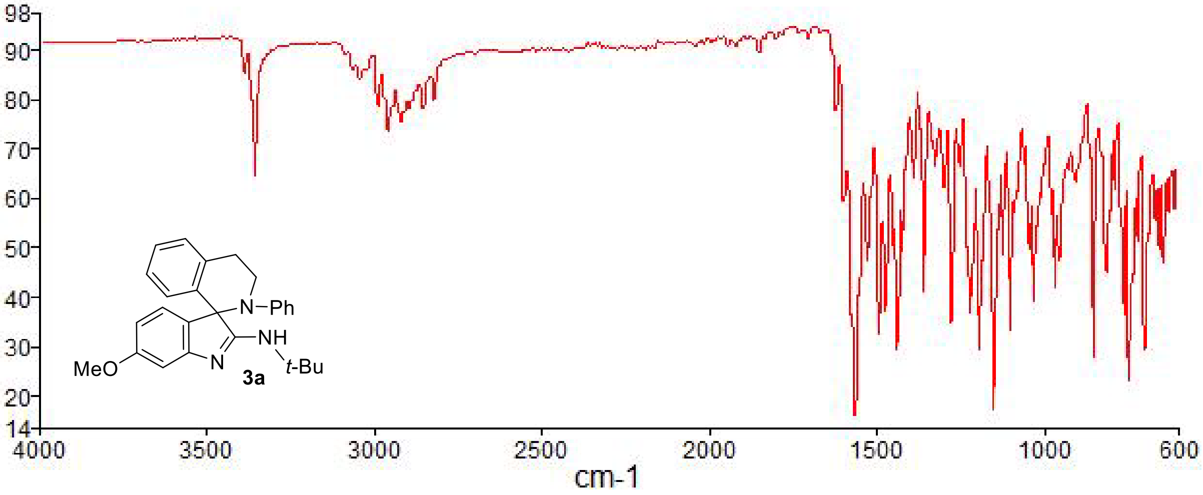

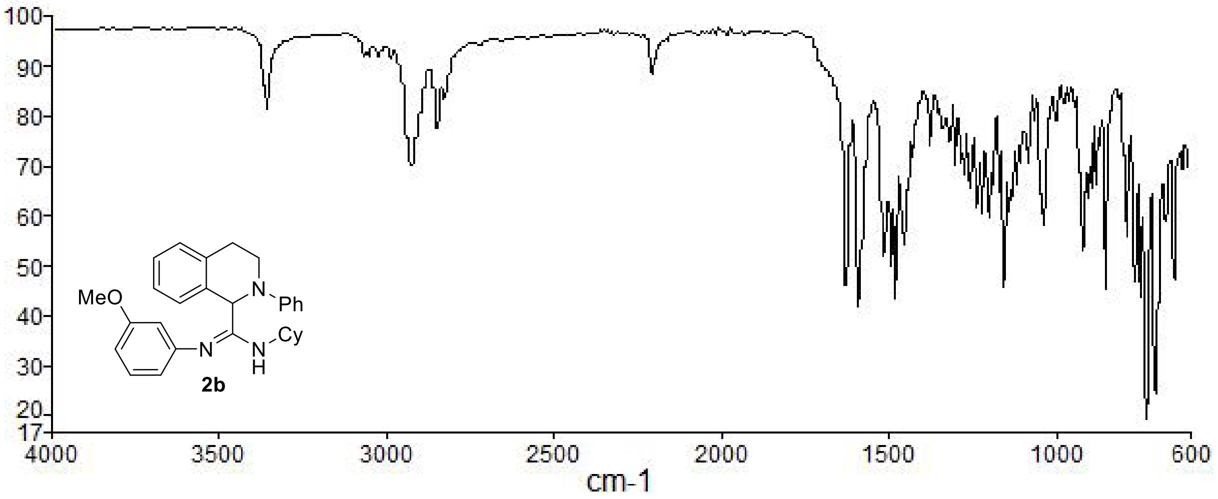

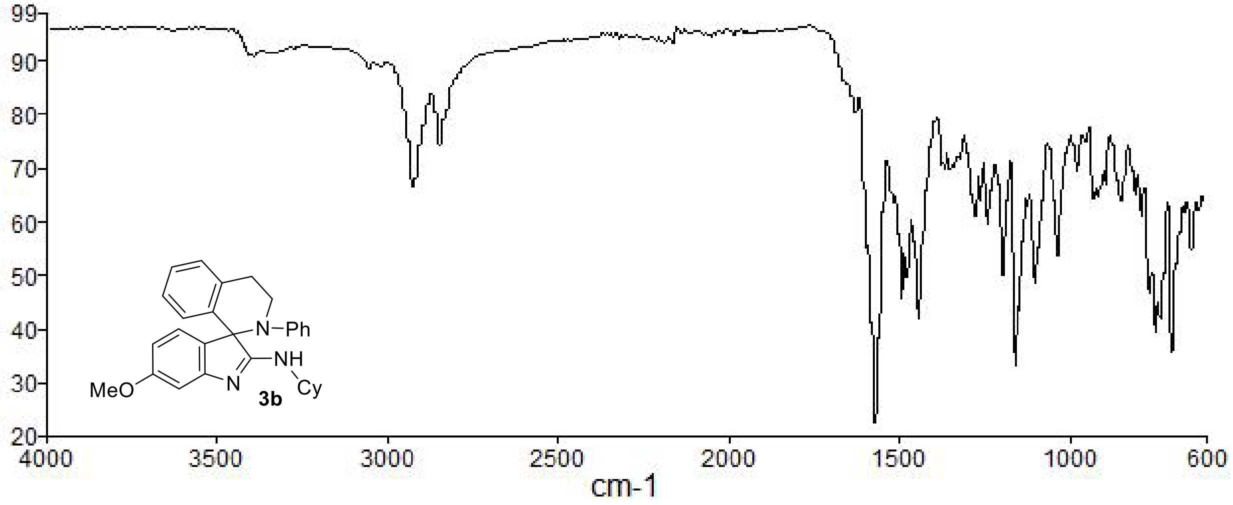
99
90
80
70
60
50
40
30
20
4000
3500
3000
2500
2000
1500
1000
600
cm-1
N–Ph
NH
Cy
MeO
N
3b

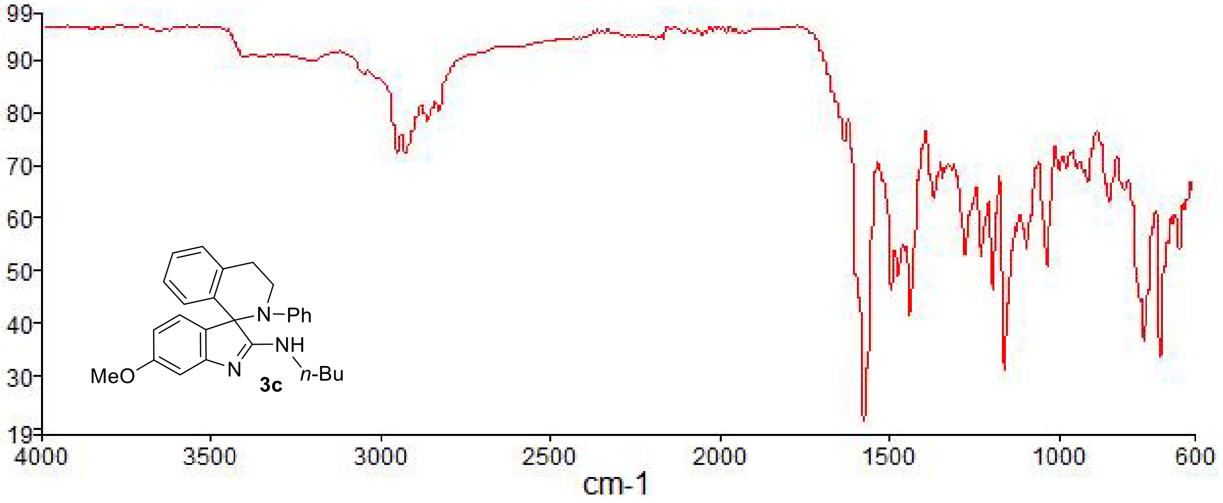
99
90
80
70
60
50
40
30
19
4000
3500
3000
2500
2000
1500
1000
600
cm-1
N–Ph
NH
n-Bu
MeO
N
3c

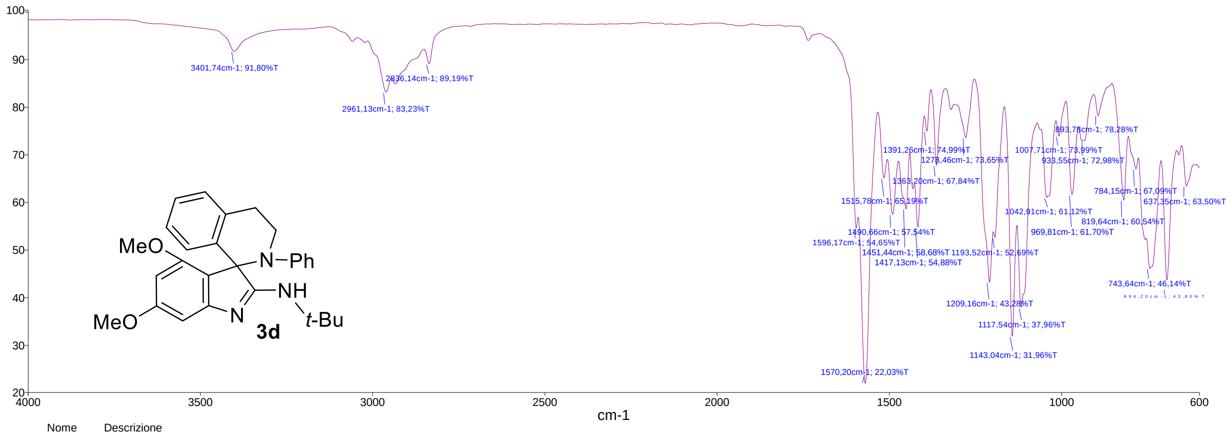
100
90
80
70
60
50
40
30
20
4000
3500
3000
2500
2000
1500
1000
600
cm-1
3401,74cm-1; 91,80%T
2836,14cm-1; 89,19%T
2961,13cm-1; 83,23%T
893,78cm-1; 78,28%T
1391,26cm-1; 74,99%T
1007,71cm-1; 73,99%T
1278,46cm-1; 73,65%T
933,55cm-1; 72,98%T
1363,20cm-1; 67,84%T
784,15cm-1; 67,09%T
1515,78cm-1; 65,19%T
637,35cm-1; 63,50%T
1042,91cm-1; 61,12%T
819,64cm-1; 60,54%T
1490,66cm-1; 57,54%T
969,81cm-1; 61,70%T
1596,17cm-1; 54,65%T
1451,44cm-1; 58,68%T
1193,52cm-1; 52,69%T
1417,13cm-1; 54,88%T
743,64cm-1; 46,14%T
694,20cm-1; 43,80%T
1209,16cm-1; 43,28%T
1117,54cm-1; 37,96%T
1143,04cm-1; 31,96%T
1570,20cm-1; 22,03%T
MeO
N–Ph
NH
t-Bu
MeO
N
3d
Nome
Descrizione

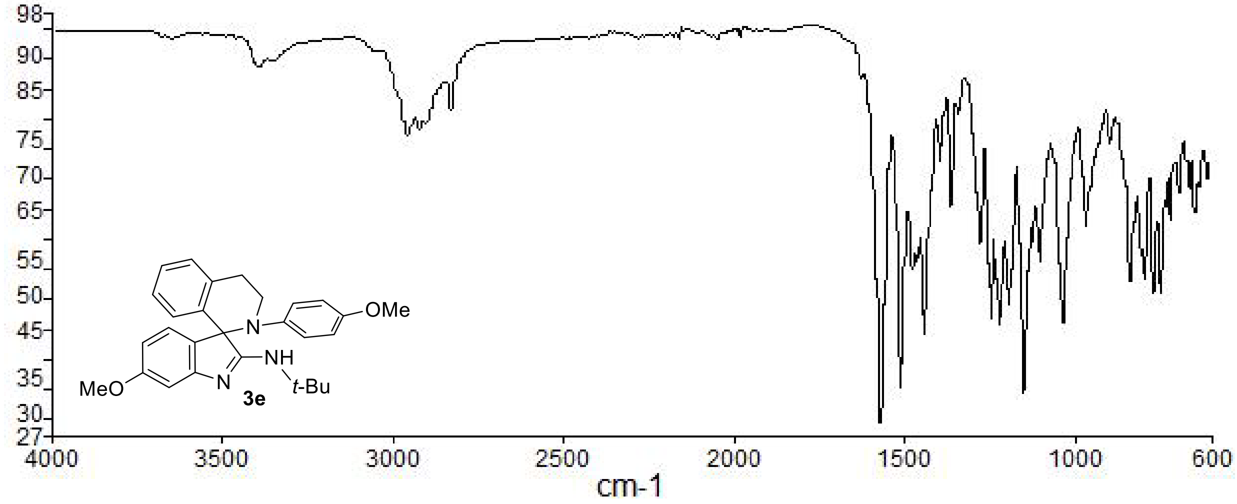
OMe
N
NH
MeO
N
t-Bu
3e
cm-1

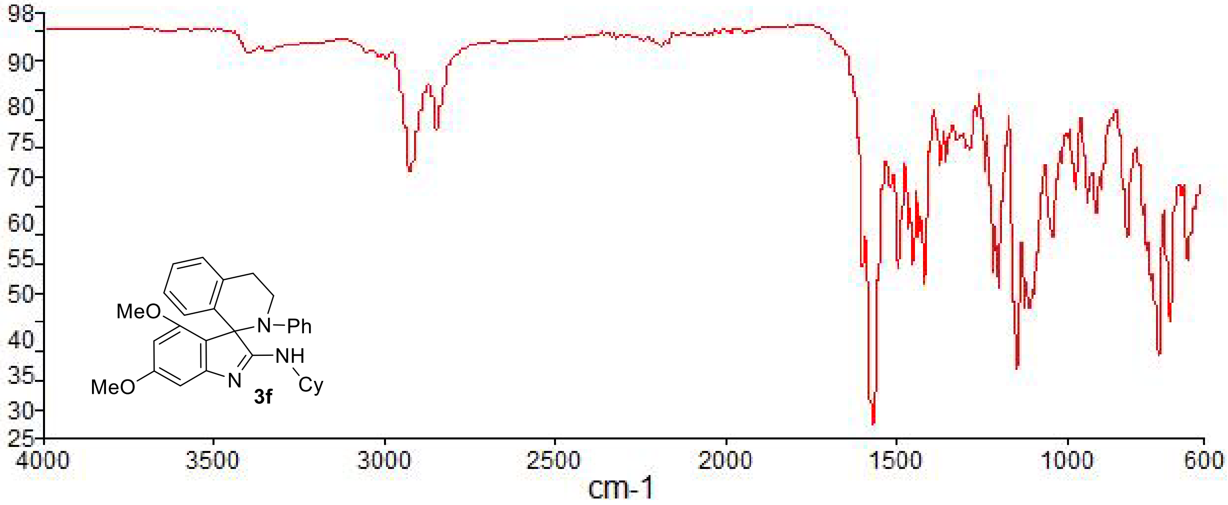
MeO
N–Ph
NH
MeO
N
Cy
3f
cm-1

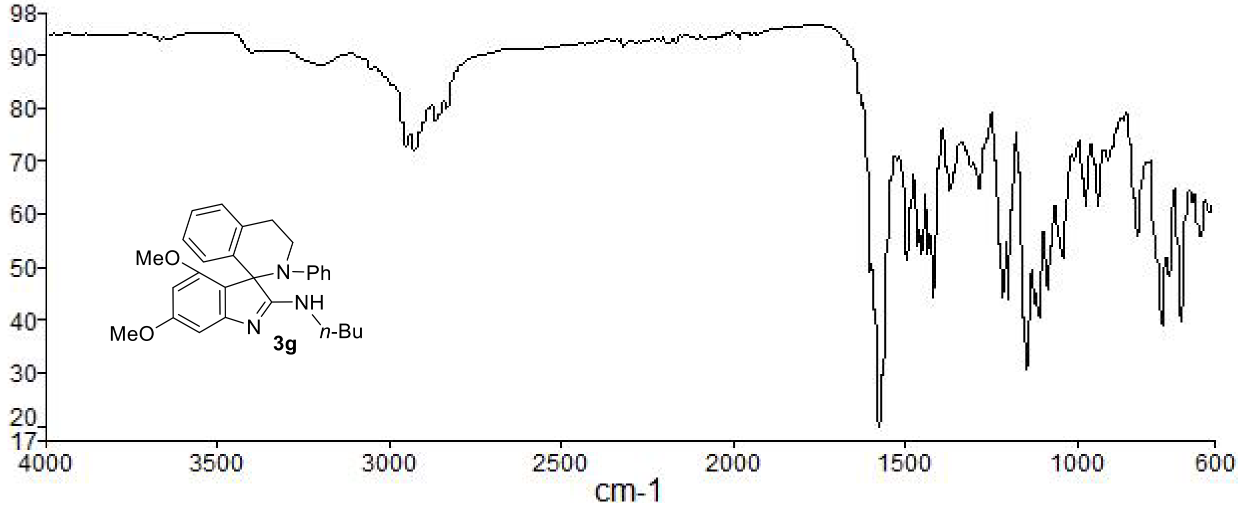
MeO
N–Ph
NH
MeO
N
n-Bu
3g
cm-1

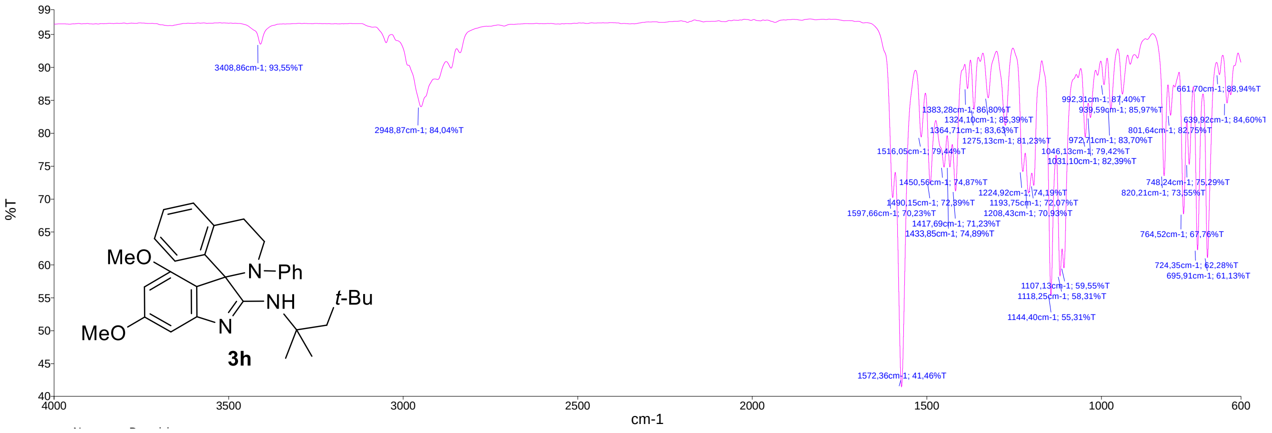
%T
cm-1
3408,86cm-1; 93,55%T
2948,87cm-1; 84,04%T
1383,28cm-1; 86,80%T
1324,10cm-1; 85,39%T
1364,71cm-1; 83,63%T
1275,13cm-1; 81,23%T
1516,05cm-1; 79,44%T
1450,56cm-1; 74,87%T
1224,92cm-1; 74,19%T
1490,15cm-1; 72,39%T
1193,75cm-1; 72,07%T
1597,66cm-1; 70,23%T
1208,43cm-1; 70,93%T
1417,69cm-1; 71,23%T
1433,85cm-1; 74,89%T
992,31cm-1; 87,40%T
939,59cm-1; 85,97%T
972,71cm-1; 83,70%T
1046,13cm-1; 79,42%T
1031,10cm-1; 82,39%T
661,70cm-1; 88,94%T
639,92cm-1; 84,60%T
801,64cm-1; 82,75%T
748,24cm-1; 75,29%T
820,21cm-1; 73,55%T
764,52cm-1; 67,76%T
724,35cm-1; 62,28%T
695,91cm-1; 61,13%T
1107,13cm-1; 59,55%T
1118,25cm-1; 58,31%T
1144,40cm-1; 55,31%T
1572,36cm-1; 41,46%T
MeO
MeO
N−Ph
NH
t-Bu
N
3h

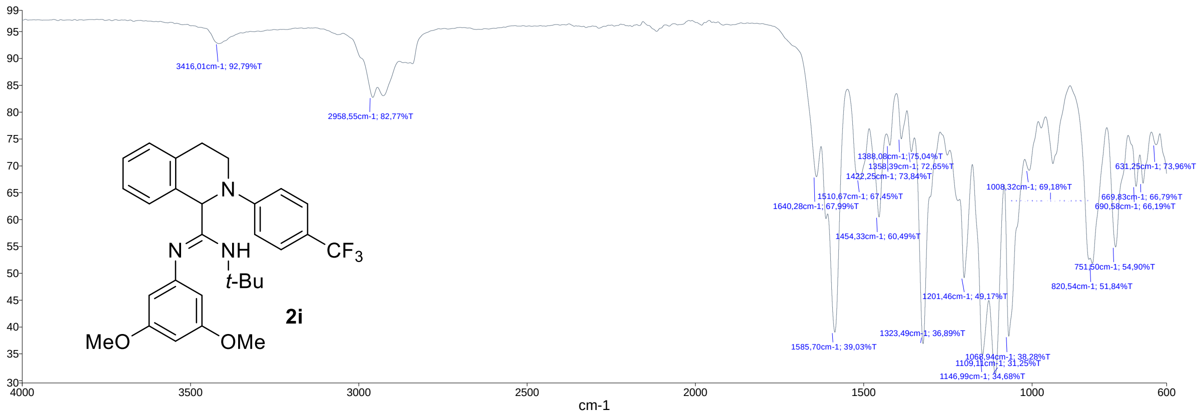
cm-1
3416,01cm-1; 92,79%T
2958,55cm-1; 82,77%T
1388,08cm-1; 75,04%T
1358,39cm-1; 72,65%T
1422,25cm-1; 73,84%T
1510,67cm-1; 67,45%T
1640,28cm-1; 67,99%T
1454,33cm-1; 60,49%T
1008,32cm-1; 69,18%T
631,25cm-1; 73,96%T
669,83cm-1; 66,79%T
690,58cm-1; 66,19%T
751,50cm-1; 54,90%T
820,54cm-1; 51,84%T
1201,46cm-1; 49,17%T
1323,49cm-1; 36,89%T
1585,70cm-1; 39,03%T
1068,94cm-1; 38,28%T
1109,11cm-1; 31,25%T
1146,99cm-1; 34,68%T
N
N
NH
t-Bu
CF3
MeO
OMe
2i

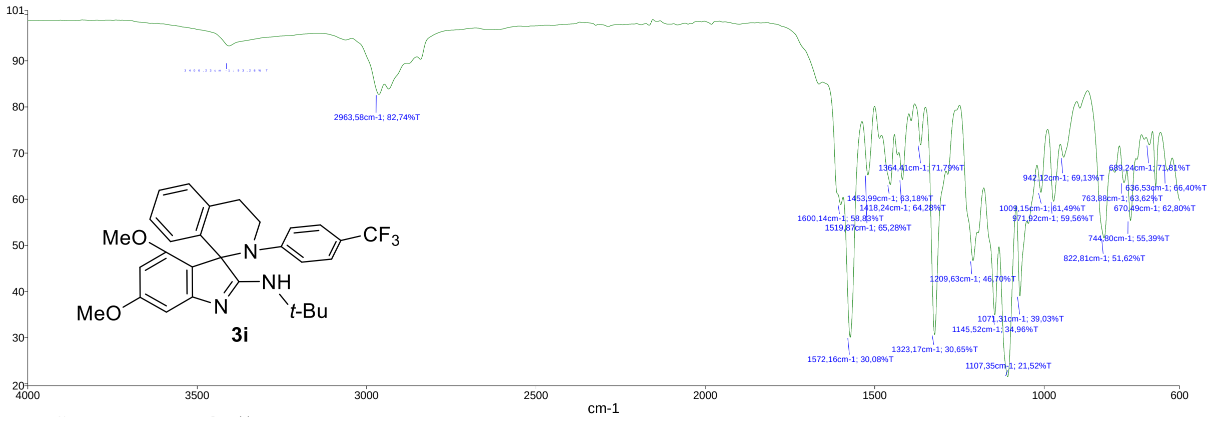
cm-1
2963,58cm-1; 82,74%T
1364,41cm-1; 71,79%T
1453,99cm-1; 63,18%T
1418,24cm-1; 64,26%T
1600,14cm-1; 58,83%T
1519,87cm-1; 65,28%T
942,12cm-1; 69,13%T
1009,15cm-1; 61,49%T
971,92cm-1; 59,56%T
689,24cm-1; 71,81%T
636,53cm-1; 66,40%T
763,88cm-1; 63,62%T
670,49cm-1; 62,80%T
744,80cm-1; 55,39%T
822,81cm-1; 51,62%T
1209,63cm-1; 46,70%T
1071,31cm-1; 39,03%T
1145,52cm-1; 34,96%T
1323,17cm-1; 30,65%T
1572,16cm-1; 30,08%T
1107,35cm-1; 21,52%T
MeO
MeO
N
CF3
NH
t-Bu
N
3i

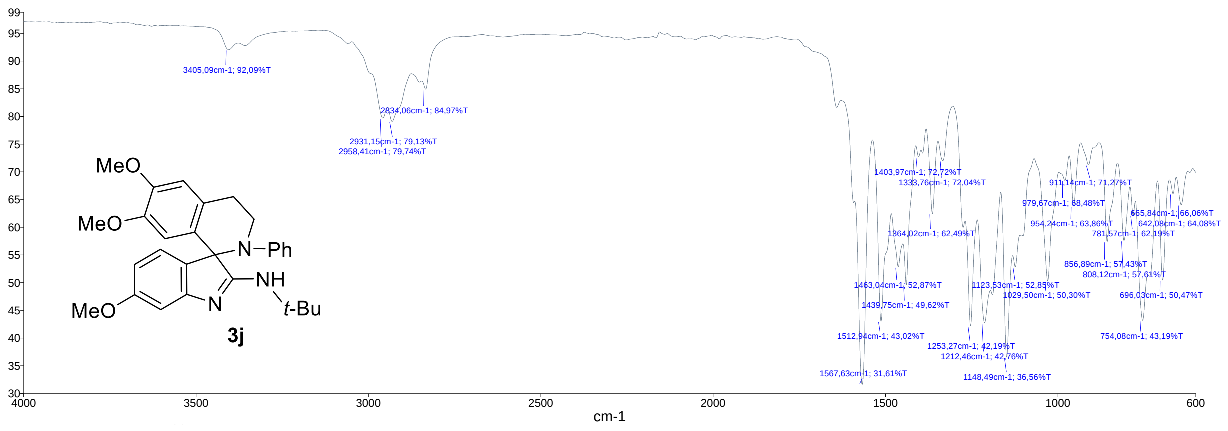
cm-1
3405,09cm-1; 92,09%T
2834,06cm-1; 84,97%T
2931,15cm-1; 79,13%T
2958,41cm-1; 79,74%T
1403,97cm-1; 72,72%T
1333,76cm-1; 72,04%T
1364,02cm-1; 62,49%T
1463,04cm-1; 52,87%T
1439,75cm-1; 49,62%T
1512,94cm-1; 43,02%T
1567,63cm-1; 31,61%T
1123,53cm-1; 52,85%T
1029,50cm-1; 50,30%T
1253,27cm-1; 42,19%T
1212,46cm-1; 42,76%T
1148,49cm-1; 36,56%T
911,14cm-1; 71,27%T
979,67cm-1; 68,48%T
954,24cm-1; 63,86%T
665,84cm-1; 66,06%T
642,08cm-1; 64,08%T
781,57cm-1; 62,19%T
856,89cm-1; 57,43%T
808,12cm-1; 57,61%T
696,03cm-1; 50,47%T
754,08cm-1; 43,19%T
MeO
MeO
MeO
N−Ph
NH
t-Bu
N
3j

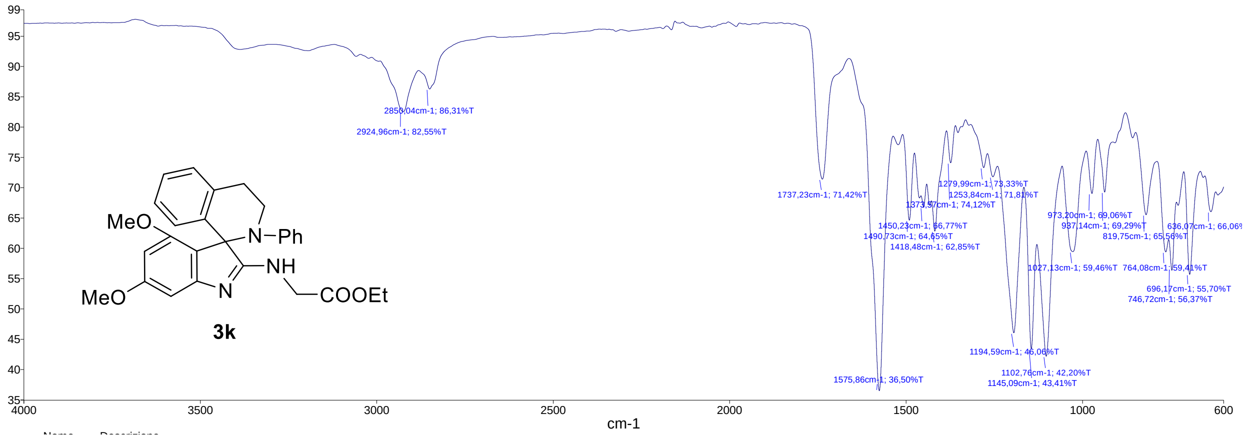

2859,04cm-1; 86,31%T
2924,96cm-1; 82,55%T
1737,23cm-1; 71,42%T
1279,99cm-1; 73,33%T
1253,84cm-1; 71,81%T
1373,57cm-1; 74,12%T
1450,23cm-1; 66,77%T
1490,73cm-1; 64,65%T
1418,48cm-1; 62,85%T
973,20cm-1; 69,06%T
937,14cm-1; 69,29%T
819,75cm-1; 65,56%T
1027,13cm-1; 59,46%T
764,08cm-1; 59,41%T
696,17cm-1; 55,70%T
746,72cm-1; 56,37%T
1194,59cm-1; 46,06%T
1102,76cm-1; 42,20%T
1145,09cm-1; 43,41%T
1575,86cm-1; 36,50%T
cm-1
MeO
N−Ph
NH
MeO
N
COOEt
3k

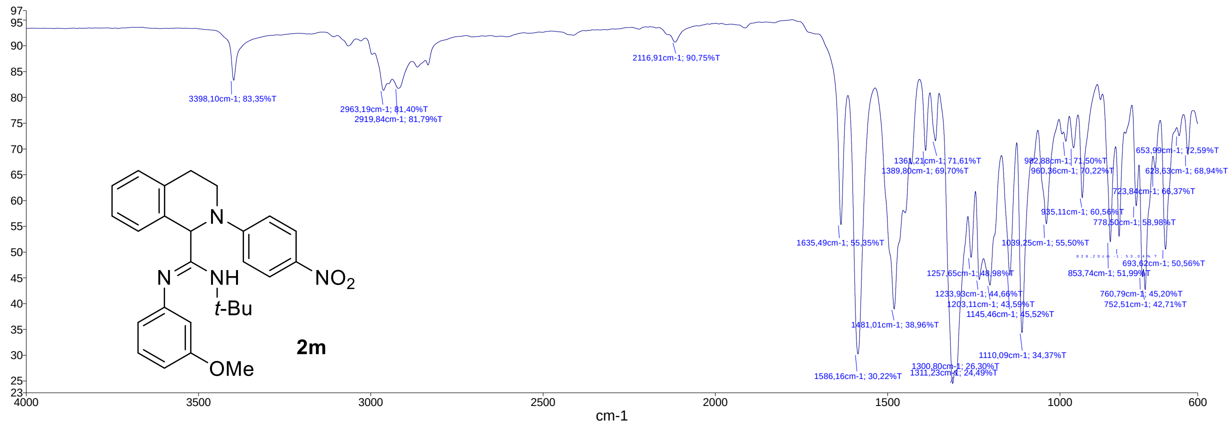

2116,91cm-1; 90,75%T
3398,10cm-1; 83,35%T
2963,19cm-1; 81,40%T
2919,84cm-1; 81,79%T
1361,21cm-1; 71,61%T
1389,80cm-1; 69,70%T
982,88cm-1; 71,50%T
960,36cm-1; 70,22%T
653,99cm-1; 72,59%T
628,63cm-1; 68,94%T
723,84cm-1; 66,37%T
935,11cm-1; 60,56%T
778,50cm-1; 58,98%T
1635,49cm-1; 55,35%T
1039,25cm-1; 55,50%T
693,62cm-1; 50,56%T
1257,65cm-1; 48,98%T
853,74cm-1; 51,99%T
1233,93cm-1; 44,66%T
1203,11cm-1; 43,59%T
1145,46cm-1; 45,52%T
760,79cm-1; 45,20%T
752,51cm-1; 42,71%T
1481,01cm-1; 38,96%T
1110,09cm-1; 34,37%T
1300,80cm-1; 26,30%T
1311,23cm-1; 24,49%T
1586,16cm-1; 30,22%T
cm-1
N
NO2
N
NH
t-Bu
OMe
2m

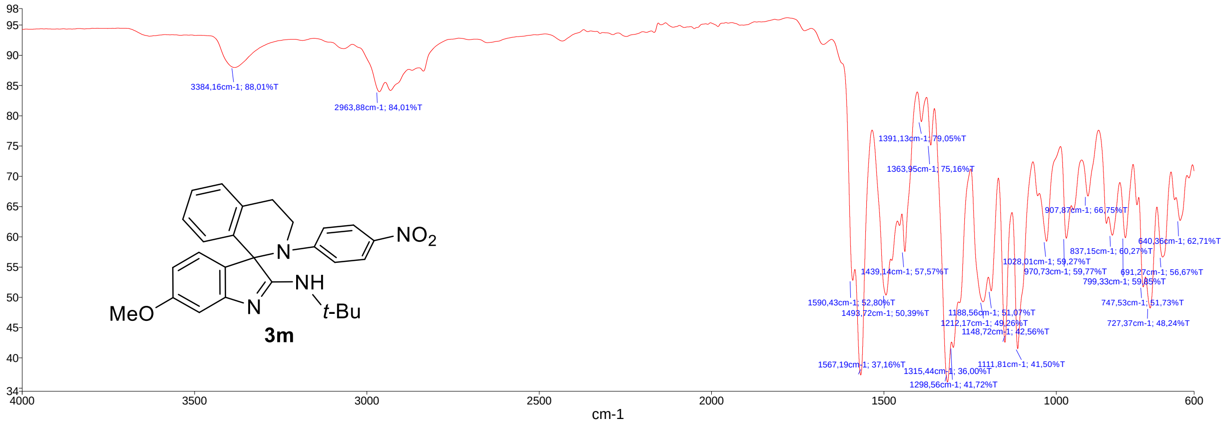

3384,16cm-1; 88,01%T
2963,88cm-1; 84,01%T
1391,13cm-1; 79,05%T
1363,95cm-1; 75,16%T
907,87cm-1; 66,75%T
640,36cm-1; 62,71%T
837,15cm-1; 60,27%T
1028,01cm-1; 59,27%T
970,73cm-1; 59,77%T
691,27cm-1; 56,67%T
799,33cm-1; 59,85%T
1439,14cm-1; 57,57%T
1590,43cm-1; 52,80%T
1493,72cm-1; 50,39%T
1188,56cm-1; 51,07%T
1212,17cm-1; 49,26%T
1148,72cm-1; 42,56%T
747,53cm-1; 51,73%T
727,37cm-1; 48,24%T
1567,19cm-1; 37,16%T
1315,44cm-1; 36,00%T
1111,81cm-1; 41,50%T
1298,56cm-1; 41,72%T
cm-1
N
NO2
NH
MeO
N
t-Bu
3m

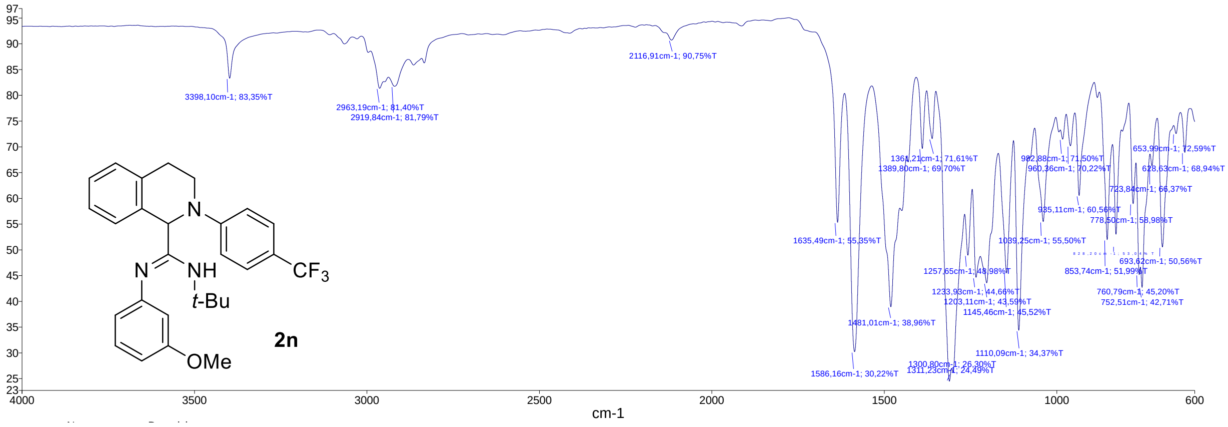

2116,91cm-1; 90,75%T
3398,10cm-1; 83,35%T
2963,19cm-1; 81,40%T
2919,84cm-1; 81,79%T
1361,21cm-1; 71,61%T
1389,80cm-1; 69,70%T
982,88cm-1; 71,50%T
960,36cm-1; 70,22%T
653,99cm-1; 72,59%T
628,63cm-1; 68,94%T
723,84cm-1; 66,37%T
935,11cm-1; 60,56%T
778,50cm-1; 58,98%T
1635,49cm-1; 55,35%T
1039,25cm-1; 55,50%T
693,62cm-1; 50,56%T
1257,65cm-1; 48,98%T
853,74cm-1; 51,99%T
1233,93cm-1; 44,66%T
1203,11cm-1; 43,59%T
1145,46cm-1; 45,52%T
760,79cm-1; 45,20%T
752,51cm-1; 42,71%T
1481,01cm-1; 38,96%T
1110,09cm-1; 34,37%T
1300,80cm-1; 26,30%T
1311,23cm-1; 24,49%T
1586,16cm-1; 30,22%T
cm-1
N
CF3
N
NH
t-Bu
OMe
2n

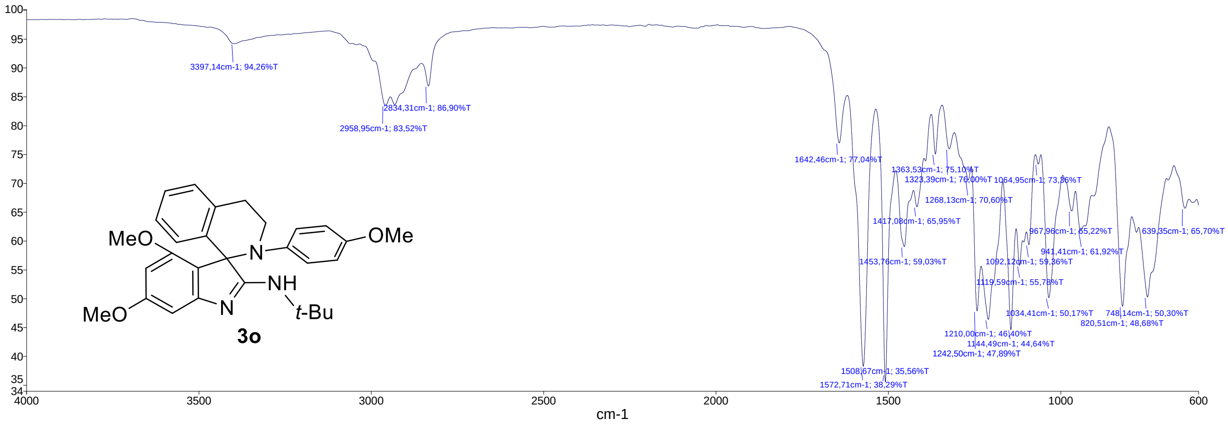
3397,14cm-1; 94,26%T
2834,31cm-1; 86,90%T
2958,95cm-1; 83,52%T
1642,46cm-1; 77,04%T
1508,67cm-1; 35,56%T
1572,71cm-1; 38,29%T
748,14cm-1; 50,30%T
820,51cm-1; 48,68%T
639,35cm-1; 65,70%T
MeO
OMe
NH
t-Bu
3o
cm-1

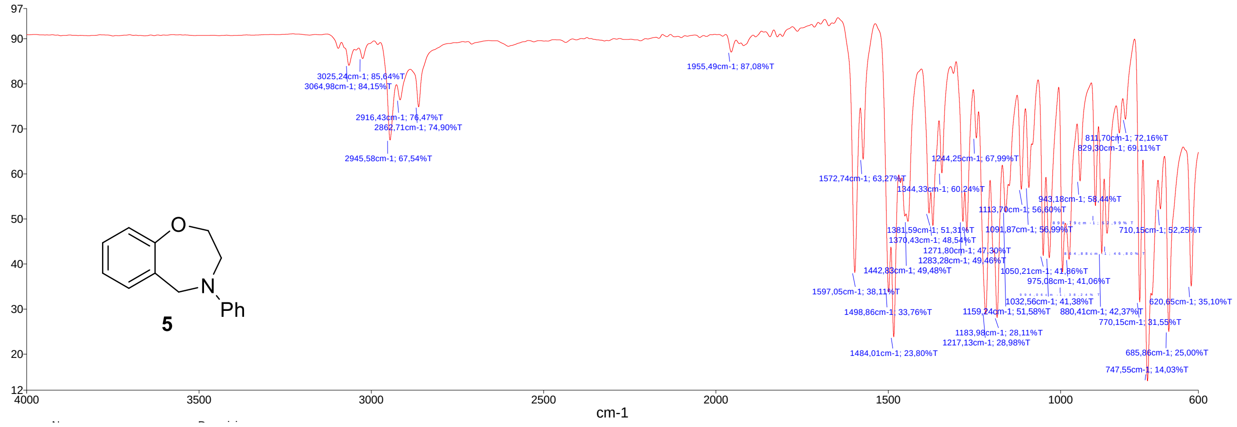
5
Ph
cm-1

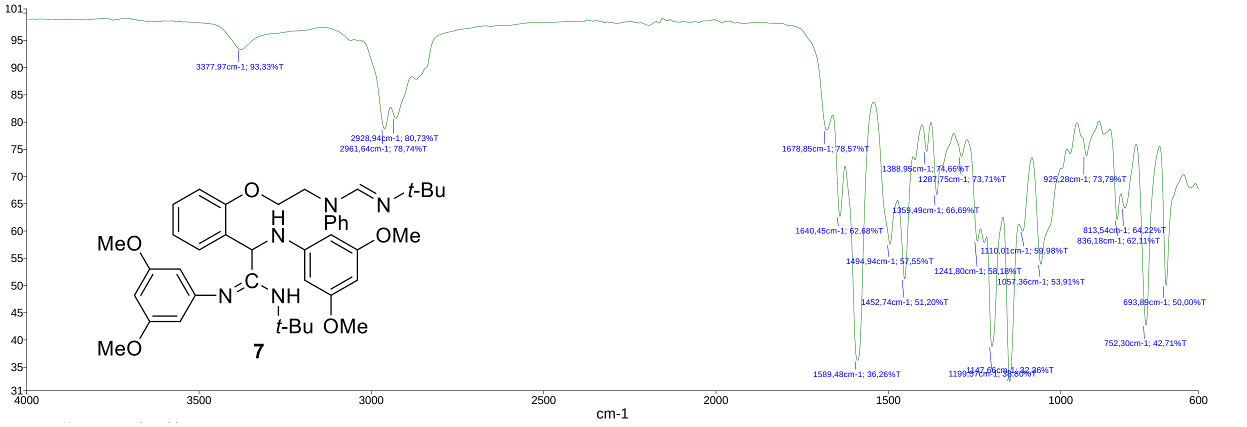
3377,97cm-1; 93,33%T
2928,94cm-1; 80,73%T
2961,64cm-1; 78,74%T
1678,85cm-1; 78,57%T
1640,45cm-1; 62,68%T
1589,48cm-1; 36,26%T
752,30cm-1; 42,71%T
693,89cm-1; 50,00%T
MeO
OMe
t-Bu
Ph
7
cm-1

Experimental procedures and spectroscopic characterization

Synthesis of 1,2,3,4-tetrahydroisoquinoline derivatives *N*-Ph-THIQ, S1, S2, S3, S4

General procedure: 1,2,3,4-Tetrahydroisoquinoline (1 equiv) and the opportune bromoarene (1.2 equiv) were added to a suspension of Pd(dba)$_2$ (palladium(0) bis(dibenzylideneacetone)) (0.03 equiv), XPhos (0.06 equiv) and sodium *tert*-butoxide (3.3 equiv) in anhydrous and deoxygenated toluene (0.1 M), and the resulting mixture was refluxed under nitrogen for 20 h. After cooling to room temperature, the mixture was filtered first employing Büchner vacuum filtration and then through a celite cake washing with EtOAc. The solvent was removed under reduced pressure and the residue was purified by column chromatography on silica gel, using the opportune eluent.

2-Phenyl-1,2,3,4-tetrahydroisoquinoline (*N*-Ph-THIQ)

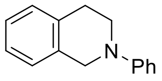

The physical and spectral data agreed with those reported.[2]

2-(4-(Trifluoromethyl)phenyl)-1,2,3,4-tetrahydroisoquinoline S1

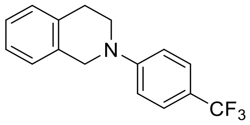

The physical and spectral data agreed with those reported.[2]

2-(4-Methoxyphenyl)-1,2,3,4-tetrahydroisoquinoline S3

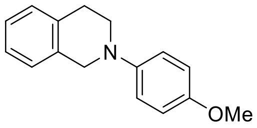

The physical and spectral data agreed with those reported.[2]

6,7-Dimethoxy-2-phenyl-1,2,3,4-tetrahydroisoquinoline S4

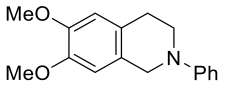

The physical and spectral data agreed with those reported.[3]

2-(4-Nitrophenyl)-1,2,3,4-tetrahydroisoquinoline S5

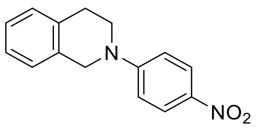

Compound **S5** was synthesized according to a literature procedure.[4]

Synthesis of compound 5

Compound **5** is synthesized starting from salicylic aldehyde through the following procedure:

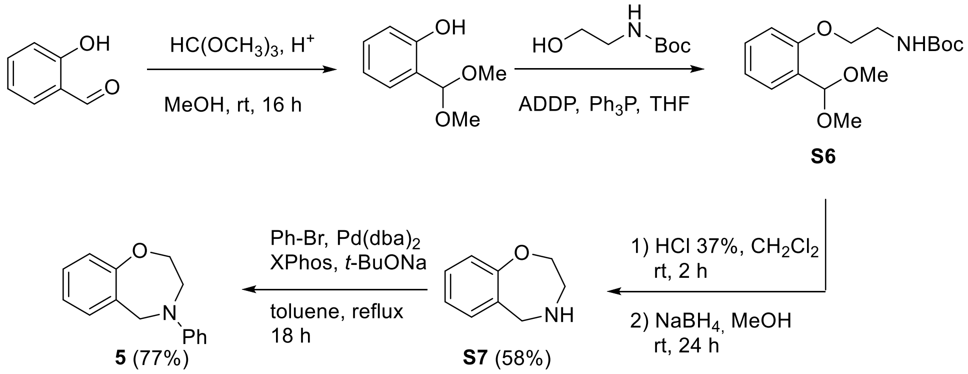

For preparation of **S6** see Reference [5]. Acetal **S6** (913 mg, 2.93 mmol) was dissolved in CH_2Cl_2 (12 mL) and treated with 37% aqueous HCl (2.45 mL, 29.3 mmol). The mixture was stirred at rt for 2 h. Then it was diluted with CH_2Cl_2 (10 mL), and cautiously treated with 5% aqueous Na_2CO_3 (15 mL). After checking that pH > 9, the two phases were separated, and the aqueous phase was extracted with CH_2Cl_2 (3 × 20 mL). Then, the collected organic phases were washed with brine, dried with Na_2SO_4, filtered and evaporated. The resulting crude imine (oil) was taken up in dry methanol (6.0 mL), and treated with $NaBH_4$ (213 mg, 5.63 mmol). The mixture was stirred at rt for 24 h and finally treated with saturated aqueous

Na_2CO_3 (20 mL). The resulting aqueous phase was extracted with EtOAc (15 mL × 6) and the collected organic phases were dried with Na_2SO_4, filtered and evaporated. The residue was chromatographed (EtOAc /MeOH + 2% Et_3N from 8:2 to 7:3) to give pure **S7** (243 mg, 58%) [6]. To a solution of **S7** (350 mg, 2.35 mmol, 2 equiv.), X-Phos (67 mg, 0.14 mmol, 0.06 equiv.), Pd(dba)$_2$ (41 mg, 0.07 mmol, 0.03 equiv), sodium *t*-butoxide (745 mg, 7.76 mmol, 3.3 equiv), in toluene (24 mL) under Ar, bromobenzene (300 µL, 2.82 mmol, 1.2 equiv) was added and the reaction mixture was stirred at reflux for 24 h. The reaction mixture was filtered through Celite, concentrated under reduced pressure and purified by flash column chromatography (PE/CH_2Cl_2 70:30+ 2% Et_3N) to give the **5** as a yellow oil (405 mg, 77%); **4-phenyl-2,3,4,5-tetrahydrobenzo[*f*][1,4]oxazepane 5**: Rf 0.29 (PE/CH_2Cl_2 70:30); I.R.: ῡ (cm^{-1}) = 3065, 3025, 2946, 2916, 2863, 1955, 1597, 1573, 1499, 1484, 1443, 1382, 1370, 1344, 1283, 1272, 1244, 1217, 1184, 1159, 1114, 1092, 1050, 1033, 994, 975, 943, 899, 880, 865, 829, 812, 770, 748, 710, 686, 621; ^{1}H NMR (400 MHz, $CDCl_3$) δ 7.30 (dd, *J* = 7.5, 1.7 Hz, 1H, H Ar), 7.22 – 7.09 (m, 3H, 3 H Ar), 7.04 – 6.95 (m, 2H, 2 H Ar), 6.82 (d, *J* = 8.3 Hz, 2H, 2 H Ar), 6.68 (td, *J* = 7.3, 1.1 Hz, 1H, H Ar), 4.59 (s, 2H, CH_2), 4.20 – 4.11 (m, 2H, CH_2 near O), 3.93 – 3.82 (m, 2H, CH_2 near N); ^{13}C NMR (101 MHz, $CDCl_3$) δ 159.5 (Cq near O), 147.8 (Cq near N), 131.4 (Cq Ar), 130.6 (CH Ar), 129.5 (2 CH Ar), 128.6 (CH Ar), 123.3 (CH Ar), 121.3 (CH Ar), 117.6 (CH Ar), 114.2 (2 CH Ar), 70.7 (CH_2), 53.6 (CH_2 near O), 53.2 (CH_2 near N); HRMS (ESI$^+$): m/z calcd for $C_{15}H_{16}NO$ $[M+H]^+$: 226.1226. Found: 226.1228.

During the optimization of the one-pot four step process, we isolated pure α-aminoamidine **2a** (12 mg, 12%, following reaction conditions of entry 1, Table 1) and pure α-aminoamide **4a** (19 mg, 26%, following reaction conditions of entry 1, Table 1).

_N_-(_tert_-Butyl)-_N'_-(3-methoxyphenyl)-2-phenyl-1,2,3,4-tetrahydroisoquinoline-1-carboximidamide (2a)

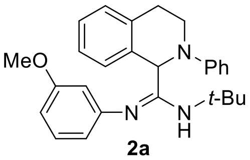

2a

Yellow oil; Rf 0.72 (PE/EtOAc 85:15); I.R.: ῡ (cm^{-1}) = 3380, 3056, 2959, 2831, 2119, 1637, 1590, 1512, 1490, 1479, 1449, 1387, 1375, 1361, 1355, 1322, 1294, 1278, 1260, 1252, 1222, 1204, 1192, 1164, 1145, 1075, 1063, 1042, 1003, 993, 961, 946, 910, 865, 850, 840, 806, 794, 783, 765, 753, 733, 696, 673, 655, 605; ^{1}H NMR (400 MHz, $CDCl_3$) δ 7.32 (dd, J = 8.7, 7.1 Hz, 2H, 2 CH Ar), 7.21 – 7.01 (m, 8H, 8 CH Ar), 6.46 (dd, J = 8.2, 1.7 Hz, 1H, CH Ar), 6.23 (dd, J = 7.9, 1.0 Hz, 1H, CH Ar), 6.17 (t, J = 2.2 Hz, 1H, CH Ar), 5.25 (s, 1H, CH), 4.97 (bs, 1H, NH), 3.67 (s, 3H, OCH_3), 3.47 (dt, J = 10.8, 4.5 Hz, 1H, 1 H of CH_2), 3.26 (td, J = 10.6, 3.4 Hz, 1H, 1 H of CH_2), 3.17 (ddd, J = 15.5, 10.3, 4.8 Hz, 1H, 1 H of CH_2), 2.82 (dt, J = 15.5, 3.7 Hz, 1H, 1 H of CH_2), 1.26 (s, 9H, 3 CH_3 of _t_-Bu); ^{13}C NMR (101 MHz, $CDCl_3$) δ 160.3 (Cq near O), 154.7 (Cq amidine), 152.0 (Cq near N), 150.8 (Cq near N), 135.2 (Cq Ar), 135.0 (Cq Ar), 129.4 (CH Ar), 129.1 (2 CH Ar), 128.3 (CH Ar), 128.1 (CH Ar), 127.1 (CH Ar), 126.2 (CH Ar), 122.7 (CH Ar), 120.9 (2 CH Ar), 114.5 (CH Ar), 107.5 (CH Ar), 107.5 (CH Ar), 61.1 (CHNH), 55.2 (OCH_3), 51.0 (CH_2), 49.0 (CH_2), 29.8 (Cq _t_-Bu), 28.4 (3 CH_3 of _t_-Bu); HRMS (ESI^+): m/z calcd for $C_{27}H_{32}N_3O$ $[M+H]^+$: 414.2540. Found: 414.2593.

N-(_tert_-Butyl)-2-phenyl-1,2,3,4-tetrahydroisoquinoline-1-carboxamide (4a)

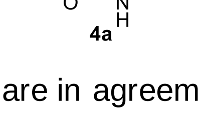

4a

The analytical and spectroscopic data are in agreement to what previously reported.[7]

Synthesis of spiro[indole-tetrahydroisoquinolines] 3

N-Cyclohexyl-6-methoxy-2'-phenyl-3',4'-dihydro-2'*H*-spiro[indole-3,1'-isoquinolin]-2-amine (3b)

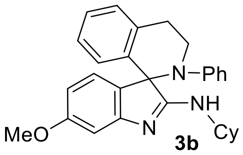

3b

A solution of *N*-phenyltetrahydroisoquinoline (1 equiv, 0.53 mmol, 111 mg), 3-methoxyaniline (1.5 equiv, 0.80 mmol, 89 µL) and $BrCCl_3$ (3 equiv, 1.59 mmol, 156 µL) in CH_3CN (2.7 mL, 0.2 M) in a vial was degassed with argon for 2 minutes, then cyclohexyl isocyanide (2.2 equiv, 1.17 mmol, 143 µL) was added and the vial sealed. The mixture was irradiated at 30 °C at 451 nm (blue LEDs) under magnetic stirring for 24 h. The reaction mixture was treated with Et_3N (3 equiv, 1.59 mmol, 221 µL), concentrated and the residue was purified by column chromatography on aluminium oxide with PE/AcOEt (from 12:1 + 1% of Et_3N to 9:1 + 1% of Et_3N) to give **2b** (127 mg, 54%) as yellow oil and **3b** (64 mg, 27%) as yellow oil; characterization of **2b**: Rf 0.77 (PE/AcOEt 9:1); I.R.: ῡ (cm^{-1}) = 3367, 3074, 3026, 2993, 2935, 2855, 2831, 1627, 1586, 1515, 1492, 1481, 1465, 1459, 1451, 1424, 1377, 1334, 1316, 1297, 1284, 1270, 1254, 1234, 1220, 1206, 1197, 1188, 1166, 1153, 1142, 1132, 1117, 1104, 1082, 1063, 1039, 1002, 993, 973, 956, 945, 917, 900, 893, 880, 873, 865, 852, 842, 785, 766, 761, 745, 727, 700, 668, 642, 617, 603; ^{1}H NMR (400 MHz, DMSO-d_6) δ 7.29 – 7.23 (m, 2H, 2 H Ar), 7.21 – 7.12 (m, 4H, 4 H Ar), 6.99 – 6.90 (m, 3H, 3 H Ar), 6.84 (t, J = 7.2 Hz, 1H, H Ar), 6.35 (d, J = 7.6 Hz, 1H, H Ar), 5.88 (d, J = 7.7 Hz, 1H, H Ar), 5.81 (bs, 1H, NH), 5.31 (s, 1H, CH of THIQ), 3.51 (s, 3H, OCH_3), 3.50 (bs, 1H, <u>CH</u>NH), 3.26 – 3.15 (m, 2H, CH_2 of THIQ), 2.98 – 2.83 (m, 1H, 1 H of CH_2 THIQ), 2.73 (dt, J = 15.0, 4.4 Hz, 1H, 1 H of CH_2 THIQ), 1.81 – 1.66 (m, 2H, 2 H of Cy), 1.65 – 1.44 (m, 3H, 3 H of Cy), 1.20 – 0.99 (m, 5H, 5 H of Cy); ^{13}C NMR (101 MHz, DMSO-d_6) δ 159.5 (Cq near O), 155.2 (Cq amidine), 152.2 (Cq near N), 149.6 (Cq near N), 135.2 (Cq Ar), 134.5 (Cq Ar),

128.8 (CH Ar), 128.6 (2 CH Ar), 128.0 (CH Ar), 126.9 (CH Ar), 126.8 (CH Ar), 125.8 (CH Ar), 119.2 (CH Ar), 116.8 (2 CH Ar), 113.7 (CH Ar), 106.8 (CH Ar), 106.4 (CH Ar), 60.6 (CH of THIQ), 54.5 (OCH_3), 48.9 (CH of Cy), 32.1 (CH_2 of THIQ), 31.9 (CH_2 of Cy), 28.8 (CH_2 of Cy), 25.3 (CH_2 of THIQ), 24.7 (CH_2 of Cy), 24.6 (CH_2 of Cy); HRMS (ESI$^+$): m/z calcd for $C_{29}H_{34}N_3O$ [M+H]$^+$: 440.2696. Found: 440.2693. Characterization of **3b**; Rf 0.46 (PE/AcOEt 9:1); I.R.: ῡ (cm^{-1}) = 3400, 2929, 2852, 2051, 1626, 1568, 1516, 1492, 1476, 1439, 1361, 1349, 1275, 1258, 1236, 1193, 1155, 1122, 1096, 1030, 976, 925, 910, 891, 843, 804, 783, 761, 746, 728, 696, 659, 639, 616; ^{1}H NMR (400 MHz, $CDCl_3$) δ 7.20 (d, J = 7.6 Hz, 1H, H Ar), 7.15 (td, J = 7.3, 1.3 Hz, 1H, H Ar), 7.10 – 7.05 (m, 2H, 2 H Ar), 7.05 – 6.97 (m, 2H, 2 H Ar), 6.93 – 6.85 (m, 3H, 3 H Ar), 6.78 (d, J = 2.4 Hz, 1H, H Ar), 6.67 (d, J = 8.0 Hz, 1H, H Ar), 6.32 (dd, J = 8.1, 2.4 Hz, 1H, H Ar), 4.75 (bd, J = 8.6 Hz, 1H, NH), 3.98 (ddd, J = 11.9, 10.8, 3.6 Hz, 1H, 1 H of CH_2 THIQ), 3.86 – 3.77 (m, 1H, <u>CH</u>NH), 3.76 (s, 3H, OCH_3), 3.58 (ddd, J = 11.9, 5.4, 3.0 Hz, 1H, 1 H of CH_2 THIQ), 3.32 (ddd, J = 16.1, 10.8, 5.4 Hz, 1H, 1 H of CH_2 THIQ), 3.04 (dt, J = 16.0, 3.3 Hz, 1H, 1 H of CH_2 THIQ), 1.90 – 1.52 (m, 7H, 7 H of Cy), 1.40 – 1.27 (m, 1H, H of Cy), 1.12 – 0.91 (m, 2H, 2 H of Cy); ^{13}C NMR (101 MHz, $CDCl_3$) δ 176.4 (Cq amidine), 160.9 (Cq near O), 157.4 (Cq near N), 149.5 (Cq near N), 136.5 (Cq Ar), 134.0 (Cq Ar), 132.9 (Cq Ar), 129.0 (CH Ar), 128.6 (2 CH Ar), 127.0 (CH Ar), 127.0 (CH Ar), 126.7 (CH Ar), 123.7 (CH Ar), 123.5 (CH Ar), 123.3 (2 CH Ar), 107.0 (CH Ar), 103.3 (CH Ar), 75.3 (Cq spiro), 55.4 (OCH_3), 51.0 (CH of Cy), 47.8 (CH_2 of THIQ), 33.0 (CH_2 of Cy), 32.8 (CH_2 of Cy), 30.9 (CH_2 of THIQ), 25.6 (CH_2 of Cy), 24.9 (CH_2 of Cy), 24.8 (CH_2 of Cy); HRMS (ESI$^+$): m/z calcd for $C_{29}H_{32}N_3O$ [M+H]$^+$: 438.2540. Found: 438.2517.

***N*-Butyl-6-methoxy-2'-phenyl-3',4'-dihydro-2'*H*-spiro[indole-3,1'-isoquinolin]-2-amine (3c)**

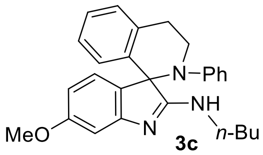

3c

A solution of *N*-phenyltetrahydroisoquinoline (1 equiv, 0.48 mmol, 100 mg), 3-methoxyaniline (1.5 equiv, 0.72 mmol, 81 µL) and $BrCCl_3$ (3 equiv, 1.44 mmol, 140 µL) in CH_3CN (2.4 mL, 0.2 M) in a vial was degassed with argon for 2 minutes, then *n*-butyl isocyanide (2.2 equiv, 1.06 mmol, 111 µL) was added and the vial sealed. The mixture was irradiated at 30 °C at 451 nm (blue LEDs) under magnetic stirring for 24 h. The reaction mixture was treated with Et_3N (3 equiv, 1.44 mmol, 200 µL), concentrated and the residue was purified by column chromatography on aluminium oxide with PE/Et_2O (from 3:1 + 1% of MeOH to 3:2 + 1% of MeOH) to give **3c** (105 mg, 53%) as brown oil; Rf 0.13 (PE/AcOEt 85:15); I.R.: ῡ (cm^{-1}) = 2956, 2931, 2870, 2834, 2187, 1631, 1571, 1491, 1475, 1464, 1438, 1362, 1339, 1275, 1223, 1190, 1155, 1094, 1029, 994, 975, 911, 843, 803, 760, 745, 695, 658, 638, 618; ^1H NMR (400 MHz, $CDCl_3$) δ 7.20 (d, *J* = 7.5 Hz, 1H, H Ar), 7.15 (t, *J* = 7.3 Hz, 1H, H Ar), 7.11 – 7.05 (m, 2H, 2 H Ar), 7.02 (d, *J* = 8.1 Hz, 2H, 2 H Ar), 6.93 – 6.83 (m, 3H, 3 H Ar), 6.81 – 6.76 (m, 1H, H Ar), 6.67 (d, *J* = 8.0 Hz, 1H, H Ar), 6.33 (dd, *J* = 8.1, 2.4 Hz, 1H, H Ar), 4.89 (bs, 1H, NH), 3.98 (td, *J* = 11.7, 3.4 Hz, 1H, 1 H of CH_2 THIQ), 3.76 (s, 3H, OCH_3), 3.64 – 3.56 (m, 1H, 1 H of CH_2 THIQ), 3.44 – 3.26 (m, 3H, 1 H of CH_2 THIQ and CH_2 of *n*-Bu), 3.04 (dt, *J* = 16.1, 3.2 Hz, 1H, 1 H of CH_2 THIQ), 1.46 – 1.35 (m, 2H, CH_2 of *n*-Bu), 1.19 – 1.09 (m, 2H, CH_2 of *n*-Bu), 0.80 (t, *J* = 7.4 Hz, 3H, CH_3 of *n*-Bu); ^{13}C NMR (101 MHz, $CDCl_3$) 177.5 (Cq of amidine), 161.0 (Cq near O), 157.3 (Cq near N), 149.5 (Cq near N), 136.3 (Cq Ar), 134.1 (Cq Ar), 132.9 (Cq Ar), 129.0 (CH Ar), 128.6 (2 CH Ar), 127.1 (CH Ar), 127.0 (CH Ar), 126.9 (CH Ar), 123.7 (CH Ar), 123.4 (CH Ar), 123.1 (2 CH Ar), 107.1 (CH Ar), 103.5 (CH Ar), 75.3 (Cq spiro), 55.4 (OCH_3), 47.8 (CH_2 of THIQ), 42.5 (CH_2 of *n*-Bu), 31.2 (CH_2 of THIQ), 31.0 (CH_2 of *n*-Bu), 20.0 (CH_2 of *n*-Bu), 13.8 (CH_3 of *n*-Bu); HRMS (ESI^+): m/z calcd for $C_{27}H_{30}N_3O$ $[M+H]^+$: 412.2383. Found: 412.2389.

_N_-(_tert_-Butyl)-4,6-dimethoxy-2'-phenyl-3',4'-dihydro-2'_H_-spiro[indole-3,1'-isoquinolin]-2-amine (3d)

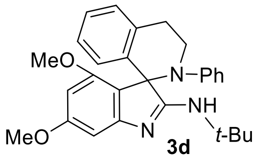

A solution of _N_-phenyltetrahydroisoquinoline (1 equiv, 0.55 mmol, 115 mg), 3,5-dimethoxyaniline (1.5 equiv, 0.82 mmol, 126 mg) and $BrCCl_3$ (3 equiv, 1.65 mmol, 162 µL) in CH_3CN (2.8 mL, 0.2 M) in a vial was degassed with argon for 2 minutes, then _t_-butyl isocyanide (2.2 equiv, 1.21 mmol, 137 µL) was added and the vial sealed. The mixture was irradiated at 30 °C at 451 nm (blue LEDs) under magnetic stirring for 24 h. The reaction mixture was treated with Et_3N (3 equiv, 1.65 mmol, 230 µL), concentrated and the residue was purified by column chromatography on silica with PE/AcOEt (9:1 + 1% of Et_3N) to give **3d** (147 mg, 62%) as cream foam; Rf 0.24 (PE/AcOEt 9:1 + 1% of Et_3N); I.R.: ū (cm^{-1}) = 3402, 2961, 2836, 1596, 1570, 1516, 1491, 1451, 1417, 1391, 1363, 1278, 1209, 1194, 1143, 1118, 1043, 1008, 970, 934, 894, 820, 784, 744, 694, 637; ^1H NMR (400 MHz, $CDCl_3$) δ 7.17 (d, J = 7.3 Hz, 1H, H Ar), 7.13 (td, J = 7.2, 1.2 Hz, 1H, H Ar), 7.10 – 7.03 (m, 2H, 2 H Ar), 7.02 – 6.93 (m, 3H, 3 H Ar), 6.84 (t, J = 7.3 Hz, 1H, H Ar), 6.59 (d, J = 7.6 Hz, 1H, H Ar), 6.48 (d, J = 2.0 Hz, 1H, H Ar), 5.89 (d, J = 2.0 Hz, 1H, H Ar), 4.86 (bs, 1H, NH), 4.01 (td, J = 11.3, 3.1 Hz, 1H, 1 H of CH_2 THIQ), 3.77 (s, 3H, OCH_3), 3.60 – 3.54 (m, 1H, 1 H of CH_2 THIQ), 3.54 (s, 3H, OCH_3), 3.21 (ddd, J = 16.4, 11.5, 5.2 Hz, 1H, 1 H of CH_2 THIQ), 3.02 (dt, J = 15.8, 3.0 Hz, 1H, 1 H of CH_2 THIQ), 1.29 (s, 9H, 3 CH_3 of _t_-Bu); ^{13}C NMR (101 MHz, $CDCl_3$) δ 177.7 (Cq amidine), 162.2 (Cq near O), 159.6 (Cq near N), 155.0 (Cq near O), 150.4 (Cq near N), 136.0 (Cq Ar), 134.3 (Cq Ar), 128.7 (CH Ar), 128.3 (2 CH Ar), 126.7 (CH Ar), 126.7 (CH Ar), 126.2 (CH Ar), 122.5 (CH Ar), 121.5 (2 CH Ar), 118.2 (Cq Ar), 95.7 (CH Ar), 92.8 (CH Ar), 77.4 (Cq spiro), 55.5 (OCH_3), 55.1 (OCH_3), 52.0 (Cq _t_-Bu), 46.6 (CH_2 of THIQ), 31.5 (CH_2 of THIQ), 28.5 (3 CH_3 of _t_-Bu); HRMS (ESI^+): m/z calcd for $C_{28}H_{32}N_3O_2$ $[M+H]^+$: 442.2489. Found: 442.2493.

N-(tert-Butyl)-6-methoxy-2'-(4-methoxyphenyl)-3',4'-dihydro-2'H-spiro[indole-3,1'-isoquinolin]-2-amine (3e)

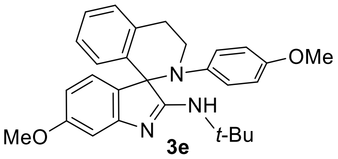

3e

A solution of *N*-(4-methoxyphenyl)tetrahydroisoquinoline (1 equiv, 0.38 mmol, 90 mg), 3-methoxyaniline (1.5 equiv, 0.56 mmol, 63 µL) and $BrCCl_3$ (3 equiv, 1.14 mmol, 112 µL) in CH_3CN (2.0 mL, 0.2 M) in a vial was degassed with argon for 2 minutes, then *t*-butyl isocyanide (2.2 equiv, 0.84 mmol, 94 µL) was added and the vial sealed. The mixture was irradiated at 30 °C at 451 nm (blue LEDs) under magnetic stirring for 24 h. The reaction mixture was treated with Et_3N (3 equiv, 1.14 mmol, 160 µL), concentrated and the residue was purified by column chromatography on silica with PE/AcOEt (from 85:15 + 1% of Et_3N to 80:20 + 1% of Et_3N) to give **3e** (81 mg, 48%) as yellow oil; Rf 0.38 (PE/AcOEt 80:20); I.R.: ῡ (cm^{-1}) = 3407, 2961, 2929, 2833, 2051, 1567, 1507, 1474, 1464, 1439, 1391, 1363, 1341, 1275, 1244, 1220, 1188, 1148, 1120, 1095, 1031, 965, 895, 835, 792, 766, 745, 716, 688, 662, 642, 607; ^1H NMR (400 MHz, $CDCl_3$) δ 7.19 (dd, J = 7.6, 1.0 Hz, 1H, H Ar), 7.14 (td, J = 7.3, 1.3 Hz, 1H, H Ar), 7.05 (d, J = 8.1 Hz, 1H, H Ar), 7.02 – 6.96 (m, 1H, H Ar), 6.88 – 6.82 (m, 2H, 2 H Ar), 6.75 (d, J = 2.4 Hz, 1H, H Ar), 6.65 – 6.57 (m, 3H, 3 H Ar), 6.32 (dd, J = 8.1, 2.4 Hz, 1H, H Ar), 4.81 (bs, 1H, NH), 4.02 – 3.93 (m, 1H, 1 H of CH_2 THIQ), 3.77 (s, 3H, OCH_3), 3.69 (s, 3H, OCH_3), 3.43 – 3.25 (m, 2H, 2 H of CH_2 THIQ), 3.10 – 2.92 (m, 1H, 1 H of CH_2 THIQ), 1.31 (s, 9H, 3 CH_3 of *t*-Bu); ^{13}C NMR (101 MHz, $CDCl_3$) δ 175.1 (Cq amidine), 160.8 (Cq near O), 158.2 (Cq near N), 156.5 (Cq near O), 142.6 (Cq near N), 136.8 (Cq Ar), 133.9 (Cq Ar), 132.5 (Cq Ar), 129.0 (CH Ar), 126.9 (CH Ar), 126.7 (2 CH Ar), 126.5 (2 CH Ar), 123.9 (CH Ar), 113.5 (CH Ar), 106.9 (CH Ar), 103.2 (CH Ar), 76.3 (Cq spiro), 55.4 (2 OCH_3), 52.1 (Cq *t*-Bu), 48.2 (CH_2 of THIQ), 30.7 (CH_2 of THIQ), 28.7 (3 CH_3 of *t*-Bu); HRMS (ESI^+): m/z calcd for $C_{28}H_{32}N_3O_2$ $[M+H]^+$: 442.2489. Found: 442.2490.

***N*-Cyclohexyl-4,6-dimethoxy-2'-phenyl-3',4'-dihydro-2'*H*-spiro[indole-3,1'-isoquinolin]-2-amine (3f)**

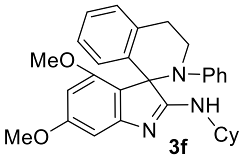

3f

A solution of *N*-phenyltetrahydroisoquinoline (1 equiv, 0.48 mmol, 100 mg), 3,5-dimethoxyaniline (1.5 equiv, 0.72 mmol, 110 mg) and $BrCCl_3$ (3 equiv, 1.44 mmol, 140 µL) in CH_3CN (2.4 mL, 0.2 M) in a vial was degassed with argon for 2 minutes, then cyclohexyl isocyanide (2.2 equiv, 1.06 mmol, 132 µL) was added and the vial sealed. The mixture was irradiated at 30 °C at 451 nm (blue LEDs) under magnetic stirring for 24 h. The reaction mixture was treated with Et_3N (3 equiv, 1.44 mmol, 200 µL), concentrated and the residue was purified by column chromatography on alumina with PE/CH_2Cl_2/Et_2O (from 6:2:2 to 5:3:2) to give **3f** (162 mg, 72%) as cream foam; Rf 0.30 (PE/AcOEt 80:20); I.R.: ῡ (cm^{-1}) = 3400, 2929, 2852, 2191, 1597, 1567, 1515, 1491, 1463, 1449, 1433, 1418, 1370, 1350, 1281, 1239, 1212, 1196, 1142, 1119, 1106, 1036, 1010, 972, 937, 908, 891, 818, 777, 758, 745, 726, 694, 660, 639; ^{1}H NMR (400 MHz, $CDCl_3$) δ 7.17 (d, *J* = 7.5 Hz, 1H, H Ar), 7.12 (t, *J* = 7.3 Hz, 1H, H Ar), 7.06 (t, *J* = 7.7 Hz, 2H, 2 H Ar), 7.00 – 6.92 (m, 3H, 3 H Ar), 6.84 (t, *J* = 7.3 Hz, 1H, H Ar), 6.62 (d, *J* = 8.0 Hz, 2H, 2 H Ar), 6.46 (d, *J* = 2.0 Hz, 1H, H Ar), 5.89 (d, *J* = 2.0 Hz, 1H, H Ar), 4.84 (bd, *J* = 8.6 Hz, 1H, NH), 4.03 (td, *J* = 11.2, 2.0 Hz, 1H, 1 H of CH_2 THIQ), 3.87 – 3.77 (m, 1H, CH of Cy), 3.75 (s, 3H, OCH_3), 3.59 – 3.51 (m, 1H, 1 H of CH_2 THIQ), 3.55 (s, 3H, OCH_3), 3.24 (ddd, *J* = 16.5, 11.9, 5.0 Hz, 1H, 1 H of CH_2 THIQ), 3.01 (dt, *J* = 15.4, 2.1 Hz, 1H, 1 H of CH_2 THIQ), 1.99 – 1.87 (m, 1H, 1 H of CH_2 Cy), 1.80 – 1.71 (m, 1H, 1 H of CH_2 Cy), 1.69 – 1.44 (m, 3H, 3 H of CH_2 Cy), 1.40 – 1.25 (m, 2H, 2 H of CH_2 Cy), 1.19 – 1.04 (m, 2H, 2 H of CH_2 Cy), 0.95 – 0.80 (m, 1H, 1 H of CH_2 Cy); ^{13}C NMR (101 MHz, $CDCl_3$) δ 178.6 (Cq amidine), 162.4 (Cq near O), 159.1 (Cq near N), 155.1 (Cq near O), 150.3 (Cq near N), 135.7 (Cq Ar), 134.4 (Cq Ar), 128.8 (CH Ar), 128.4 (2 CH

Ar), 126.8 (CH Ar), 126.7 (CH Ar), 126.3 (CH Ar), 122.7 (CH Ar), 121.7 (2 CH Ar), 118.6 (Cq Ar), 95.6 (CH Ar), 92.7 (CH Ar), 76.7 (Cq spiro), 55.5 (OCH_3), 55.2 (OCH_3), 50.9 (CH of Cy), 46.6 (CH_2 of THIQ), 32.8 (2 CH_2 of Cy), 31.5 (CH_2 of THIQ), 25.6 (CH_2 of Cy), 24.8 (CH_2 of Cy), 24.6 (CH_2 of Cy); HRMS (ESI^+): m/z calcd for $C_{30}H_{34}N_3O_2$ $[M+H]^+$: 468.2646. Found: 468.2650.

***N*-Butyl-4,6-dimethoxy-2'-phenyl-3',4'-dihydro-2'*H*-spiro[indole-3,1'-isoquinolin]-2-amine 3g**

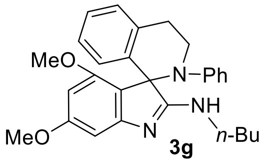

3g

A solution of *N*-phenyl-tetrahydroisoquinoline (1 equiv, 0.48 mmol, 100 mg), 3,5-dimethoxyaniline (1.5 equiv, 0.72 mmol, 110 mg) and $BrCCl_3$ (3 equiv, 1.44 mmol, 140 µL) in CH_3CN (2.4 mL, 0.2 M) in a vial was degassed with argon for 2 minutes, then *n*-butyl isocyanide (2.2 equiv, 1.06 mmol, 111 µL) was added and the vial sealed. The mixture was irradiated at 30 °C at 451 nm (blue LEDs) under magnetic stirring for 24 h. The reaction mixture was treated with Et_3N (3 equiv, 1.44 mmol, 200 µL), concentrated and the residue was purified by column chromatography on alumina with $PE/CH_2Cl_2/Et_2O$ (from 6:2:2 to 5:3:2) to give **3g** (113 mg, 53%) as white foam; Rf 0.15 (PE/AcOEt 80:20); I.R.: ῡ (cm^{-1}) = 3213, 2956, 2931, 2871, 1597, 1571, 1491, 1463, 1449, 1432, 1417, 1370, 1279, 1213, 1195, 1142, 1119, 1105, 1080, 1036, 973, 936, 907, 820, 745, 726, 693, 658, 639, 604; ^1H NMR (400 MHz, $CDCl_3$) δ 7.18 (d, *J* = 6.3 Hz, 1H, H Ar), 7.13 (td, *J* = 7.3, 1.3 Hz, 1H, H Ar), 7.06 (tt, *J* = 7.3, 2.1 Hz, 2H, 2 H Ar), 7.00 – 6.92 (m, 3H, 3 H Ar), 6.84 (tt, *J* = 7.3, 1.2 Hz, 1H, H Ar), 6.62 (d, *J* = 8.1 Hz, 1H, H Ar), 6.46 (d, *J* = 2.0 Hz, 1H, H Ar), 5.90 (d, *J* = 2.1 Hz, 1H, H Ar), 4.98 (bt, *J* = 5.4 Hz, 1H, NH), 4.04 (td, *J* = 11.5, 3.0 Hz, 1H, 1 H of CH_2 THIQ), 3.75 (s, 3H, OCH_3), 3.59 – 3.54 (m, 1H, 1 H of CH_2 THIQ), 3.55 (s, 3H, OCH_3), 3.44 – 3.16 (m, 3H, , 1 H of CH_2 THIQ and CH_2 of *n*-Bu), 3.01 (dt, *J* = 15.8, 2.9 Hz, 1H, 1 H of CH_2 THIQ), 1.44 (td, *J* = 7.2, 3.6 Hz, 2H, CH_2 of *n*-Bu), 1.15 (h, *J* = 7.5 Hz, 2H, CH_2 of *n*-Bu),

0.81 (t, J = 7.4 Hz, 3H, CH_3 of n-Bu); ^{13}C NMR (101 MHz, $CDCl_3$) δ 179.6 (Cq amidine), 162.4 (Cq near O), 158.6 (Cq near N), 155.1 (Cq near O), 150.2 (Cq near N), 135.5 (Cq Ar), 134.5 (Cq Ar), 128.9 (CH Ar), 128.4 (2 CH Ar), 126.9 (CH Ar), 126.8 (CH Ar), 126.4 (CH Ar), 122.8 (CH Ar), 121.6 (2 CH Ar), 118.5 (Cq Ar), 95.7 (CH Ar), 92.9 (CH Ar), 76.6 (Cq spiro), 55.5 (OCH_3), 55.2 (OCH_3), 46.6 (CH_2 of THIQ), 42.6 (CH_2 of n-Bu), 31.5 (CH_2 of THIQ), 31.2 (CH_2 of n-Bu), 20.0 (CH_2 of n-Bu), 13.8 (CH_3 of n-Bu); HRMS (ESI^+): m/z calcd for $C_{28}H_{32}N_3O_2$ $[M+H]^+$: 442.2489. Found: 442.2485.

4,6-Dimethoxy-2'-phenyl-*N*-(2,4,4-trimethylpentan-2-yl)-3',4'-dihydro-2'*H*-spiro[indole-3,1'-isoquinolin]-2-amine (3h)

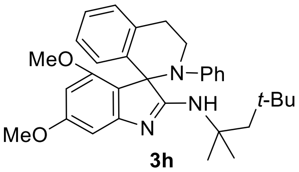

3h

A solution of N-phenyltetrahydroisoquinoline (1 equiv, 0.48 mmol, 100 mg), 3,5-dimethoxyaniline (1.5 equiv, 0.72 mmol, 110 mg) and $BrCCl_3$ (3 equiv, 1.44 mmol, 140 µL) in CH_3CN (2.4 mL, 0.2 M) in a vial was degassed with argon for 2 minutes, then 1,1,3,3-tetramethylbutyl isocyanide (Walborsky's reagent) (2.2 equiv, 1.06 mmol, 185 µL) was added and the vial sealed. The mixture was irradiated at 30 °C at 451 nm (blue LEDs) under magnetic stirring for 24 h. The reaction mixture was treated with Et_3N (3 equiv, 1.44 mmol, 200 µL), concentrated and the residue was purified by column chromatography on silica gel with PE/AcOEt (from 10:1 + 1% Et_3N to 9:1 + 1% Et_3N) to give **3h** (81 mg, 34%) as white foam; Rf 0.56 (PE/AcOEt 80:20); I.R.: ῡ (cm^{-1}) = 3409, 2949, 2092, 1598, 1572, 1516, 1490, 1451, 1434, 1418, 1383, 1365, 1324, 1275, 1225, 1208, 1194, 1144, 1118, 1107, 1046, 1031, 992, 973, 940, 820, 802, 765, 748, 724, 696, 662, 640; ^1H NMR (400 MHz, $CDCl_3$) δ 7.13 (dd, J = 7.8, 1.8 Hz, 1H, H Ar), 7.12 – 7.03 (m, 3H, 3 H Ar), 7.00 – 6.93 (m, 3H, 3 H Ar), 6.82 (tt, J = 7.1, 1.2 Hz, 1H, H Ar), 6.59 (dd, J = 8.0, 1.2 Hz, 1H, H Ar), 6.48 (d, J = 2.0 Hz, 1H, H Ar), 5.88 (d, J = 2.1 Hz, 1H, H Ar), 5.08 (bs, 1H, NH), 4.00 (td, J = 11.6, 3.0 Hz,

1H, 1 H of CH_2 THIQ), 3.77 (s, 3H, OCH_3), 3.61 (ddd, *J* = 11.1, 5.1, 2.4 Hz, 1H, 1 H of CH_2 THIQ), 3.54 (s, 3H, OCH_3), 3.19 (ddd, *J* = 15.7, 11.8, 5.1 Hz, 1H, 1 H of CH_2 THIQ), 2.99 (dt, *J* = 15.7, 2.8 Hz, 1H, 1 H of CH_2 THIQ), 1.61 (d, *J* = 15.1 Hz, 1H, 1 H of CH_2), 1.42 (d, *J* = 15.1 Hz, 1H, 1 H of CH_2), 1.42 (s, 3H, CH_3), 1.41 (s, 3H, CH_3), 0.76 (s, 9H, 3 CH_3 of *t*-Bu); ^{13}C NMR (101 MHz, $CDCl_3$) δ 177.5 (Cq near amidine), 162.2 (Cq near OCH_3), 159.9 (Cq near N), 155.0 (Cq near OCH_3), 150.4 (Cq), 135.8 (Cq), 134.4 (Cq), 128.8 (CH Ar), 128.3 (2 CH Ar), 126.72 (CH Ar), 126.69 (CH Ar), 126.4 (CH Ar), 122.2 (CH Ar), 121.1 (2 CH Ar), 117.9 (Cq), 95.6 (CH Ar), 92.6 (CH Ar), 77.6 (Cq spiro), 56.2 (Cq), 55.5 (OCH_3), 55.1 (OCH_3), 54.9 (CH_2), 46.4 (CH_2 of THIQ), 31.7 (CH_2 of THIQ), 31.6 (Cq), 31.4 (3 CH_3 of *t*-Bu), 28.6 (CH_3), 26.7 (CH_3); HRMS (ESI^+): m/z calcd for $C_{32}H_{40}N_3O_2$ $[M+H]^+$: 498.3115. Found: 498.3118.

***N*-(*tert*-Butyl)-4,6-dimethoxy-2'-(4-(trifluoromethyl)phenyl)-3',4'-dihydro-2'*H*-spiro[indole-3,1'-isoquinolin]-2-amine (3i)**

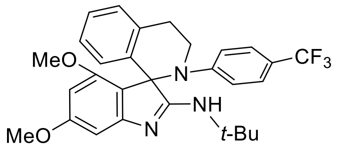

3i

A solution of 2-(4-(trifluoromethyl)phenyl)-1,2,3,4-tetrahydroisoquinoline (1 equiv, 0.31 mmol, 86 mg), 3,5-dimethoxyaniline (1.5 equiv, 0.47 mmol, 71 mg) and $BrCCl_3$ (3 equiv, 0.93 mmol, 91 µL) in CH_3CN (1.6 mL, 0.2 M) in a vial was degassed with argon for 2 minutes, then *t*-butyl isocyanide (2.2 equiv, 0.68 mmol, 78 µL) was added and the vial sealed. The mixture was irradiated at 30 °C at 451 nm (blue LEDs) under magnetic stirring for 24 h. The reaction mixture was treated with Et_3N (3 equiv, 0.93 mmol, 129 µL), concentrated and the residue was purified by column chromatography on silica gel with PE/AcOEt (from 10:1 + 1% Et_3N to 85:15 + 1% Et_3N) to give **2i** (25 mg, 16%) as brown oil and **3i** (66 mg, 41%) as yellow oil; characterization of **2i**: Rf 0.52 (PE/AcOEt 85:15 + 1% Et_3N); I.R.: ῡ (cm^{-1}) = 3416, 2959, 1640, 1586, 1511, 1454, 1422, 1388, 1358, 1323, 1201, 1147, 1109, 1069, 1008, 938, 821, 752, 691, 670, 631; ^{1}H NMR (400 MHz, $CDCl_3$) δ 7.46 (d, *J* = 8.1 Hz, 2H, 2 H Ar), 7.20

(td, *J* = 7.4, 1.4 Hz, 1H, 1 H Ar), 7.16 – 7.08 (m, 2H, 2 H Ar), 6.93 (d, *J* = 7.7 Hz, 2H, 2 H Ar), 6.88 (d, *J* = 8.6 Hz, 2H, 2 H Ar), 6.04 (t, *J* = 2.3 Hz, 1H, H Ar), 5.88 (d, *J* = 2.3 Hz, 2H, 2 H Ar), 5.57 (s, 1H, CH), 4.63 (s, 1H, NH), 3.64 (s, 6H, 2 OCH_3), 3.63 – 3.58 (m, 1H, 1 H of CH_2 THIQ), 3.48 (ddt, *J* = 12.0, 8.0, 3.7 Hz, 1H, 1 H of CH_2 THIQ), 3.07 (ddd, *J* = 15.7, 7.9, 4.6 Hz, 1H, 1 H of CH_2 THIQ), 2.90 (ddd, *J* = 15.7, 6.6, 4.6 Hz, 1H, 1 H of CH_2 THIQ), 1.29 (s, 9H, 3 CH_3 of *t*-Bu); ^{13}C NMR – ^{19}F dec (101 MHz, $CDCl_3$) δ 161.3 (Cq of amidine), 153.7 (2 Cq near O), 152.4 (Cq near N), 152.0 (Cq near N), 135.7 (Cq Ar), 133.9 (Cq Ar), 128.7 (CH Ar), 128.2 (CH Ar), 127.7 (CH Ar), 126.4 (CH Ar), 126.3 (2 CH Ar of *p*-CF_3-Ph), 124.8 (CF_3), 121.4 (Cq near CF_3), 115.9 (2 CH Ar of *p*-CF_3-Ph), 100.1 (2 CH Ar), 94.3 (CH Ar), 59.3 (CH), 55.3 (2 OCH_3), 51.4 (Cq of *t*-Bu), 45.9 (CH_2 of THIQ), 28.8 (CH_2 of THIQ), 28.5 (3 CH_3 of *t*-Bu)); ^{19}F NMR (376 MHz, $CDCl_3$) δ -61.27 (s, 3 F); HRMS (ESI$^+$): m/z calcd for $C_{29}H_{33}F_3N_3O_2$ $[M+H]^+$: 512.2519. Found: 512.2525. Characterization of **3i:** Rf 0.36 (PE/AcOEt 85:15 + 1% Et_3N); I.R.: ῡ (cm^{-1}) = 3406, 2964, 1600, 1572, 1520, 1454, 1418, 1364, 1323, 1210, 1146, 1107, 1071, 1009, 972, 942, 823, 764, 745, 689, 670, 637; ^1H NMR (400 MHz, $CDCl_3$) δ 7.31 (d, *J* = 8.1 Hz, 2H, 2 H Ar), 7.18 (dd, *J* = 7.7, 1.8 Hz, 1H, H Ar), 7.15 (td, *J* = 7.2, 1.2 Hz, 1H, H Ar), 7.00 (td, *J* = 7.6, 6.9, 1.9 Hz, 1H, H Ar), 6.97 – 6.94 (m, 2H, 2 H Ar), 6.62 (dd, *J* = 8.1, 1.2 Hz, 1H, H Ar), 6.55 (d, *J* = 2.0 Hz, 1H, H Ar), 5.90 (d, *J* = 2.0 Hz, 1H, H Ar), 4.71 (bs, 1H, NH), 3.93 (td, *J* = 11.1, 3.4 Hz, 1H, 1 H of CH_2 THIQ), 3.80 (s, 3H, OCH_3), 3.73 (ddd, *J* = 11.3, 4.8, 3.2 Hz, 1H, 1 H of CH_2 THIQ), 3.51 (s, 3H, OCH_3), 3.18 (ddd, *J* = 15.8, 10.9, 4.8 Hz, 1H, 1 H of CH_2 THIQ), 3.08 (dt, *J* = 15.8, 3.4 Hz, 1H, 1 H of CH_2 THIQ), 1.26 (s, 9H, 3 CH_3 of *t*-Bu); ^{13}C NMR – ^{19}F dec (101 MHz, $CDCl_3$) δ 177.2 (Cq amidine), 162.6 (Cq near O), 159.6 (Cq near N), 154.9 (Cq near O), 153.1 (Cq of N), 135.6 (Cq), 134.1 (Cq), 128.7 (CH Ar), 127.03 (CH Ar), 126.98 (CH Ar), 126.0 (CH Ar), 125.6 (2 CH Ar of *p*-CF_3-Ph), 124.7 (CF_3), 122.7 (Cq near CF_3), 119.1 (2 CH Ar), 117.3 (Cq), 96.1 (CH Ar), 93.0 (CH Ar), 77.4 (Cq spiro), 55.6 (OCH_3), 55.2 (OCH_3), 52.2 (Cq of *t*-Bu), 45.9 (CH_2 of THIQ), 31.2 (CH_2 of THIQ), 28.5 (3 CH_3 of *t*-Bu); ^{19}F NMR (376 MHz, $CDCl_3$) δ -

61.53 (s, 3 F); HRMS (ESI+): m/z calcd for $C_{29}H_{31}F_3N_3O_2$ $[M+H]^+$: 510.2363. Found: 510.2372.

***N*-(*tert*-Butyl)-6,6',7'-trimethoxy-2'-phenyl-3',4'-dihydro-2'*H*-spiro[indole-3,1'-isoquinolin]-2-amine (3j)**

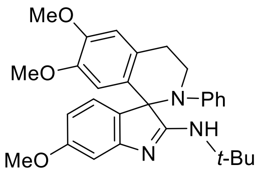

3j

A solution of 6,7-dimethoxy-2-phenyl-1,2,3,4-tetrahydroisoquinoline (1 equiv, 0.36 mmol, 96 mg), 3-methoxyaniline (1.5 equiv, 0.54 mmol, 60 µL) and $BrCCl_3$ (3 equiv, 1.80 mmol, 106 µL) in CH_3CN (1.8 mL, 0.2 M) in a vial was degassed with argon for 2 minutes, then *tert*-butyl isocyanide (2.2 equiv, 0.79 mmol, 90 µL) was added and the vial sealed. The mixture was irradiated at 30 °C using white LEDs under magnetic stirring for 24 h. The reaction mixture was treated with Et_3N (3 equiv, 1.09 mmol, 149 µL), concentrated and the residue was purified by column chromatography on silica gel with PE/AcOEt (75:25 + 1% Et_3N) to give a mixture of **3j** and formamidine **S8** (86 mg, ratio 35:65, yield of **3j** 41%). An analytical sample of **3j** was obtained by preparative TLC (PE/AcOEt 80:20 + 1% Et_3N): I.R.: ῡ (cm^{-1}) = 3405, 2958, 2931, 2834, 1568, 1513, 1463, 1440, 1404, 1364, 1334, 1253, 1212, 1148, 1124, 1030, 980, 954, 911, 857, 808, 782, 754, 696, 666, 642; Rf 0.29 (PE/AcOEt 75: 25 + 1% Et_3N); ^{1}H NMR (400 MHz, $CDCl_3$) δ 7.12 – 7.05 (m, 2H, 2 CH Ar of Ph), 7.04 (d, *J* = 8.0 Hz, 1H, CH Ar), 6.94 – 6.84 (m, 3H, 3 CH Ar of Ph), 6.79 (d, *J* = 2.3 Hz, 1H, CH Ar), 6.64 (s, 1H, CH Ar), 6.32 (dd, *J* = 8.1, 2.4 Hz, 1H, CH Ar), 6.11 (s, 1H, CH Ar), 4.73 (s, 1H, NH), 3.98 (ddd, *J* = 11.9, 10.8, 3.6 Hz, 1H, 1 H of CH_2 THIQ), 3.87 (s, 3H, OCH_3), 3.77 (s, 3H, OCH_3), 3.58 (s, 3H, OCH_3), 3.57 – 3.50 (m, 1H, 1 H of CH_2 THIQ), 3.24 (ddd, *J* = 16.0, 10.7, 5.4 Hz, 1H, 1 H of CH_2 THIQ), 2.94 (dt, *J* = 15.8, 3.3 Hz, 1H, 1 H of CH_2 THIQ), 1.28 (s, 9H, 3 CH_3 of *t*-Bu); ^{13}C NMR (101 MHz, $CDCl_3$) δ 175.2 (Cq of amidine), 160.8 (Cq near O),

157.8 (Cq near N), 149.7 (Cq near N), 148.1 (Cq near O), 148.0 (Cq near O), 132.2 (Cq Ar), 128.6 (Cq Ar), 128.5 (2 CH Ar), 126.3 (Cq Ar), 123.4 (4 CH Ar), 110.9 (CH Ar), 108.7 (CH Ar), 107.0 (CH Ar), 103.5 (CH Ar), 75.7 (Cq of spiro), 55.9 (OCH_3), 55.8 (OCH_3), 55.4 (OCH_3), 52.1 (Cq of *t*-Bu), 47.9 (CH_2), 30.4 (CH_2), 28.6 (3 CH_3 of *t*-Bu).

Ethyl (4,6-dimethoxy-2'-phenyl-3',4'-dihydro-2'*H*-spiro[indole-3,1'-isoquinolin]-2-yl)glycinate (3k)

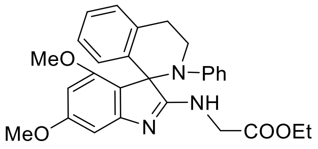

3k

A solution of *N*-phenyl-tetrahydroisoquinoline (1 equiv, 0.48 mmol, 100 mg), 3,5-dimethoxyaniline (1.5 equiv, 0.72 mmol, 110 mg) and $BrCCl_3$ (3 equiv, 1.44 mmol, 140 µL) in CH_3CN (2.4 mL, 0.2 M) in a vial was degassed with argon for 2 minutes, then ethyl isocyanoacetate (2.2 equiv, 1.06 mmol, 115 µL) was added and the vial sealed. The mixture was irradiated at 30 °C at 451 nm (blue LEDs) under magnetic stirring for 24 h. The reaction mixture was treated with Et_3N (3 equiv, 1.44 mmol, 200 µL), concentrated and the residue was purified by column chromatography on silica gel with PE/AcOEt (from 80:20 + 1% Et_3N to 60:40 + 1% Et_3N) to give **3k** (105 mg, 46%) as yellow oil; Rf 0.33 (PE/AcOEt 60:40); I.R.: ῡ (cm^{-1}) = 2925, 2850, 1737, 1576, 1491, 1450, 1418, 1374, 1280, 1254, 1195, 1145, 1103, 1027, 973, 937, 820, 764, 747, 696, 636; ^{1}H NMR (400 MHz, $CDCl_3$) δ 7.20 (d, *J* = 6.8 Hz, 1H, H Ar), 7.15 (td, *J* = 7.3, 1.3 Hz, 1H, H Ar), 7.09 – 7.02 (m, 2H, 2 H Ar), 7.01 – 6.94 (m, 3H, 3 H Ar), 6.85 (t, *J* = 7.2 Hz, 1H. H Ar), 6.58 (dd, *J* = 8.1, 1.3 Hz, 1H, H Ar), 6.42 (d, *J* = 2.0 Hz, 1H, H Ar), 5.93 (d, *J* = 2.0 Hz, 1H, H Ar), 5.67 (bs, 1H, NH), 4.23 (bd, *J* = 16.5 Hz, 1H, 1 H of CH_2-COOEt), 4.19 (q, *J* = 7.1 Hz, 2H, CH_2 of COOEt), 4.07 (d, *J* = 16.5 Hz, 1H, 1 H of CH_2-COOEt), 4.00 (td, *J* = 11.4, 2.9 Hz, 1H, 1 H of CH_2 THIQ), 3.75 (s, 3H, OCH_3), 3.57 (s, 3H, OCH_3), 3.53 (ddd, *J* = 11.0, 5.1, 2.6 Hz, 1H, 1 H of CH_2 THIQ), 3.31 (ddd, *J* =

16.4, 11.6, 5.0 Hz, 1H, 1 H of CH_2 THIQ), 3.01 (dt, J = 15.9, 2.8 Hz, 1H, 1 H of CH_2 THIQ), 1.25 (t, J = 7.1 Hz, 3H, CH_3 of COOEt); ^{13}C NMR (101 MHz, $CDCl_3$) δ 179.3 (C=O of COOEt), 169.8 (Cq amidine), 162.4 (Cq near O), 155.2 (Cq near O), 150.1 (Cq near N), 134.9 (2 Cq Ar), 129.0 (CH Ar), 128.4 (2 CH Ar), 127.0 (CH Ar), 126.7 (CH Ar), 126.3 (CH Ar), 123.2 (CH Ar), 122.3 (2 CH Ar), 95.9 (CH Ar), 93.3 (CH Ar), 77.4 (Cq spiro), 61.7 (CH_2 of Et), 55.5 (OCH_3), 55.2 (OCH_3), 46.7 (CH_2 of THIQ), 44.2 (CH$_2$COOEt), 31.5 (CH_2 of THIQ), 14.3 (CH_3 of Et); HRMS (ESI$^+$): m/z calcd for $C_{28}H_{30}N_3O_4$ [M+H]$^+$: 472.2231. Found: 472.2240.

***N*-(*tert*-Butyl)-6-methoxy-2'-(4-nitrophenyl)-3',4'-dihydro-2'*H*-spiro[indole-3,1'-isoquinolin]-2-amine (3m)**

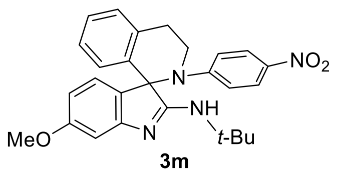

3m

A solution of 2-(4-nitrophenyl)-1,2,3,4-tetrahydroisoquinoline (1 equiv, 0.38 mmol, 97 mg), 3-methoxyaniline (1.5 equiv, 0.57 mmol, 64 µL) and $BrCCl_3$ (3 equiv, 1.14 mmol, 112 µL) in CH_3CN (2 mL, 0.2 M) in a vial was degassed with argon for 2 minutes, then *t*-butyl isocyanide (2.2 equiv, 0.84 mmol, 84 µL) was added and the vial sealed. The mixture was irradiated at 30 °C at 451 nm (blue LEDs) under magnetic stirring for 24 h. The reaction mixture was treated with Et_3N (3 equiv, 1.14 mmol, 160 µL), concentrated and the residue was purified by column chromatography on silica gel with PE/AcOEt (from 90:10 + 1% Et_3N to 75:25 + 1% Et_3N) to give **2m** (49 mg, 28%) as yellow oil and **3m** (21 mg, 12%) as yellow oil; characterization of **2m**: Rf 0.76 (PE/AcOEt 70:30 + 1% Et_3N); I.R.: ῡ (cm^{-1}) = 3398, 2963, 2920, 2117, 1635, 1586, 1481, 1390, 1361, 1311, 1301, 1258, 1234, 1203, 1145, 1110, 1039, 983, 960, 935, 854; ^{1}H NMR (400 MHz, $CDCl_3$) δ 8.04 (d, J = 8.9 Hz, 2H, 2 H Ar), 7.24 (d, J = 7.3 Hz, 1H, H Ar), 7.21 – 7.12 (m, 2H, 2 H Ar), 7.08 (t, J = 8.0 Hz, 1H, H Ar), 6.92 (d, J = 7.6 Hz, 1H, H Ar), 6.61 (d, J = 9.0 Hz, 2H, 2 H Ar), 6.44 (d, J = 8.2 Hz, 1H, H

Ar), 6.36 (d, J = 7.8 Hz, 1H, H Ar), 6.22 (t, J = 2.2 Hz, 1H, H Ar), 5.74 (s, 1H, CH of THIQ), 4.30 (bs, 1H, NH), 3.72 – 3.66 (m, 2H, CH_2 of THIQ), 3.64 (s, 3H, OCH_3), 3.07 – 2.87 (m, 2H, CH_2 of THIQ), 1.30 (s, 9H, 3 CH_3 of *t*-Bu); ^{13}C NMR (101 MHz, $CDCl_3$) δ 160.6 (Cq near O), 153.3 (Cq near N), 152.3 (Cq of amidine), 151.6 (Cq near N), 138.8 (Cq near N), 135.5 (Cq), 133.0 (Cq), 129.9 (CH Ar), 128.6 (CH Ar), 128.5 (CH Ar), 128.2 (CH Ar), 126.7 (CH Ar), 125.7 (2 CH Ar), 113.8 (CH Ar), 113.1 (2 CH Ar), 107.4 (2 CH Ar), 58.5 (CH of THIQ), 55.2 (OCH_3), 51.7 (Cq of *t*-Bu), 44.4 (CH_2 of THIQ), 28.5 (3 CH_3 of *t*-Bu), 28.2 (CH_2 of THIQ).; HRMS (ESI^+): m/z calcd for $C_{27}H_{31}N_4O_3$ $[M+H]^+$: 459.2391. Found: 459.2402.

Characterization of **3m**: Rf 0.29 (PE/AcOEt 85:15); I.R.: ῡ (cm^{-1}) = 3384, 2964, 1590, 1567, 1494, 1439, 1391, 1364, 1315, 1299, 1212, 1189, 1149, 1112, 1028, 971, 908, 837, 799, 748, 727, 691, 640; ^1H NMR (400 MHz, $CDCl_3$) δ 8.03 – 7.92 (m, 2H, 2 H Ar), 7.22 (dd, J = 7.6, 1.7 Hz, 1H, H Ar), 7.18 (td, J = 7.3, 1.2 Hz, 1H, H Ar), 7.06 (td, J = 7.7, 7.1, 1.7 Hz, 1H, H Ar), 6.94 (d, J = 2.4 Hz, 1H, H Ar), 6.90 (d, J = 8.2 Hz, 1H, H Ar), 6.82 – 6.73 (m, 3H, 3 H Ar), 6.34 (dd, J = 8.1, 2.4 Hz, 1H, H Ar), 4.43 (bs, 1H, NH), 4.04 – 3.88 (m, 2H, CH_2 of THIQ), 3.81 (s, 3H), 3.33 – 3.18 (m, 2H, CH_2 of THIQ), 1.24 (s, 9H, 3 CH_3 of *t*-Bu); ^{13}C NMR (101 MHz, $CDCl_3$) δ 174.1 (Cq of amidine), 161.4 (Cq near O), 157.5 (Cq near N), 154.6 (Cq near N), 140.4 (Cq near N), 136.1 (Cq), 133.5 (Cq), 131.5 (Cq), 128.8 (CH Ar), 127.7 (CH Ar), 127.5 (CH Ar), 125.9 (CH Ar), 125.0 (2 CH Ar), 122.3 (CH Ar), 117.5 (2 CH Ar), 108.0 (CH Ar), 104.3 (CH Ar), 75.5 (Cq spiro), 55.5 (OCH_3), 52.6 (Cq of *t*-Bu), 47.2 (CH_2 of THIQ), 30.8 (CH_2 of THIQ), 28.4 (3 CH_3 of *t*-Bu); HRMS (ESI^+): m/z calcd for $C_{27}H_{29}N_4O_3$ $[M+H]^+$: 457.2234. Found: 457.2235.

N-(tert-Butyl)-6-methoxy-2'-(4-(trifluoromethyl)phenyl)-3',4'-dihydro-2'H-spiro[indole-3,1'-isoquinolin]-2-amine (3n)

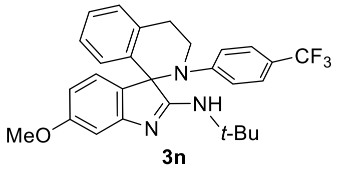

3n

A solution of 2-(4-(trifluoromethyl)phenyl)-1,2,3,4-tetrahydroisoquinoline (1 equiv, 0.32 mmol, 90 mg), 3-methoxyaniline (1.5 equiv, 0.49 mmol, 68 µL) and $BrCCl_3$ (3 equiv, 0.96 mmol, 94 µL) in CH_3CN (1.6 mL, 0.2 M) in a vial was degassed with argon for 2 minutes, then *t*-butyl isocyanide (2.2 equiv, 0.70 mmol, 80 µL) was added and the vial sealed. The mixture was irradiated at 30 °C at 451 nm (blue LEDs) under magnetic stirring for 24 h. The reaction mixture was treated with Et_3N (3 equiv, 0.96 mmol, 134 µL), concentrated and the residue was purified by column chromatography on silica gel with PE/AcOEt 90:10 + 1% Et_3N to give **2n** (31 mg, 20%) as yellow oil and a mixture of **3n** and amino amide derivative **4n** (25 mg, ratio 64:36, respectively 11% and 6%) as yellow oil; characterization of **2n:** Rf 0.50 (PE/AcOEt 85:15 + 1% Et_3N); I.R.: ῡ (cm^{-1}) = 2961, 1639, 1613, 1591, 1521, 1478, 1388, 1362, 1322, 1281, 1257, 1222, 1202, 1161, 1143, 1109, 1071, 937, 821, 747, 695, 668, 632; ^1H NMR (400 MHz, $CDCl_3$) δ 7.46 (d, *J* = 8.7 Hz, 2H, 2 H Ar), 7.20 (td, *J* = 7.4, 1.3 Hz, 1H, H Ar), 7.14 (d, *J* = 6.1 Hz, 1H, H Ar), 7.12 (d, *J* = 1.5 Hz, 1H, H Ar), 7.09 (t, *J* = 8.0 Hz, 1H, H Ar), 6.88 – 6.83 (m, 3H, 3 H Ar), 6.47 (ddd, *J* = 8.2, 2.6, 0.9 Hz, 1H, H Ar), 6.36 (ddd, *J* = 7.8, 1.9, 0.9 Hz, 1H, H Ar), 6.22 (t, *J* = 2.2 Hz, 1H, H Ar), 5.54 (s, 1H, CH near amidine), 4.65 (bs, 1H, NH), 3.65 (s, 3H, OCH_3), 3.65 – 3.58 (m, 1H, 1 H of CH_2 THIQ), 3.46 (ddd, *J* = 12.0, 8.1, 4.5 Hz, 1H, 1 H of CH_2 THIQ), 3.09 (ddd, *J* = 15.7, 8.2, 4.7 Hz, 1H, 1 H of CH_2 THIQ), 2.89 (ddd, *J* = 15.7, 6.5, 4.5 Hz, 1H, 1 H of CH_2 THIQ), 1.29 (s, 9H, 3 CH_3 of *t*-Bu); ^{13}C NMR (101 MHz, $CDCl_3$) δ 160.5 (Cq near O), 153.6 (Cq amidine), 152.0 (Cq near N), 151.8 (Cq near N), 135.6 (Cq Ar), 133.9 (Cq Ar), 129.7 (CH Ar), 128.6 (CH Ar), 128.2

(CH Ar), 127.7 (CH Ar), 126.4 (CH Ar), 126.3 (2 CH Ar of *p*-CF_3-Ph), 124.8 (CF_3), 121.5 (Cq near CF_3), 116.0 (3 CH Ar), 114.2 (CH Ar), 107.53 (CH Ar), 107.50 (CH Ar), 59.3 (CH of THIQ), 55.2 (OCH_3), 51.4 (Cq of *t*-Bu), 46.0 (CH_2 of THIQ), 28.8 (CH_2 of THIQ), 28.5 (3 CH_3 of *t*-Bu); ^{19}F NMR (376 MHz, $CDCl_3$) δ -61.27 (s, 3 F); HRMS (ESI^+): m/z calcd for $C_{28}H_{31}F_3N_3O$ $[M+H]^+$: 482.2414. Found: 482.2419. Characterization of the mixture of **3n** and **4n**: Rf 0.22 (PE/AcOEt 85:15 + 1% Et_3N); ^{1}H NMR (400 MHz, $CDCl_3$) ratio **3n**:**4n** = 64:36 δ-7.57 – 7.50 (m, 3H, 3 H Ar of **4n**), 7.33 (d, *J* = 8.3 Hz, 2H, 2 H Ar of **3n**), 7.31 – 7.23 (m, 2H, 2 H Ar of **4n**), 7.22 – 7.17 (m, 2H, 1 H Ar of **3n** and 1 H Ar of **4n**), 7.18 (td, *J* = 7.6, 1.3 Hz, 1H, 1 H Ar of **3n**), 7.03 (td, *J* = 7.6, 1.7 Hz, 1H, 1 H Ar of **3n**), 6.95 (d, *J* = 8.1 Hz, 1H, 1 H Ar of **3n**), 6.93 (d, *J* = 9.3 Hz, 2H, 2 H Ar of **4n**), 6.88 (d, *J* = 2.4 Hz, 1H, 1 H Ar of **3n**), 6.87 (d, *J* = 8.4 Hz, 2H, 2 H Ar of **3n**), 6.71 (dd, *J* = 8.1, 1.3 Hz, 1H, 1 H Ar of **3n**), 6.42 (s, 1H, NH of **4n**), 6.33 (dd, *J* = 8.1, 2.4 Hz, 1H, 1 H Ar of **3n**), 4.91 (s, 1H, CH of **4n**), 4.56 (s, 1H, NH of **3n**), 3.95 (ddd, *J* = 11.9, 9.6, 3.7 Hz, 1H, 1 H of CH_2 THIQ of **3n**), 3.87 (dt, *J* = 11.0, 4.5 Hz, 1H, 1 H of CH_2 THIQ of **4n**), 3.79 (s, 3H, OCH_3 of **3n**), 3.75 (dt, *J* = 11.9, 4.8 Hz, 1H, 1 H of CH_2 THIQ of **3n**), 3.37 (td, *J* = 10.7, 4.4 Hz, 1H, 1 H of CH_2 THIQ of **4n**), 3.27 (ddd, *J* = 14.9, 9.6, 4.9 Hz, 1H, 1 H of CH_2 THIQ of **3n**), 3.12 (dt, *J* = 16.1, 4.2 Hz, 1H, 1 H of CH_2 THIQ of **3n**), 3.08 – 2.98 (m, 2H, CH_2 THIQ of **4n**), 1.26 (s, 9H, 3 CH_3 of *t*-Bu of **4n**), 1.23 (s, 9H, 3 CH_3 of *t*-Bu of **3n**); ^{13}C NMR (101 MHz, $CDCl_3$) δ 174.8 (Cq amidine of **3n**), 170.7 (Cq amide of **4n**), 161.2 (Cq near O of **3n**), 157.8 (Cq near N of **3n**), 152.4 (Cq near N of **3n**), 151.7 (Cq near N of **4n**), 136.4 (Cq Ar of **3n**), 134.3 (Cq Ar of **4n**), 133.7 (Cq Ar of **3n**), 132.7 (Cq Ar of **4n**), 132.1 (Cq Ar of **3n**), 129.0 (CH Ar of **4n**), 128.9 (CH Ar of **3n**), 127.9 (CH Ar of **4n**), 127.7 (CH Ar of **4n**), 127.3 (CH Ar of **3n**), 127.2 (CH Ar of **4n**), 127.1 (CH Ar of **3n**), 126.7 (2 CH Ar of *p*-CF_3-Ph of **4n**), 126.3 (CH Ar of **3n**), 125.8 (2 CH 2 CH Ar of *p*-CF_3-Ph of **3n**), 124.8 (CF_3 of **4n**), 124.6 (CF_3 of **3n**), 123.4 (Cq near CF_3 of **4n**), 122.9 (CH Ar of **3n**), 121.0 (Cq near CF_3 of **3n**), 120.3 (2 CH 2 CH Ar of *p*-CF_3-Ph of **3n**), 113.5 (2 CH Ar of *p*-CF_3-Ph of **4n**), 107.6 (CH Ar of **3n**), 104.0 (CH Ar of **3n**), 75.6 (Cq spiro of **3n**), 66.0

(CH of **4n**), 55.5 (OCH_3 of **3n**), 52.3 (Cq of *t*-Bu of **4n**), 51.4 (Cq of *t*-Bu of **3n**), 47.4 (CH_2 of THIQ of **3n**), 44.7 (CH_2 of THIQ of **4n**), 30.9 (CH_2 of THIQ of **3n**), 28.8 (CH_2 of THIQ of **4n**), 28.7 (3 CH_3 of *t*-Bu of **4n**), 28.5 (3 CH_3 of *t*-Bu of **4n**); ^{19}F NMR (376 MHz, $CDCl_3$) δ -61.22 (s, 3 F of **3n**), -61.68 (s, 3 F of **4n**); HPLC-MS (ESI^+)-UV (A=H_2O+0,1%FA, B=CH_3CN+0,1%FA,0 min A=90%, 20 min A=0, flow 0.38 mL/min, and 30 °C): Rt: 16.35 min, 480.6 $[M + H]^+$, 20.01 min, 377.4 $[M + H]^+$.

N-(*tert*-Butyl)-4,6-dimethoxy-2'-(4-methoxyphenyl)-3',4'-dihydro-2'*H*-spiro[indole-3,1'-isoquinolin]-2-amine (3o)

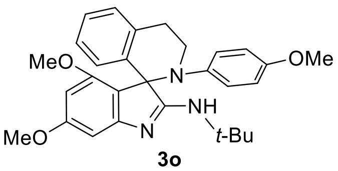

3o

A solution of 2-(4-methoxypheny)-1,2,3,4-tetrahydroisoquinoline (1 equiv, 0.42 mmol, 100 mg), 3,5-dimethoxyaniline (1.5 equiv, 0.63 mmol, 96 mg) and $BrCCl_3$ (3 equiv, 1.26 mmol, 124 µL) in CH_3CN (2.1 mL, 0.2 M) in a vial was degassed with argon for 2 minutes, then *t*-butyl isocyanide (2.2 equiv, 0.92 mmol, 105 µL) was added and the vial sealed. The mixture was irradiated at 30 °C at 451 nm (blue LEDs) under magnetic stirring for 24 h. The reaction mixture was treated with Et_3N (3 equiv, 1.26 mmol, 174 µL), concentrated and the residue was purified by column chromatography on silica gel with PE/AcOEt 80:20 + 1% Et_3N to give **3o** (77 mg, 39%) as white foam. Rf 0.36 (PE/AcOEt 80:20); I.R.: ῡ (cm^{-1}) = 3397, 2959, 2834, 1642, 1573, 1509, 1454, 1417, 1364, 1323, 1268, 1243, 1210, 1144, 1120, 1092, 1065, 1034, 968, 941, 821, 748, 639; ^1H NMR (400 MHz, $CDCl_3$) δ 7.16 (d, *J* = 7.2 Hz, 1H. H Ar), 7.12 (td, *J* = 7.3, 1.2 Hz, 1H, H Ar), 7.02 – 6.91 (m, 3H, 3 H Ar), 6.61 (d, *J* = 9.0 Hz, 2H, 2 H Ar), 6.55 (dd, *J* = 8.0, 1.3 Hz, 1H, H Ar), 6.44 (bs, 1H, H Ar), 5.91 (d, *J* = 2.0 Hz, 1H, H Ar), 4.92 (bs, 1H, NH), 4.07 (td, *J* = 11.4, 3.2 Hz, 1H, 1 H of CH_2 THIQ), 3.76 (s, 3H,

OCH_3), 3.68 (s, 3H, OCH_3), 3.58 (s, 3H, OCH_3), 3.33 (ddd, J = 10.9, 5.5, 2.1 Hz, 1H, 1 H of CH_2 THIQ), 3.23 (ddd, J = 16.7, 11.6, 5.5 Hz, 1H, 1 H of CH_2 THIQ), 2.96 (dt, J = 15.8, 2.7 Hz, 1H, 1 H of CH_2 THIQ), 1.32 (s, 9H, 3 CH_3 of *t*-Bu); ^{13}C NMR (101 MHz, $CDCl_3$) δ 177.3 (Cq amidine), 162.2 (Cq near O), 159.6 (Cq near N), 155.9 (Cq near O), 155.2 (Cq near O), 143.5 (Cq near N), 135.9 (Cq Ar), 134.3 (Cq Ar), 128.8 (CH Ar), 126.6 (CH Ar), 126.5 (CH Ar), 126.3 (CH Ar), 124.6 (2 CH Ar), 118.3 (Cq Ar), 113.5 (2 CH Ar), 95.6 (CH Ar), 92.8 (CH Ar), 78.0 (Cq spiro), 55.5 (OCH_3), 55.3 (OCH_3), 55.2 (OCH_3), 52.0 (Cq of *t*-Bu), 47.4 (CH_2 of THIQ), 31.4 (CH_2 of THIQ), 28.7 (3 CH_3 of *t*-Bu); HRMS (ESI$^+$): m/z calcd for $C_{29}H_{34}N_3O_3$ $[M+H]^+$: 472.2595. Found: 472.2592.

_N_-(_tert_-Butyl)-2-phenyl-_N'_-(_p_-tolyl)-1,2,3,4-tetrahydroisoquinoline-1-carboximidamide (2p)

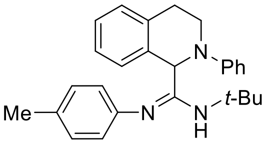

2p

A solution of iminium ion **1a** (1 equiv, 0.42 mmol, 122 mg), 4-methylaniline (1.5 equiv, 0.64 mmol, 68 mg) and *t*-butyl isocyanide (1.5 equiv, 0.64 mmol, 73 µL) in CH_3CN (4.2 mL, 0.1 M) was stirred at 30 °C for 24 h. The reaction mixture concentrated, and the residue was purified by column chromatography on alumine oxide with PE/Et_2O (from 100 to 98:2) to give **2p** (101 mg, 60%) as colourless oil. Rf 0.75 (PE/AcOEt 85:15); ^1H NMR (400 MHz, $CDCl_3$) δ 7.36 – 7.25 (m, 2H, 2 CH Ar of Ph), 7.21 – 7.02 (m, 7H, 3 CH Ar of Ph and 4 CH Ar of THIQ), 6.95 (d, J = 8.2 Hz, 2H, 2 CH of *p*-CH_3-Ph), 6.48 (d, J = 8.2 Hz, 2H, 2 CH of *p*-CH_3-Ph), 5.18 (s, 1H, CH), 4.88 (s, 1H, NH), 3.43 (dt, J = 10.0, 4.3 Hz, 1H, , 1 H of CH_2 THIQ), 3.23 (td, J = 10.5, 3.0 Hz, 1H, 1 H of CH_2 THIQ), 3.20 – 3.11 (m, 1H, 1 H of CH_2 THIQ), 2.80 (dt, J = 15.4, 3.4 Hz, 1H, 1 H of CH_2 THIQ), 2.25 (s, 3H, CH_3), 1.24 (s, 9H, 3

CH_3 of *t*-Bu); ^{13}C NMR (101 MHz, $CDCl_3$) δ 154.7 (Cq amidine), 151.0 (Cq near N), 148.0 (Cq near N), 135.2 (Cq Ar), 135.0 (Cq Ar), 130.4 (Cq Ar), 129.3 (2 CH Ar), 129.0 (2 CH Ar), 128.3 (CH Ar), 127.9 (CH Ar), 126.9 (CH Ar), 126.2 (CH Ar), 123.1 (CH Ar), 121.8 (2 CH Ar), 121.7 (2 CH Ar), 61.0 (CH of THIQ), 50.9 (Cq of *t*-Bu), 49.4 (CH_2 of THIQ), 30.0 (CH_2 of THIQ), 28.4 (3 CH_3 of *t*-Bu), 20.9 (CH_3).

***N*-(*tert*-Butyl)-2-(2-(2-((E)-*N*'-tert-butyl-*N*-phenylformimidamido)ethoxy)phenyl)-*N*'-(3,5-dimethoxyphenyl)-2-((3,5-dimethoxyphenyl)amino)acetimidamide (7)**

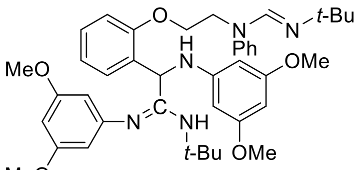

7

A solution of *N*-Ph-benzoxazepine **5** (1 equiv, 0.47 mmol, 106 mg), 3,5-dimethoxyaniline (1.5 equiv, 0.71 mmol, 109 mg) and $BrCCl_3$ (3 equiv, 1.41 mmol, 139 µL) in CH_3CN (2.4 mL, 0.2 M) in a vial was degassed with argon for 2 minutes, then *t*-butyl isocyanide (2.2 equiv, 1.03 mmol, 117 µL) was added and the vial sealed. The mixture was irradiated at 30 °C at 451 nm (blue LEDs) under magnetic stirring for 24 h. The reaction mixture was concentrated and the residue was purified by column chromatography on alumine oxide with PE/AcOEt (from 11:1 to 9:1) to give unreacted starting material **5** (51 mg, 48%) and **7** (32 mg, 10%) as brown oil. An analytical sample of pure compound **7** was obtained by preparative TLC (PE/AcOEt 9:1 + 1% of Et_3N). Compound **7**: Rf 0.59 (PE/AcOEt 9:1 + 1% of Et_3N); I.R.: ū (cm^{-1}) =3378, 2962, 2929, 1679, 1640, 1589, 1495, 1453, 1389, 1359, 1288, 1242, 1200, 1148, 1110, 1057, 925, 836, 814, 752, 694; ^1H NMR (400 MHz, $CDCl_3$) δ 7.76 (s, 1H, CH of formamidine), 7.55 (dd, *J* = 7.6, 1.7 Hz, 1H, CH Ar), 7.31 – 7.26 (m, 1H, CH Ar), 7.16 (dd, *J* = 8.3, 1.1 Hz, 1H, CH Ar), 7.14 – 7.07 (m, 2H, 2 CH Ar), 7.00 – 6.91 (m, 2H, 2 CH Ar), 6.90 – 6.85 (m, 2H, 2 CH Ar), 5.98 (t, *J* = 2.1 Hz, 1H, CH Ar), 5.92 (bs, 1H, NH of amidine), 5.91 (t, *J* = 2.3 Hz, 1H, CH Ar), 5.87 (d, *J* = 2.1 Hz, 2H, 2 CH Ar), 5.59 (d, *J* = 2.3 Hz, 2H, 2 CH

Ar), 5.28 (d, J = 1.7 Hz, 1H, <u>CH</u>NH), 4.20 – 4.13 (m, 1H, 1 H of CH_2), 3.99 – 3.83 (m, 2H, 2 H of CH_2), 3.77 (s, 7H, 2 OCH_3 and CH<u>NH</u>), 3.76 – 3.70 (m, 1H, 1 H of CH_2), 3.42 (s, 6H, 2 OCH_3), 1.48 (s, 9H, 3 CH_3 of *t*-Bu), 1.22 (s, 9H, 3 CH_3 of *t*-Bu); ^{13}C NMR (101 MHz, $CDCl_3$) δ 161.6 (2 Cq near O), 160.7 (2 Cq near O), 156.4 (Cq near O), 154.9 (Cq amidine), 153.1 (Cq near N), 149.7 (Cq near N), 146.4 (CH formamidine), 145.6 (Cq near N), 129.7 (CH Ar), 129.3 (2 CH Ar of Ph), 128.5 (CH Ar), 128.4 (Cq Ar), 122.9 (CH Ar of Ph), 120.8 (CH Ar), 119.9 (2 CH Ar of Ph), 112.8 (CH Ar), 100.0 (2 CH Ar), 94.8 (CH Ar), 92.6 (2 CH Ar), 92.0 (CH Ar), 64.7 (CH_2), 55.3 (2 OCH_3), 54.9 (2 OCH_3), 54.1 (Cq of *t*-Bu), 53.4 (CHNH), 51.1 (Cq of *t*-Bu), 45.9 (CH_2), 31.1 (3 CH_3 of *t*-Bu), 28.6 (3 CH_3 of *t*-Bu); HRMS (ESI^+): m/z calcd for $C_{41}H_{54}N_5O_5$ $[M+H]^+$: 696.4119. Found: 696.4124.

References

1. Duncan, J. A.; Bosse, M. L.; Masnovi, J. M., *J. Org. Chem.* **1980,** *45* (16), 3176-3181.
2. Franz, J. F.; Kraus, W. B.; Zeitler, K., *Chemical Communications* **2015,** *51* (39), 8280-8283.
3. Li, T.; Sun, Z.; Zhang, S.; Ma, Q.; Chen, Y.; Yuan, Y.; Jia, X., *J Org Chem* **2024,** *89* (4), 2516-2524.
4. Ueda, H.; Yoshida, K.; Tokuyama, H., *Org Lett* **2014,** *16* (16), 4194-7.
5. Pinna, A.; Basso, A.; Lambruschini, C.; Moni, L.; Riva, R.; Rocca, V.; Banfi, L., *RSC Adv* **2020,** *10* (2), 965-972.
6. Szaloki Vargane, D.; Toth, L.; Buglyo, B.; Kiss-Szikszai, A.; Mandi, A.; Matyus, P.; Antus, S.; Chen, Y.; Li, D.; Tao, L.; Zhang, H.; Kurtan, T., *Molecules* **2020,** *25* (6).
7. Xia, Q.; Yang, J.; Zhang, S.; Zhang, J.; Li, Z.; Wang, J.; Chen, X., *J Am Chem Soc* **2023,** *145* (11), 6123-6134.

Copies of ^{1}H, ^{13}C and ^{19}F NMR spectra of compounds

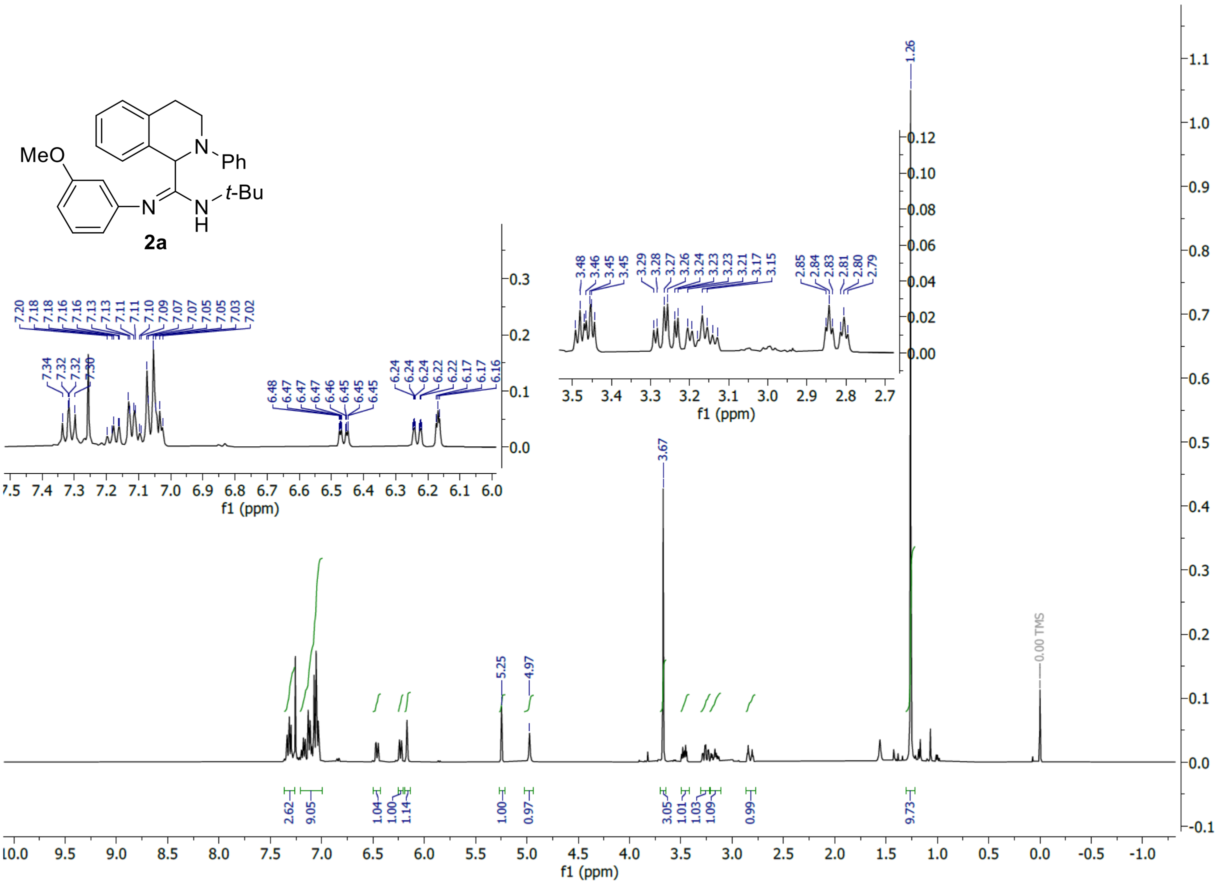

2a

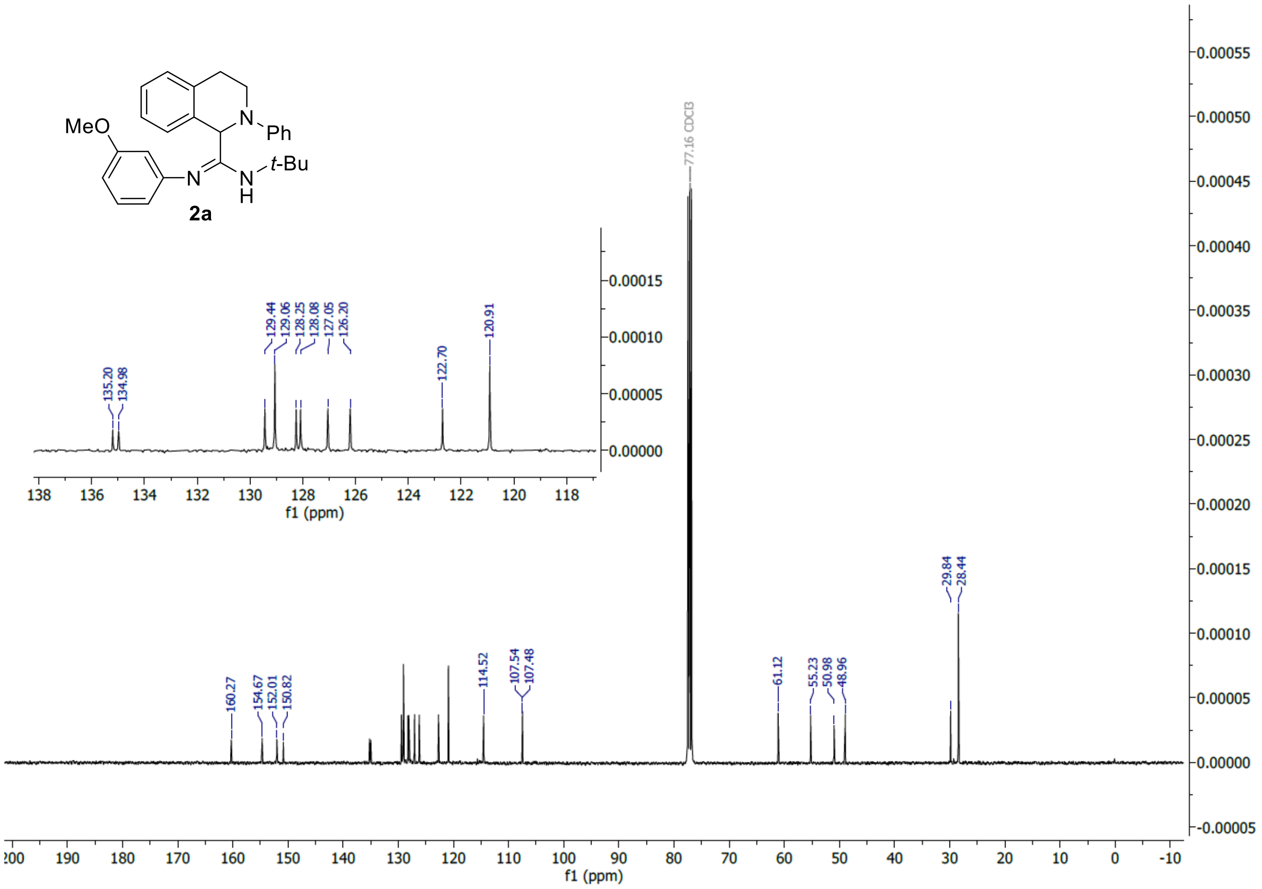
MeO
N
Ph
N
t-Bu
N
H
2a
f1 (ppm)
77.16 CDCl3

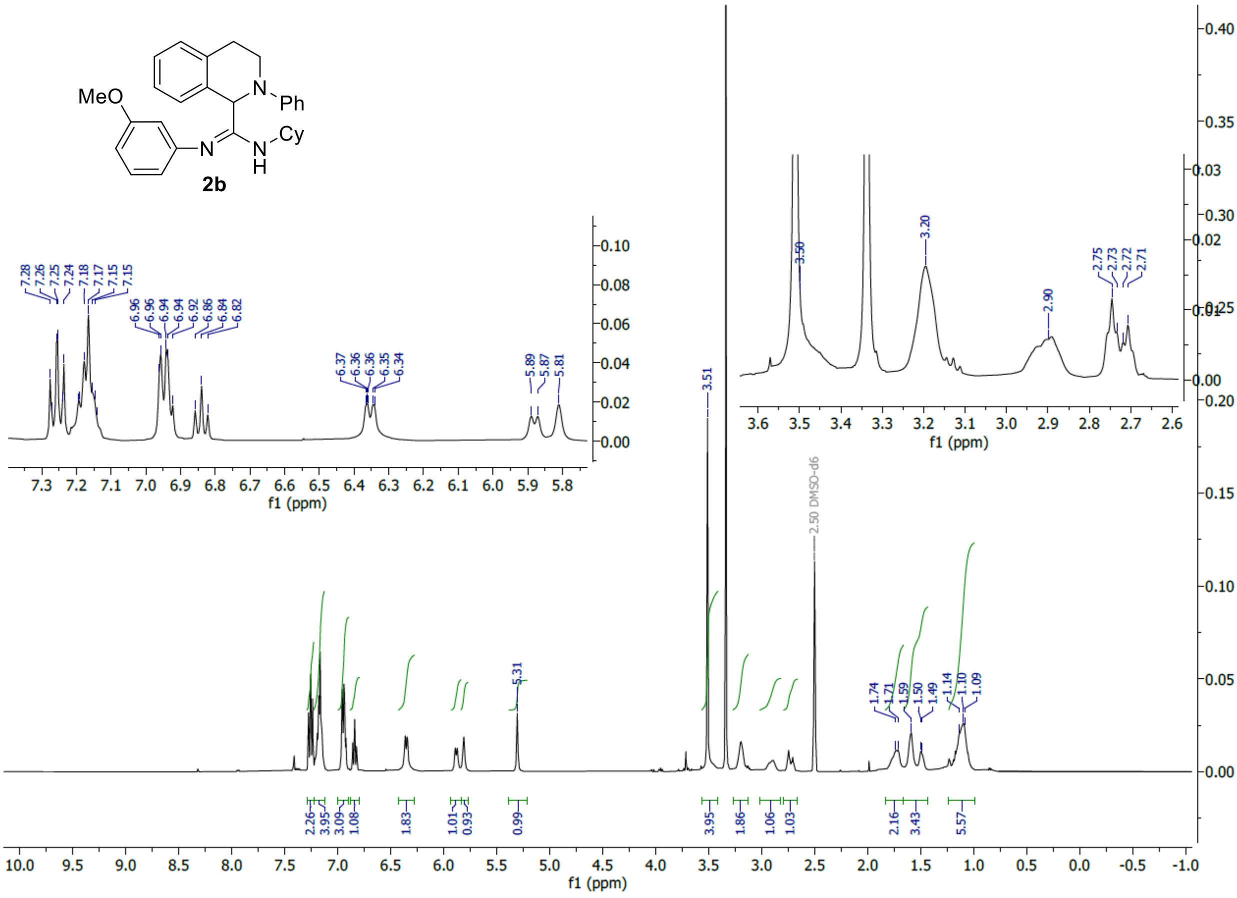
MeO
N
Ph
N
Cy
N
H
2b
f1 (ppm)
2.50 DMSO-d6

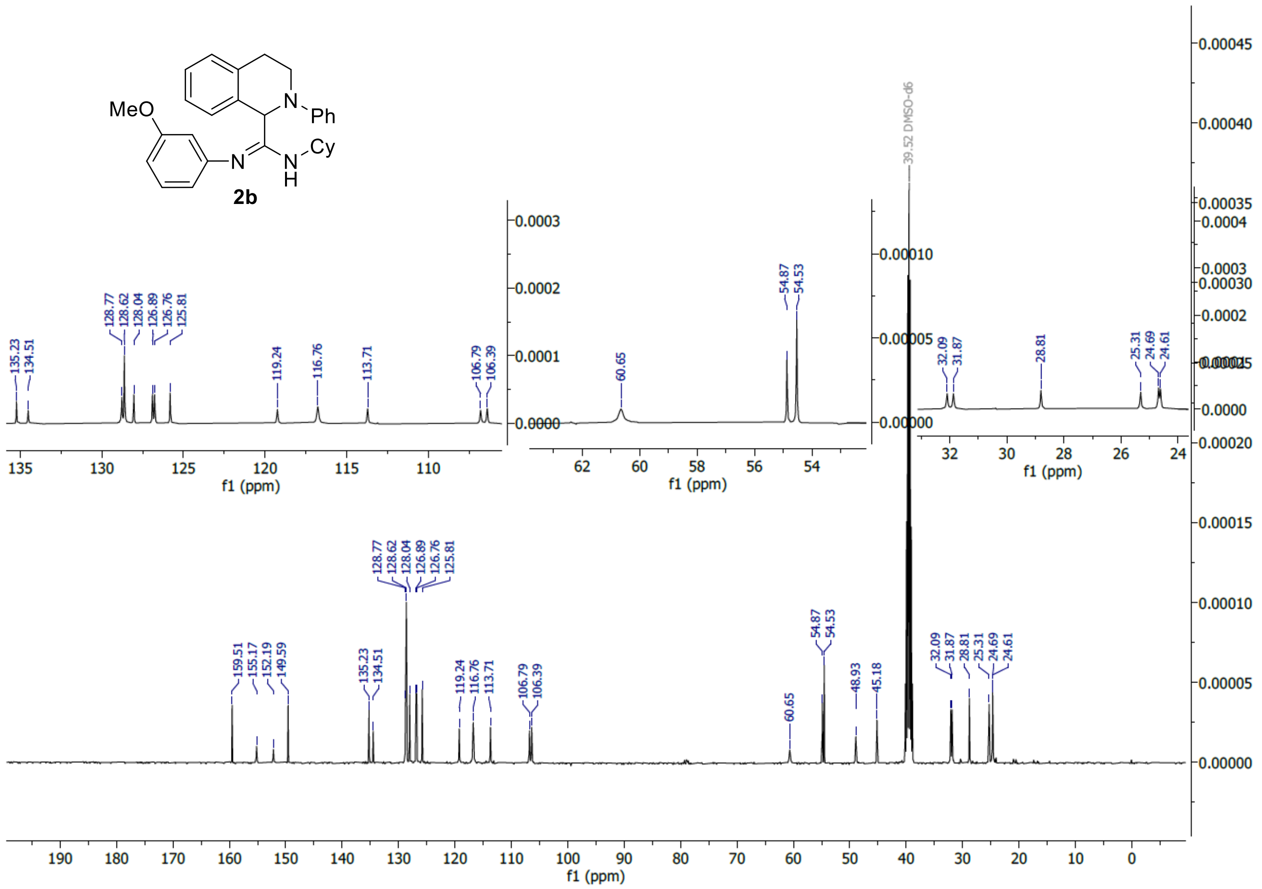
MeO
N
Ph
N
H
Cy
2b
39.52 DMSO-d6
f1 (ppm)

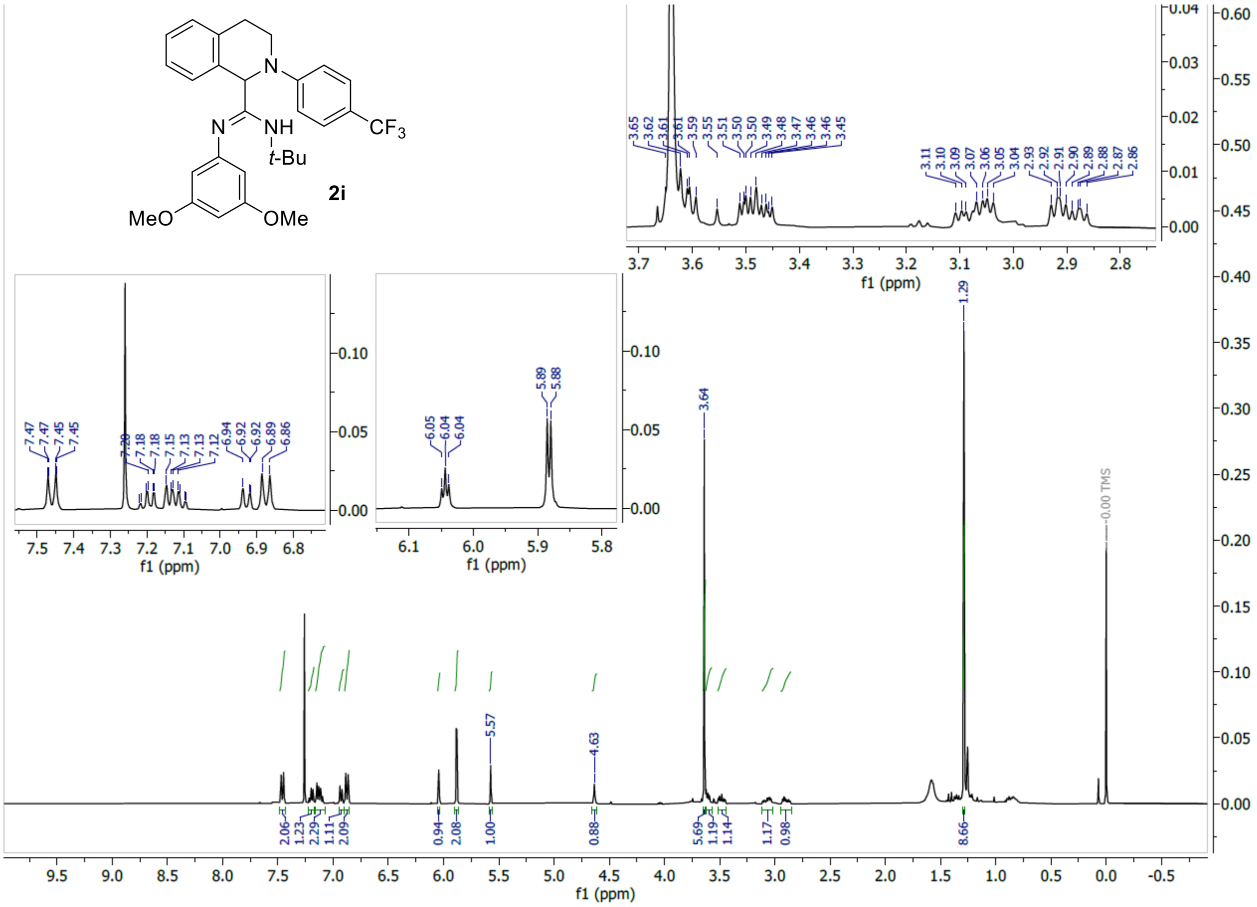
N
CF3
NH
t-Bu
MeO
OMe
2i
0.00 TMS
f1 (ppm)

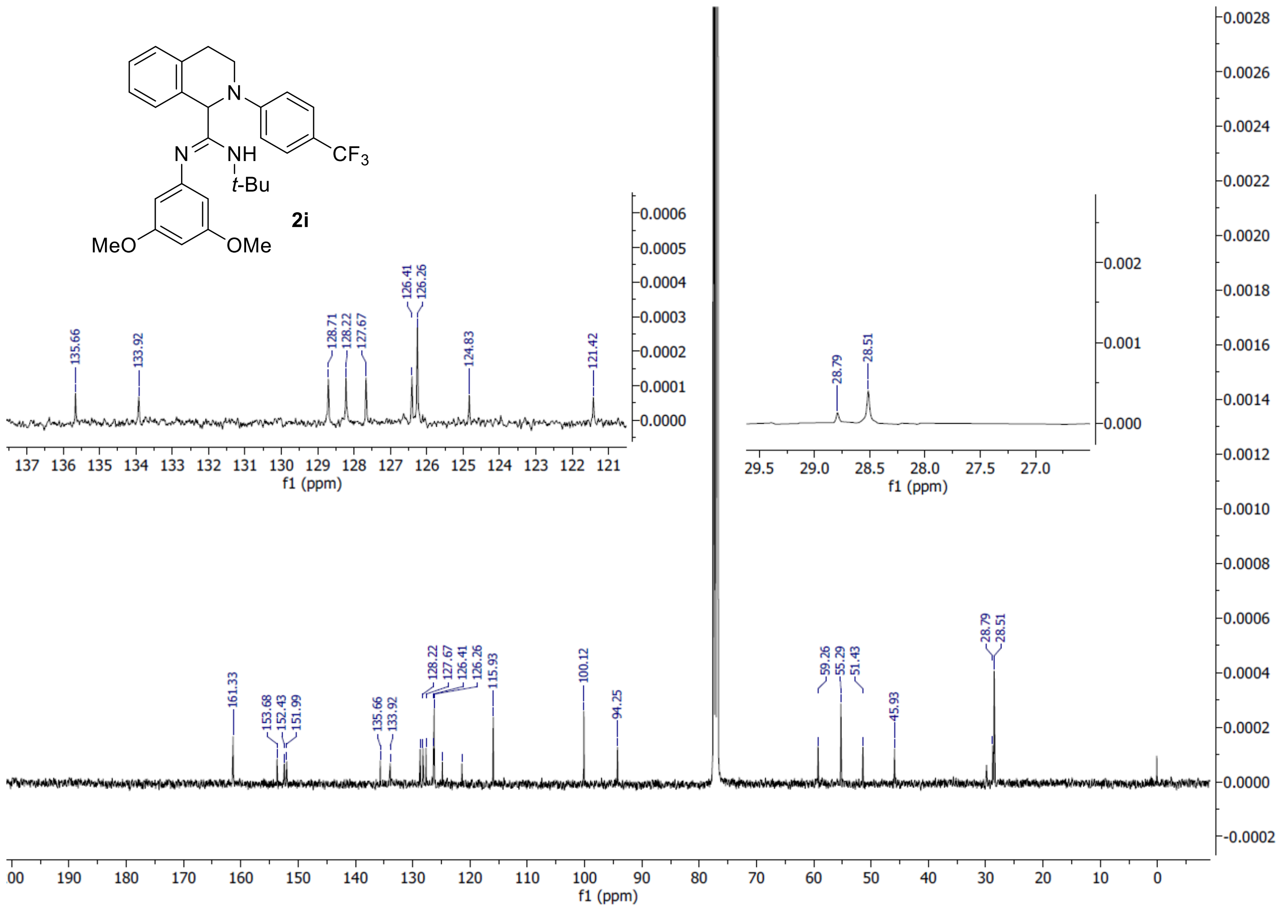

MeO
OMe
N
NH
t-Bu
CF3
2i
161.33
153.68
152.43
151.99
135.66
133.92
128.22
127.67
126.41
126.26
115.93
100.12
94.25
59.26
55.29
51.43
45.93
28.79
28.51
128.71
124.83
121.42
f1 (ppm)

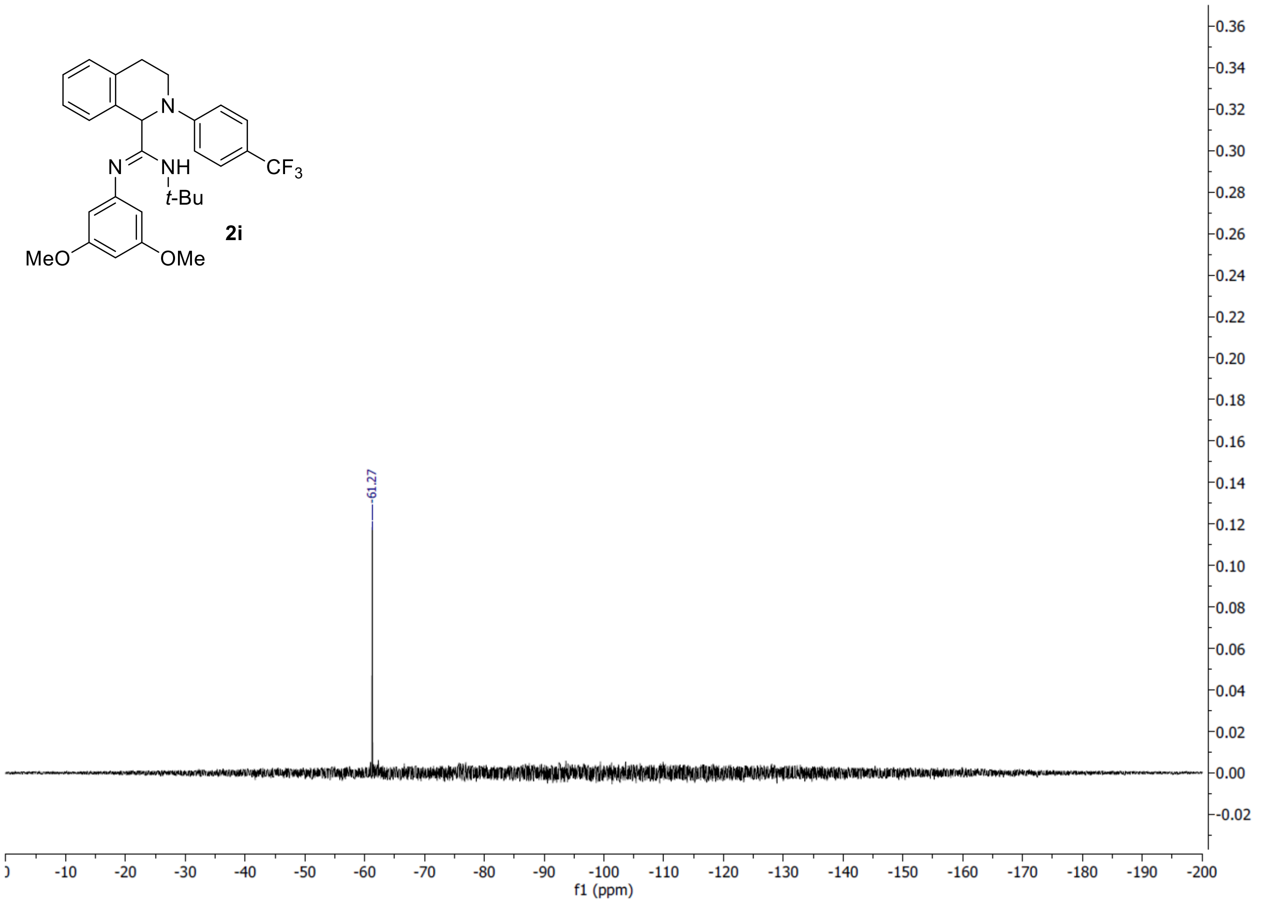

MeO
OMe
N
NH
t-Bu
CF3
2i
-61.27
f1 (ppm)

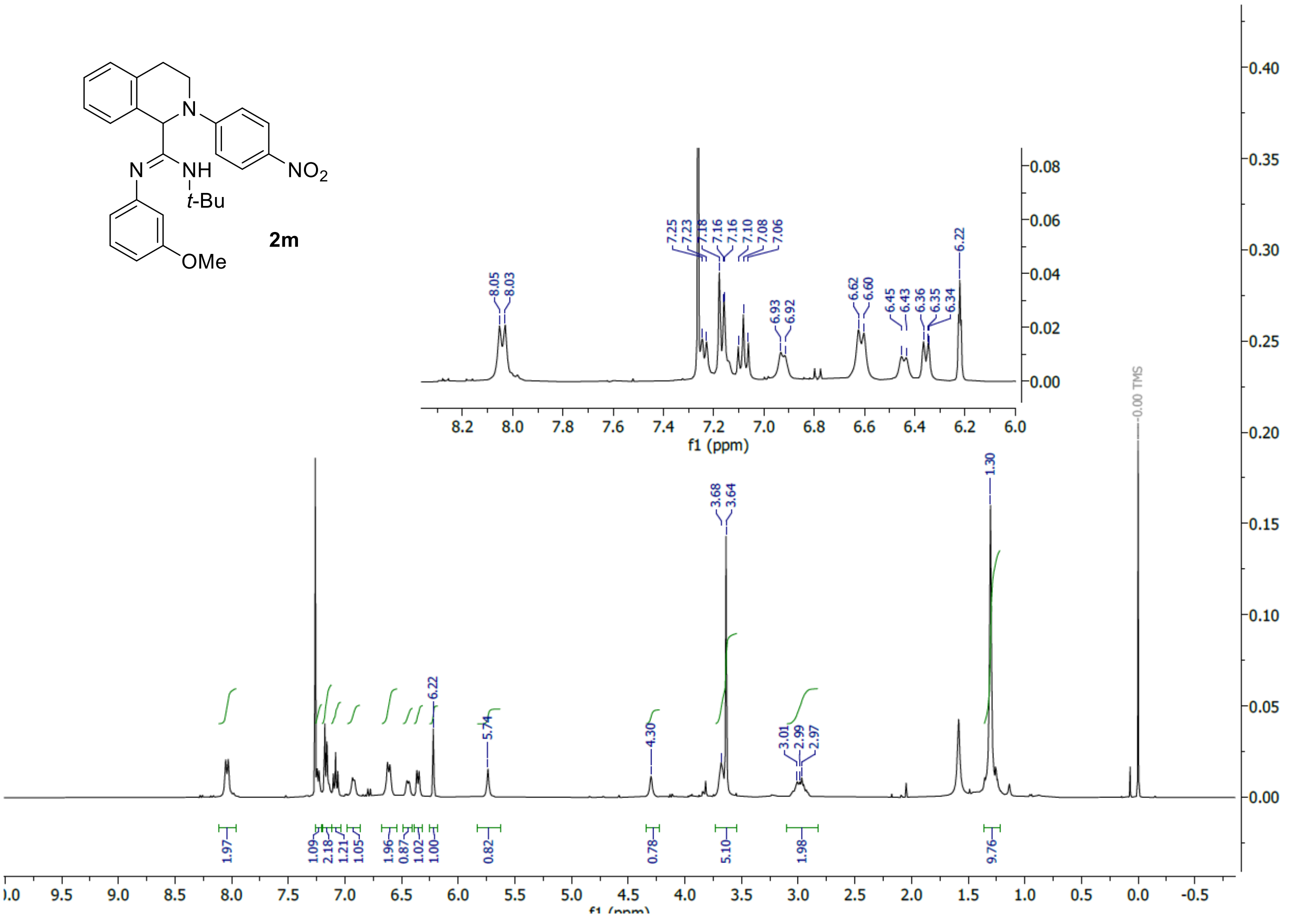

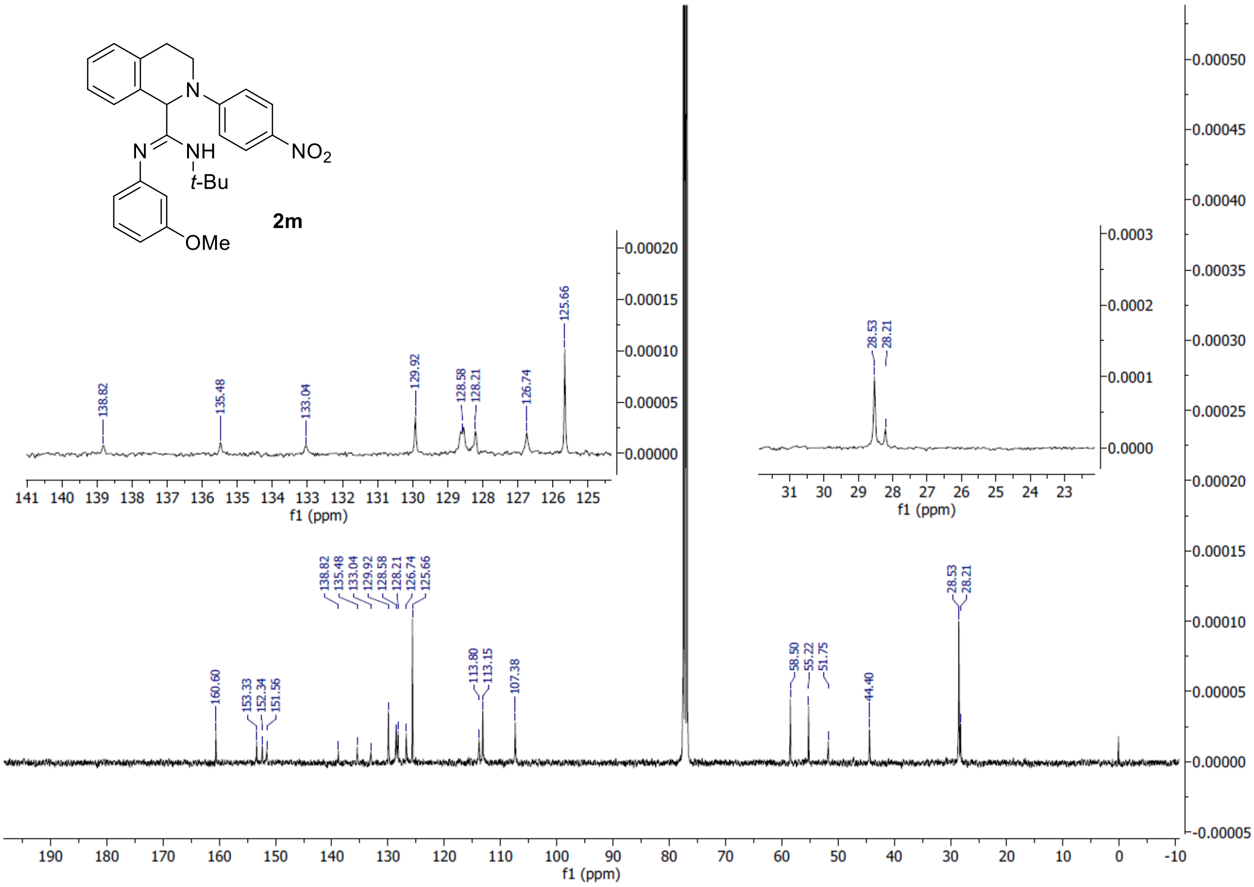

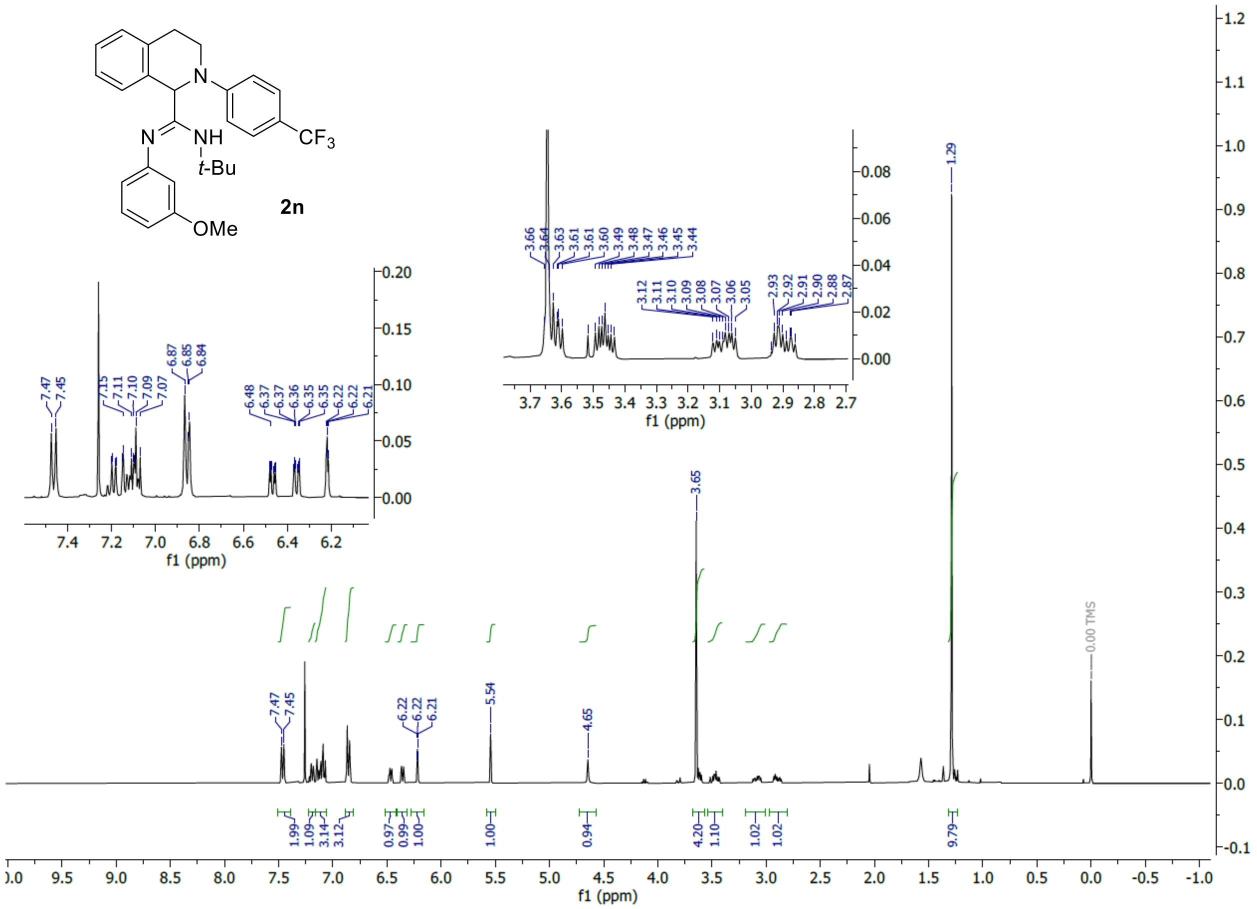

2n
N
NH
t-Bu
CF3
OMe
7.47 7.45 7.15 7.11 7.10 7.09 7.07 6.87 6.85 6.84 6.48 6.37 6.37 6.36 6.35 6.35 6.22 6.22 6.21
3.66 3.64 3.63 3.61 3.61 3.60 3.49 3.48 3.47 3.46 3.45 3.44 3.12 3.11 3.10 3.09 3.08 3.07 3.06 3.05 2.93 2.92 2.91 2.90 2.88 2.87
1.29
3.65
5.54
4.65
0.00 TMS
1.99 1.09 3.14 3.12 0.97 0.99 1.00 1.00 0.94 4.20 1.10 1.02 1.02 9.79
f1 (ppm)

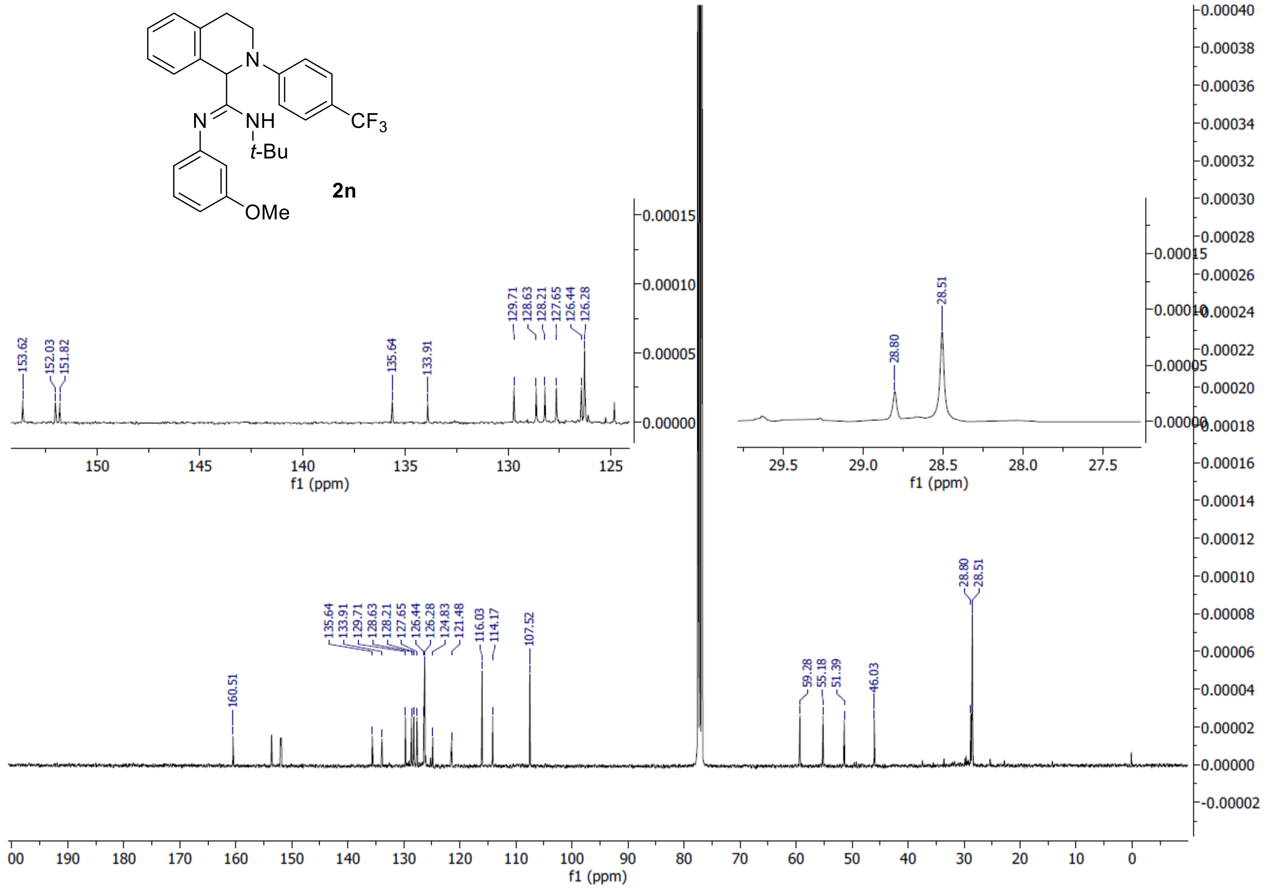

2n
N
NH
t-Bu
CF3
OMe
153.62 152.03 151.82 135.64 133.91 129.71 128.63 128.21 127.65 126.44 126.28
28.80 28.51
160.51
135.64 133.91 129.71 128.63 128.21 127.65 126.44 126.28 124.83 121.48 116.03 114.17 107.52
59.28 55.18 51.39 46.03
f1 (ppm)

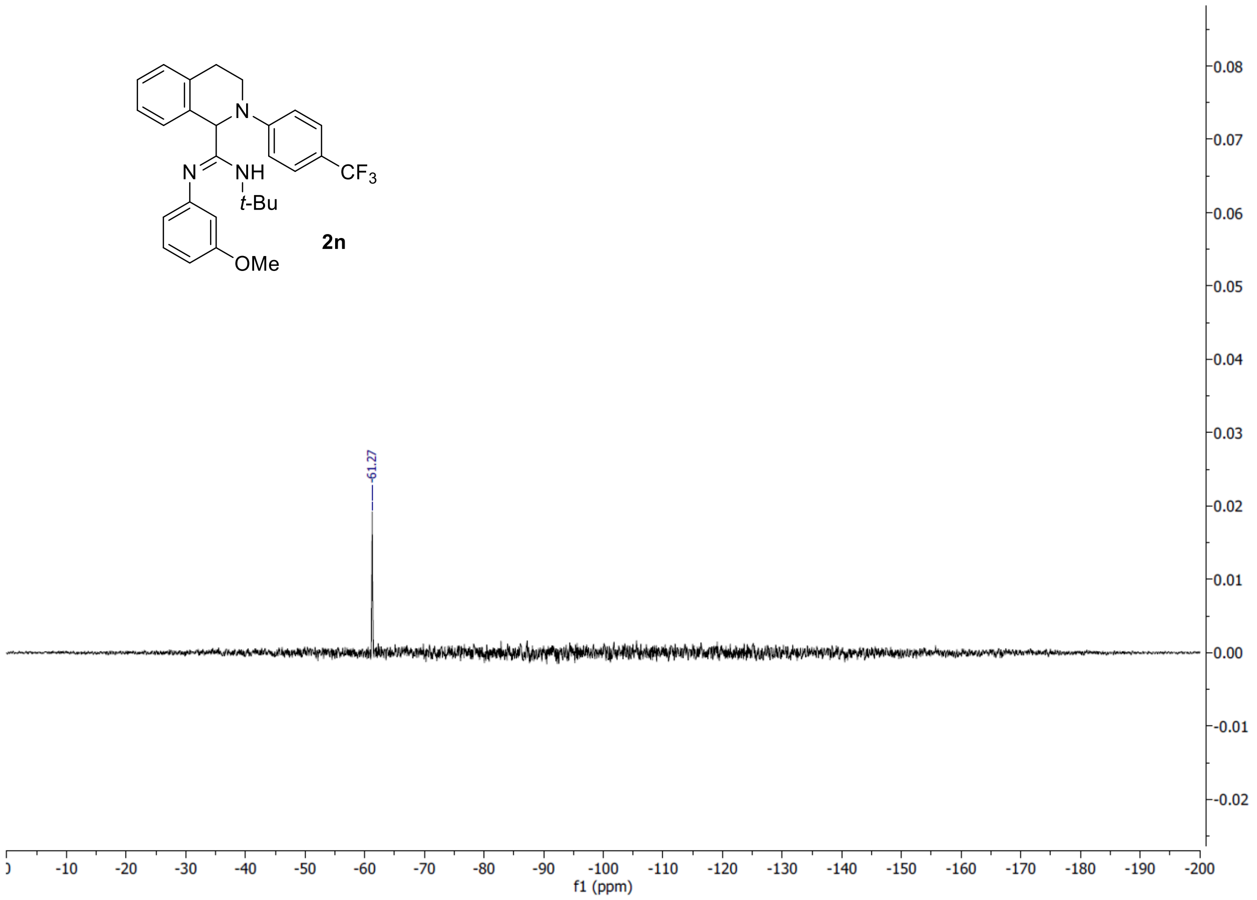

CF3
NH
t-Bu
OMe
2n
-61.27
f1 (ppm)

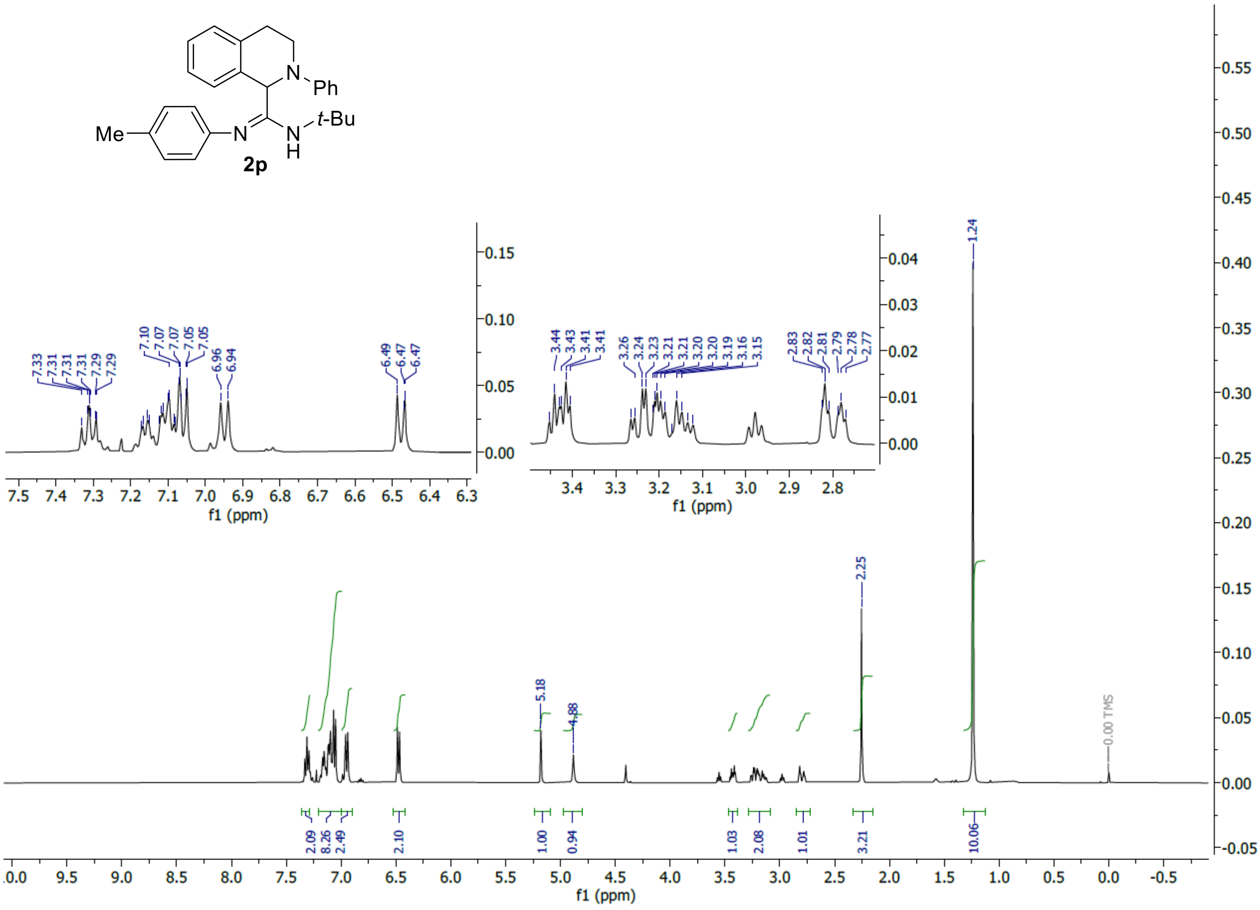

Me
N
Ph
t-Bu
2p
1.24
2.25
5.18
4.88
0.00 TMS
f1 (ppm)

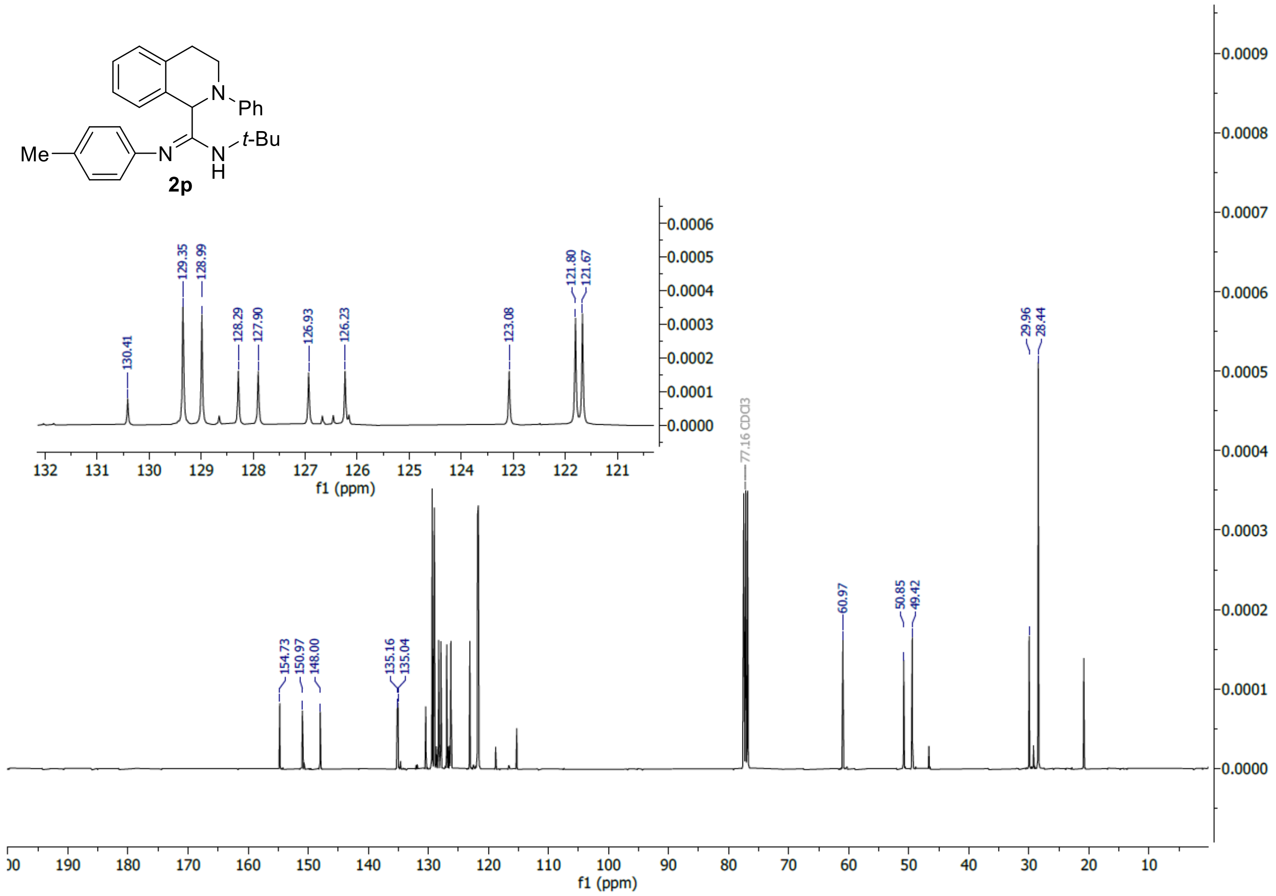
Me
N
N
Ph
t-Bu
H
2p
130.41
129.35
128.99
128.29
127.90
126.93
126.23
123.08
121.80
121.67
f1 (ppm)
154.73
150.97
148.00
135.16
135.04
77.16 CDCl3
60.97
50.85
49.42
29.96
28.44
f1 (ppm)

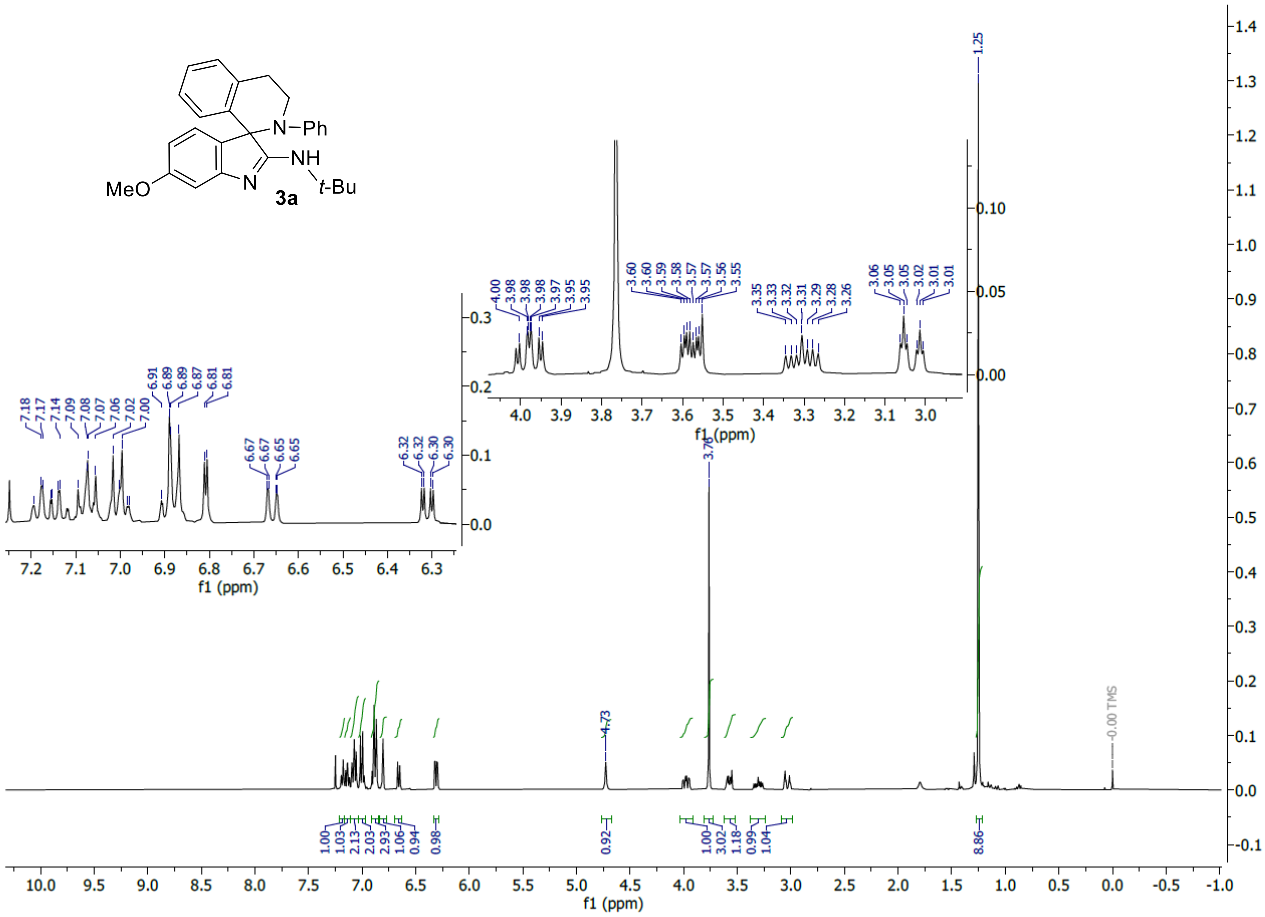
N−Ph
NH
t-Bu
MeO
N
3a
7.18
7.17
7.14
7.09
7.08
7.07
7.06
7.02
7.00
6.91
6.89
6.89
6.87
6.81
6.81
6.67
6.67
6.65
6.65
6.32
6.32
6.30
6.30
f1 (ppm)
4.00
3.98
3.98
3.98
3.97
3.95
3.95
3.60
3.60
3.59
3.58
3.57
3.57
3.56
3.55
3.35
3.33
3.32
3.31
3.29
3.28
3.26
3.06
3.05
3.05
3.02
3.01
3.01
f1 (ppm)
3.76
1.25
4.73
0.00 TMS
1.00
1.03
2.13
2.03
2.93
1.06
0.94
0.98
0.92
1.00
3.02
1.18
0.99
1.04
8.86
f1 (ppm)

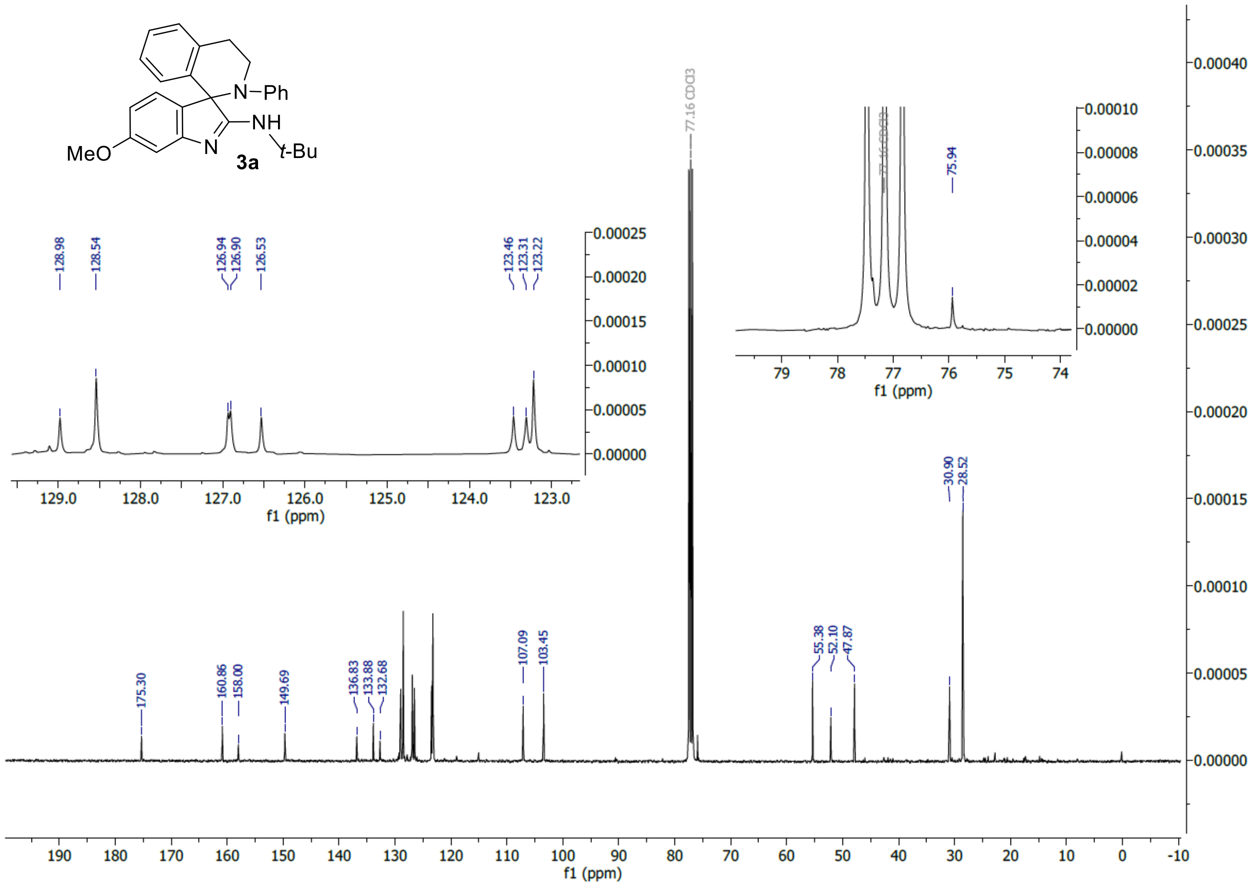

3a
MeO
N
NH
t-Bu
N–Ph
77.16 CDCl3
175.30
160.86
158.00
149.69
136.83
133.88
132.68
107.09
103.45
55.38
52.10
47.87
30.90
28.52
75.94
f1 (ppm)

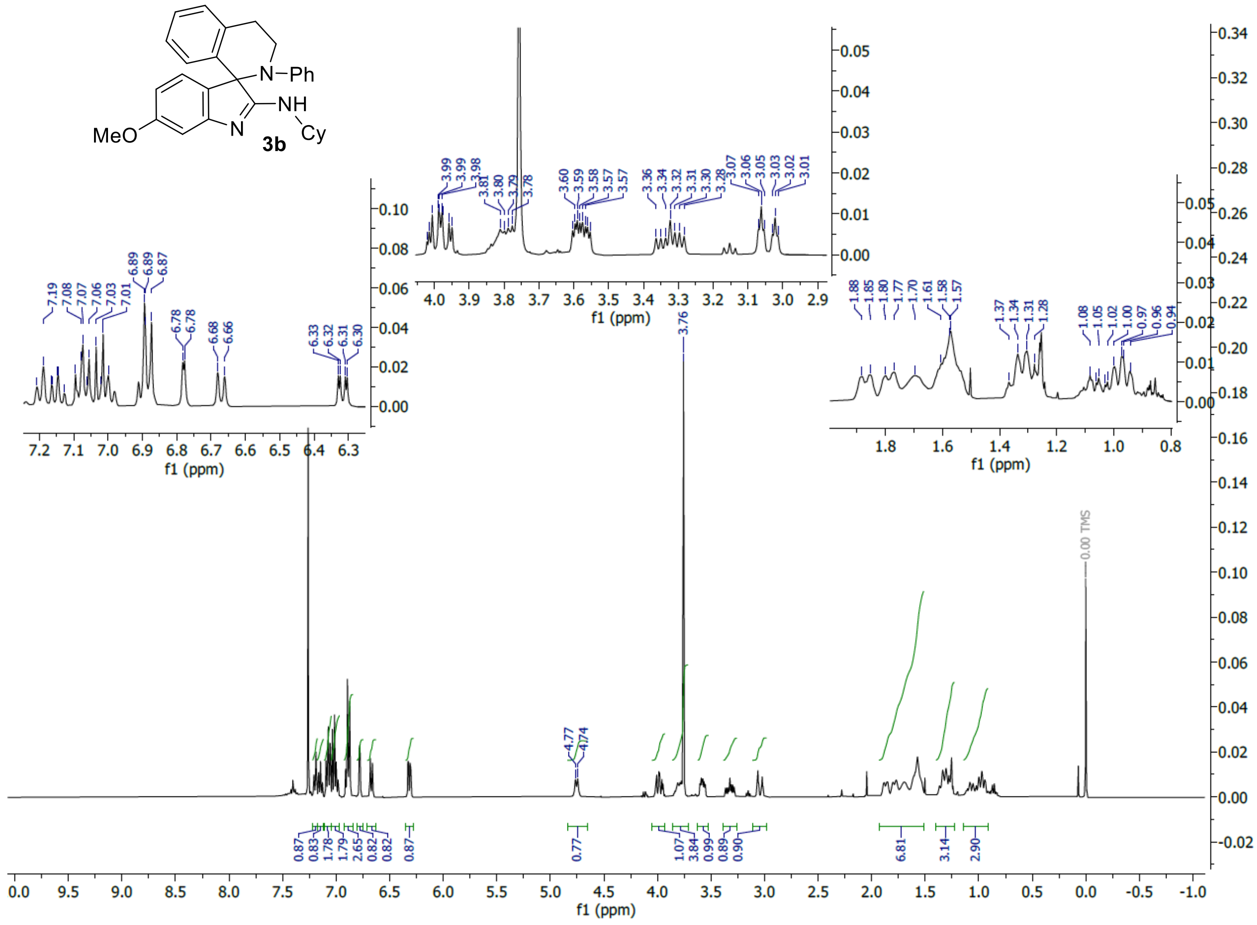

3b
MeO
N
NH
Cy
N–Ph
3.76
4.77
4.74
0.00 TMS
f1 (ppm)

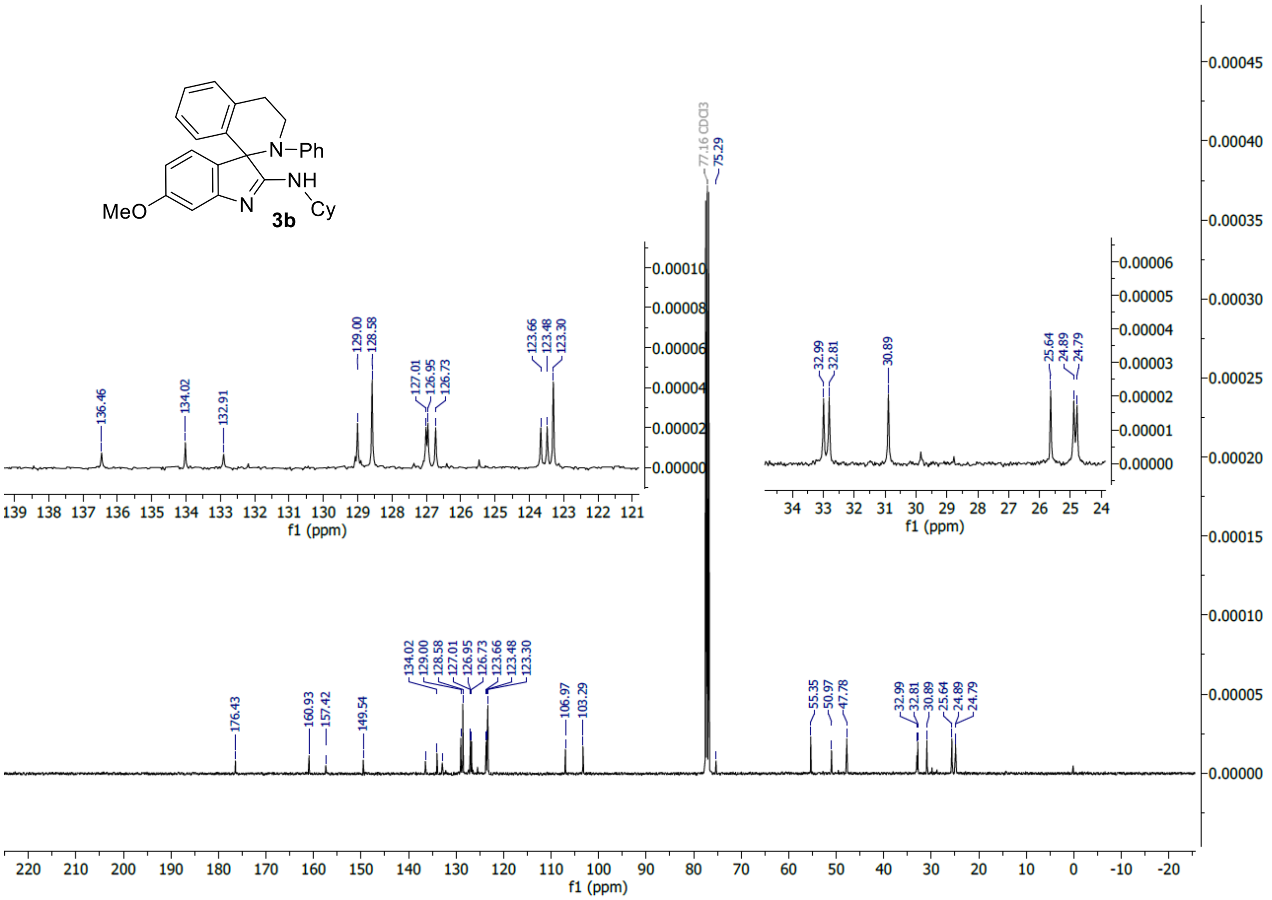

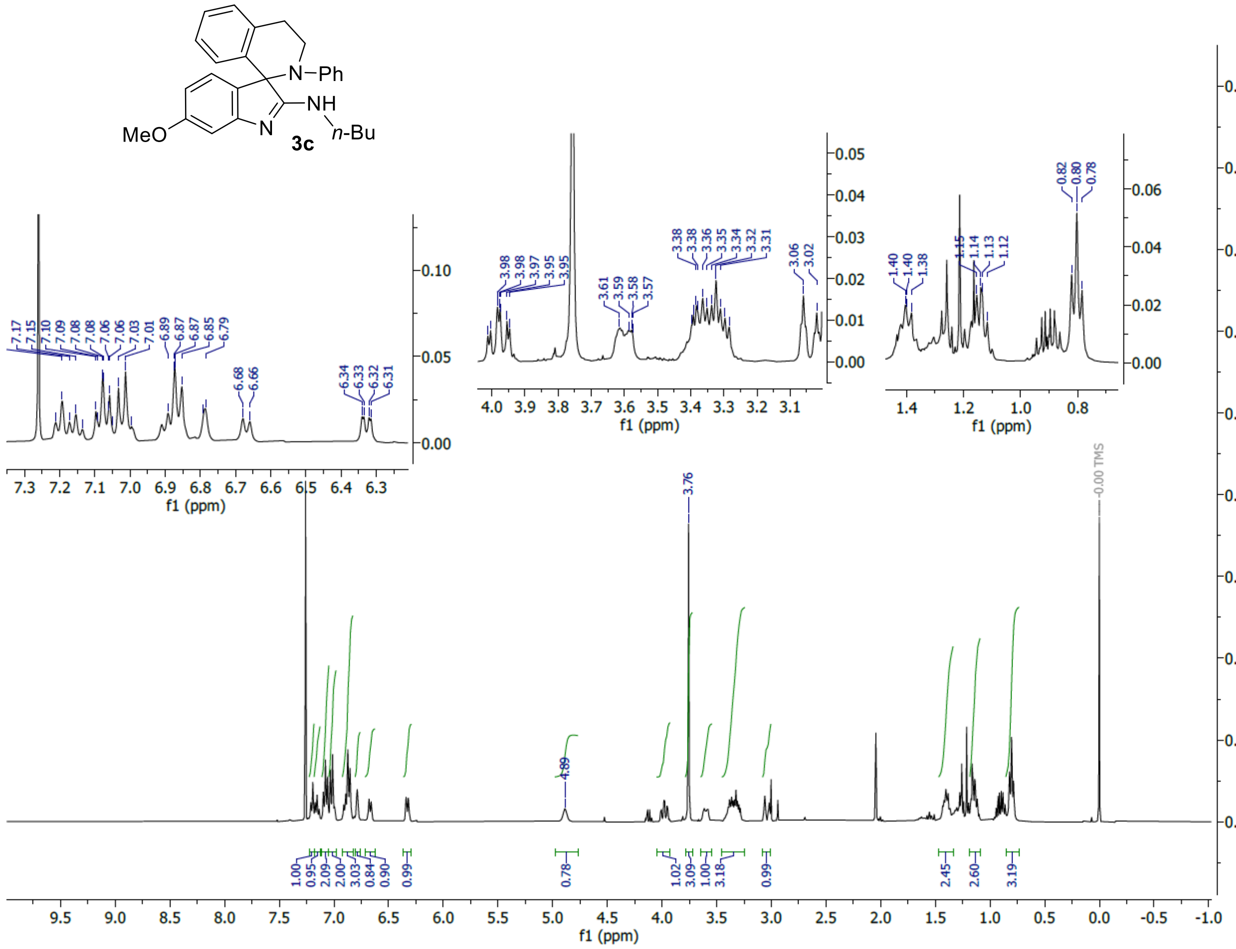

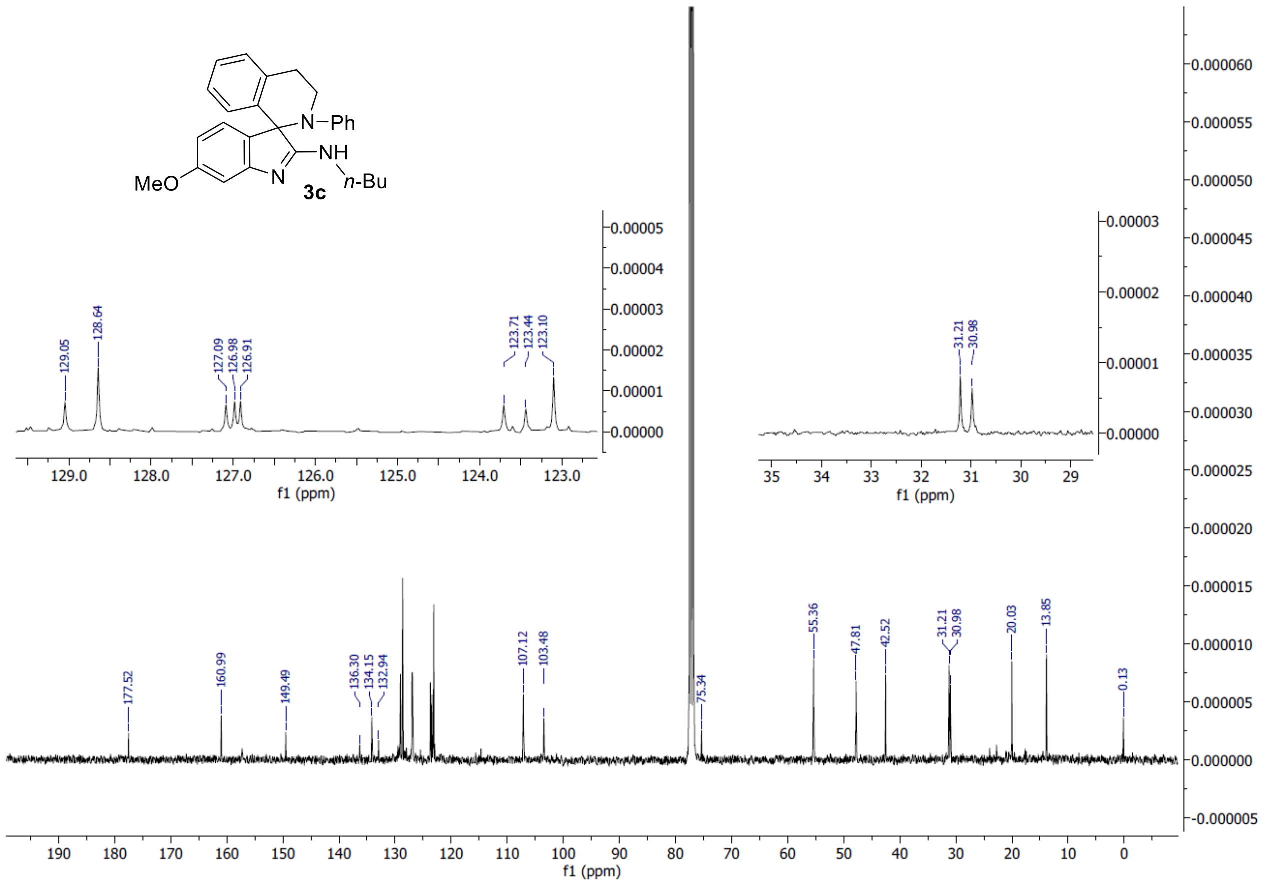

N–Ph
NH
MeO
N
n-Bu
3c
f1 (ppm)

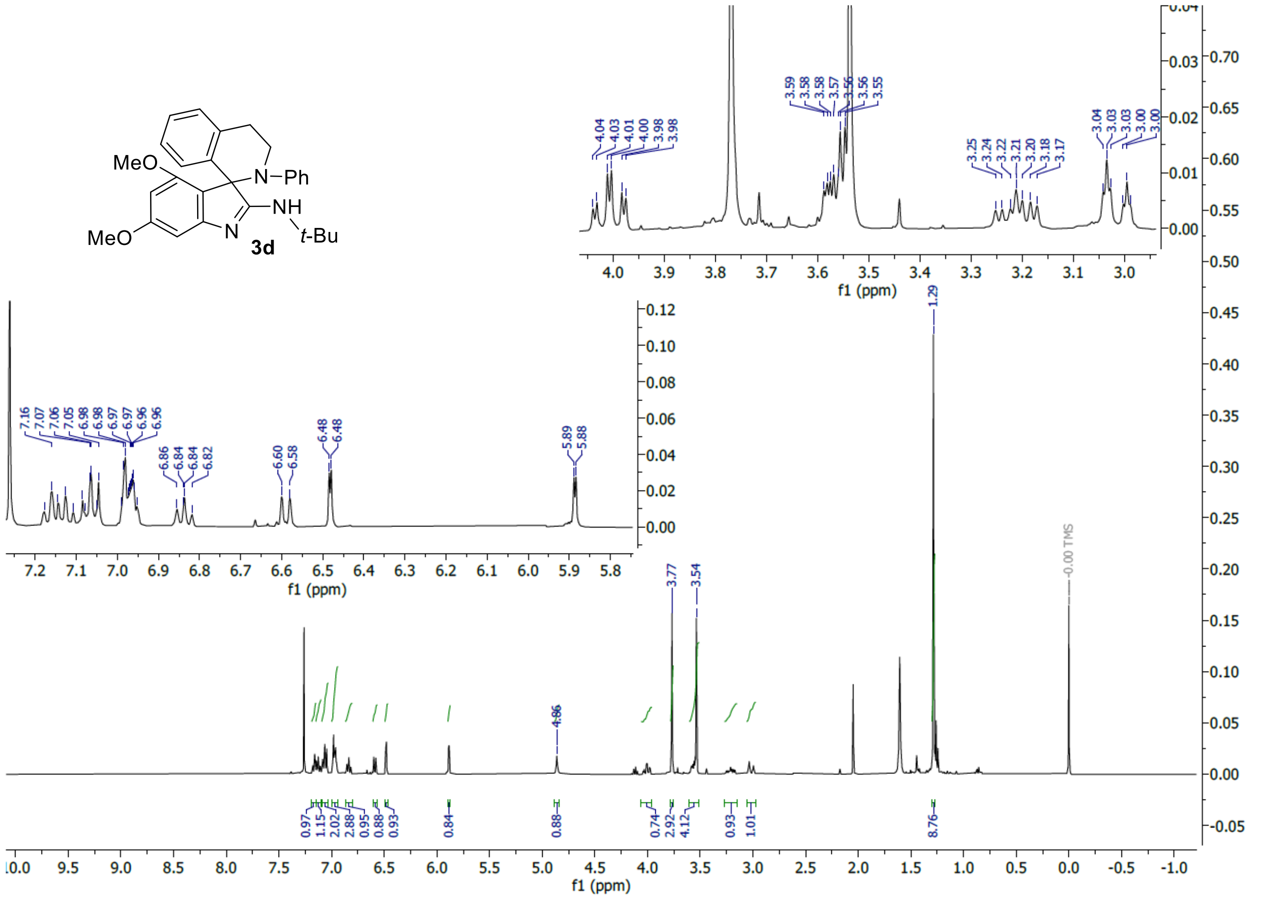

MeO
N–Ph
NH
MeO
N
t-Bu
3d
f1 (ppm)

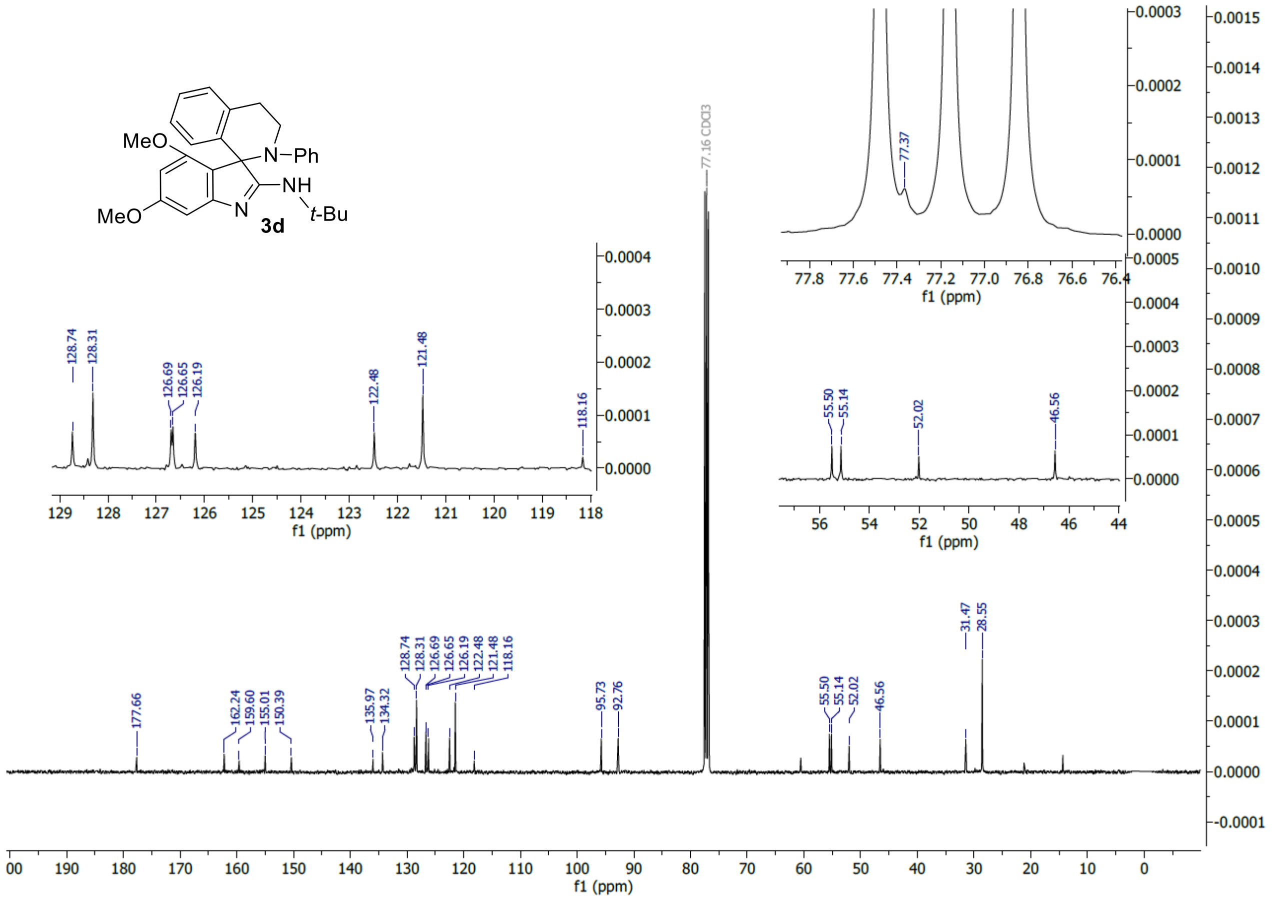
MeO
N−Ph
NH
MeO
N
3d
t-Bu
77.16 CDCl3
f1 (ppm)

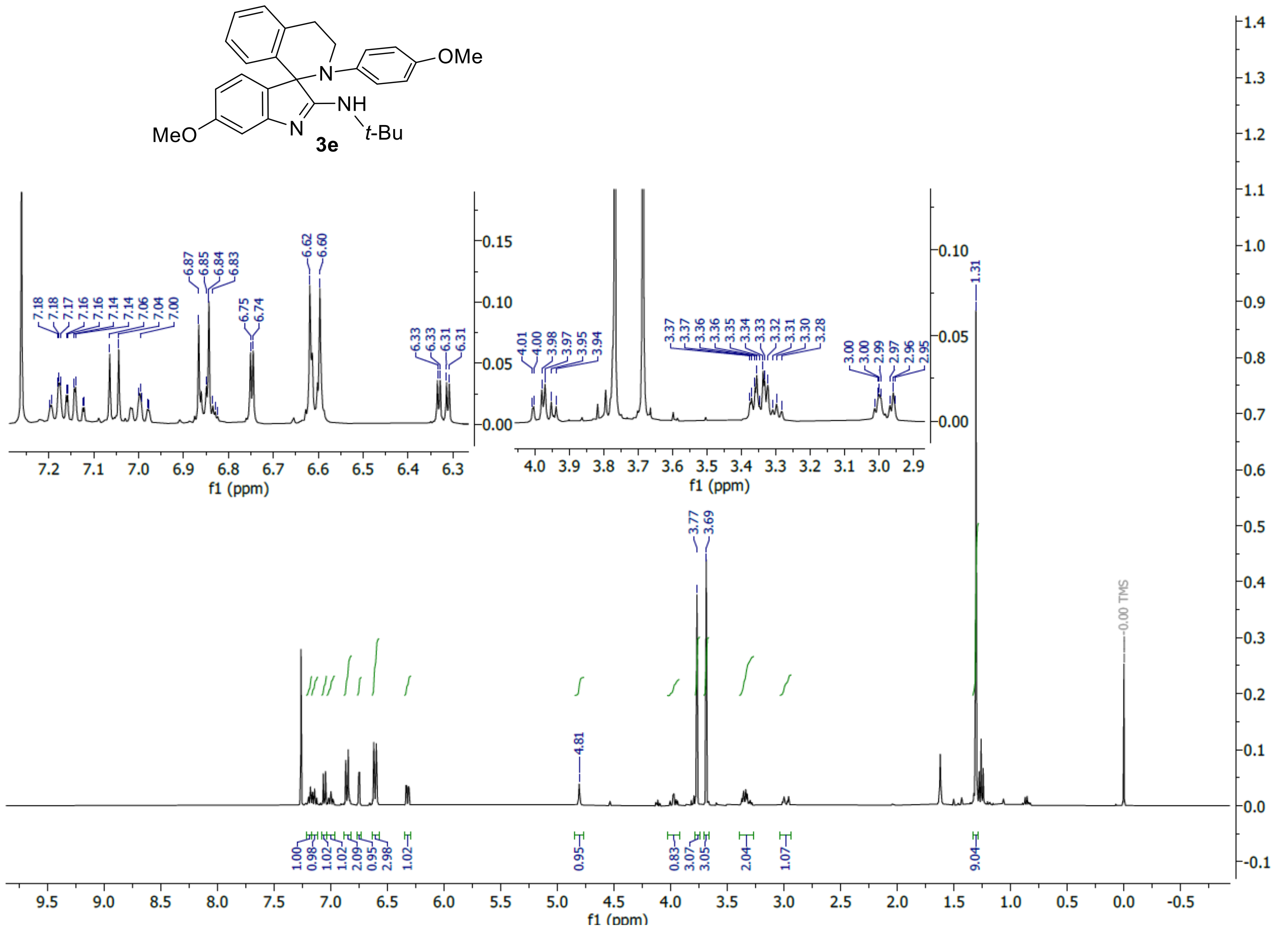
N
OMe
NH
MeO
N
3e
t-Bu
0.00 TMS
f1 (ppm)

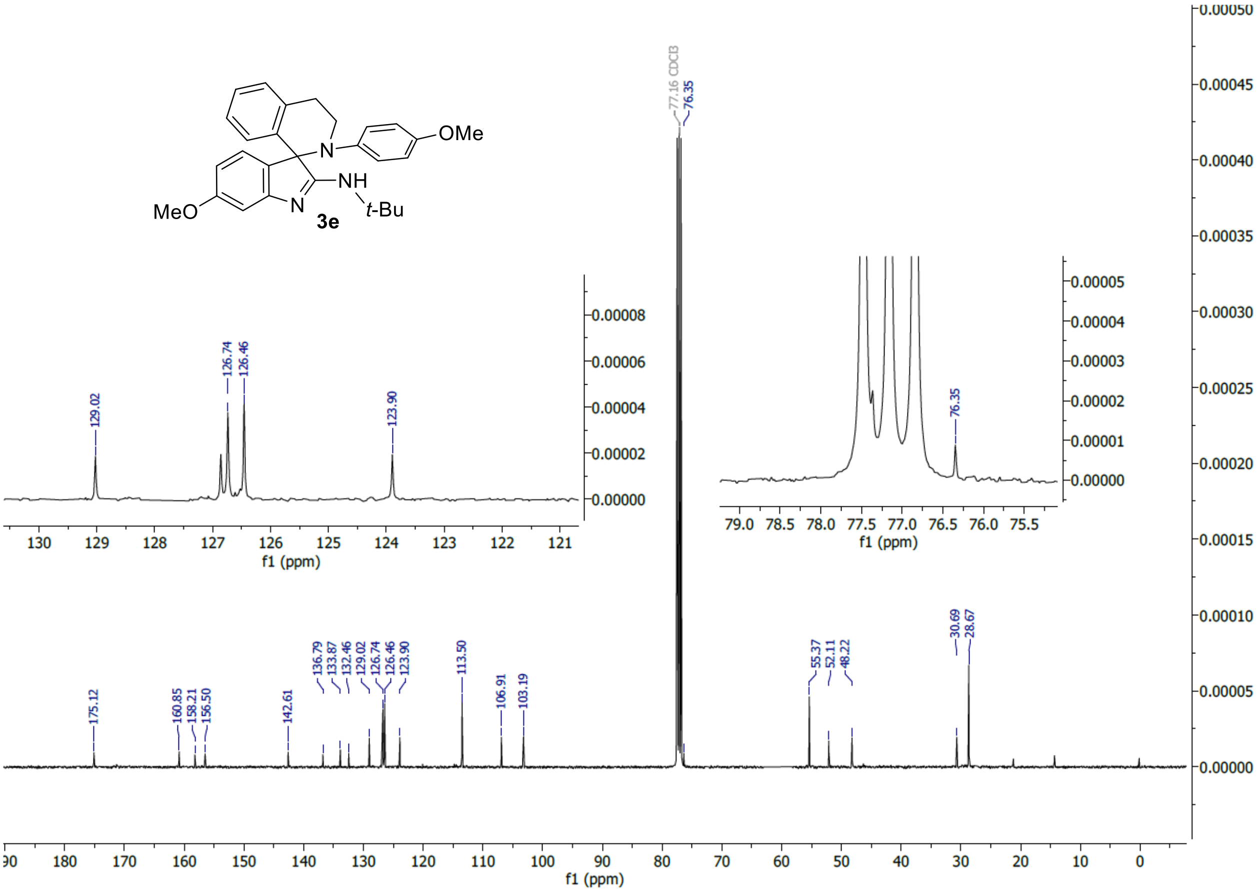
3e
MeO
OMe
NH
t-Bu
f1 (ppm)

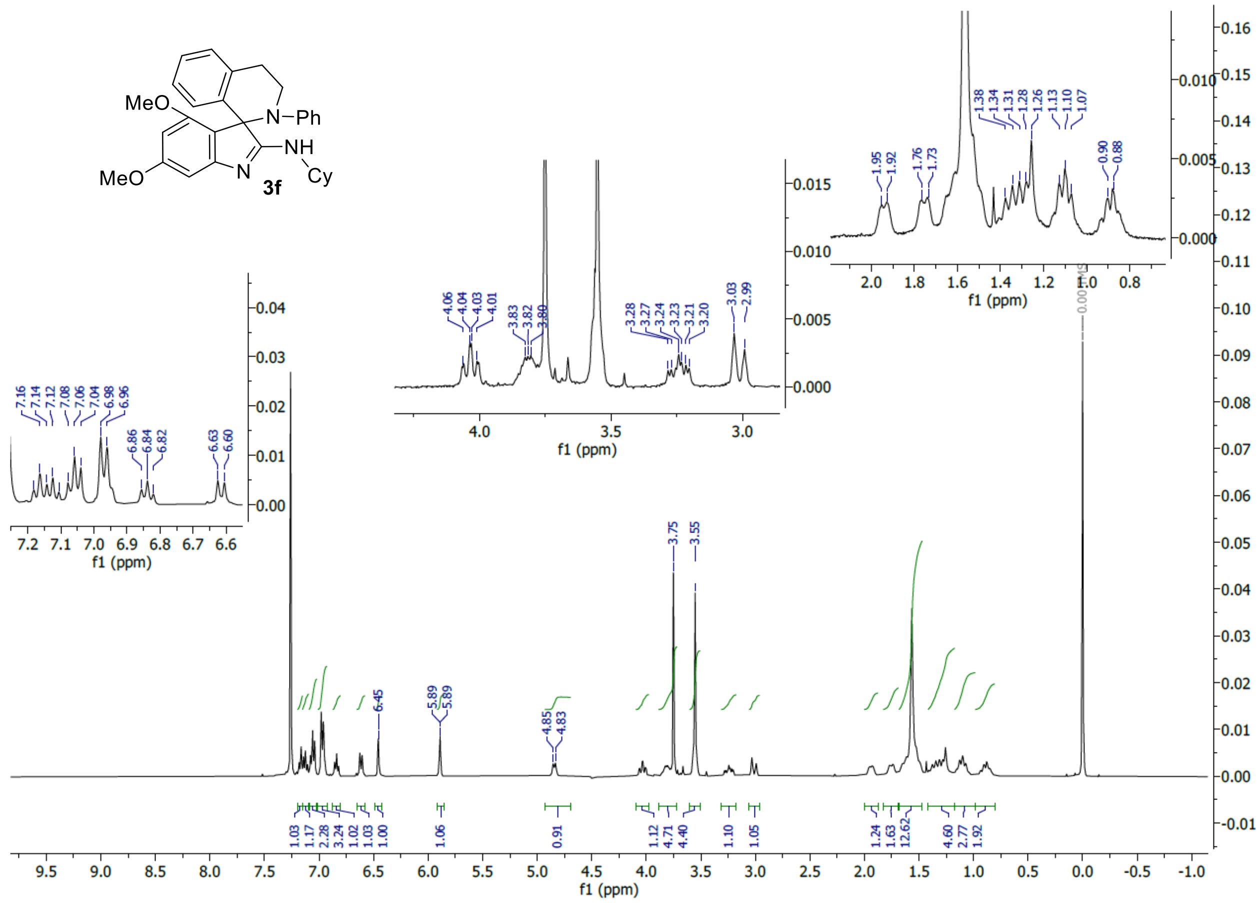
3f
MeO
N–Ph
NH
Cy
f1 (ppm)

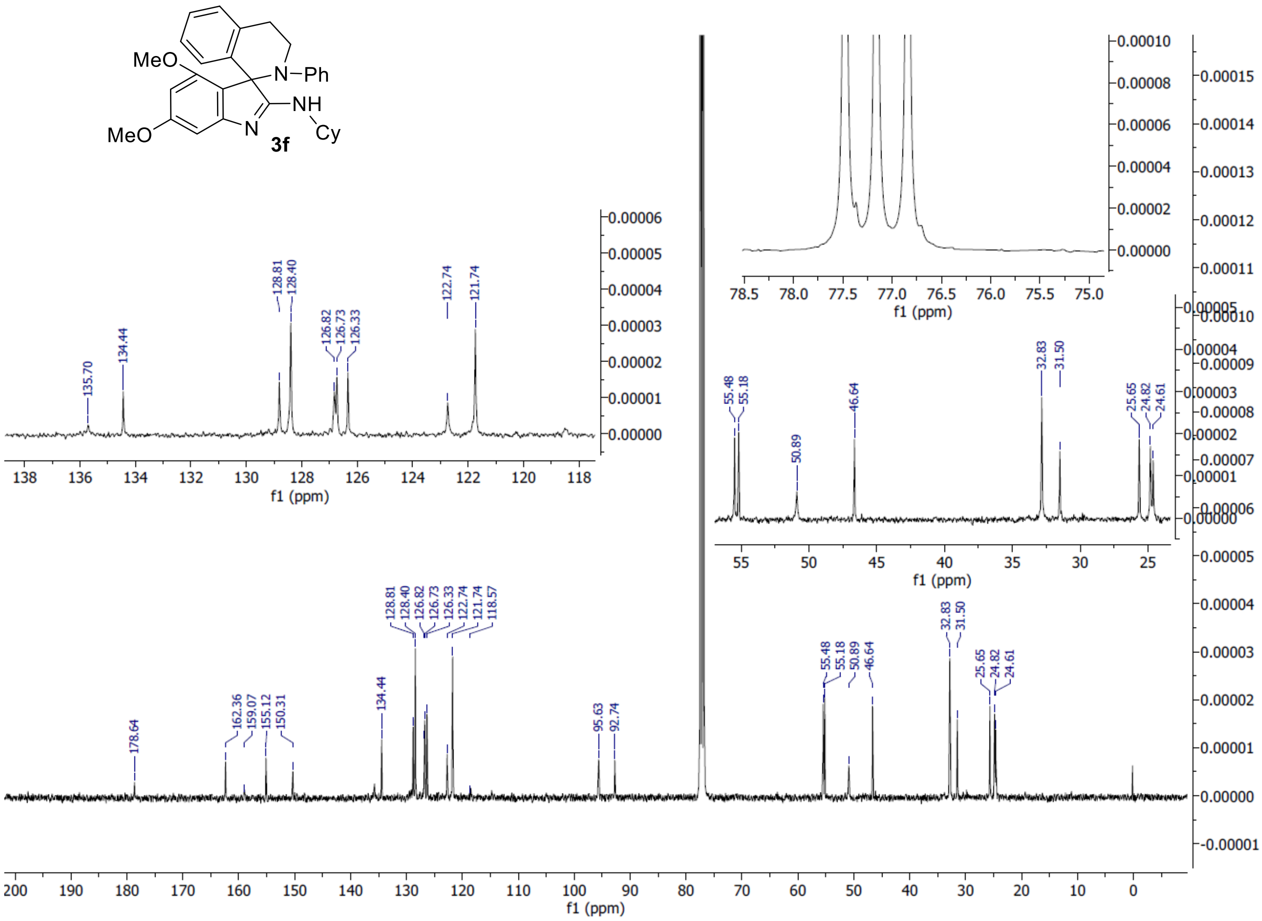
MeO
N−Ph
NH
MeO
N
3f
Cy
f1 (ppm)

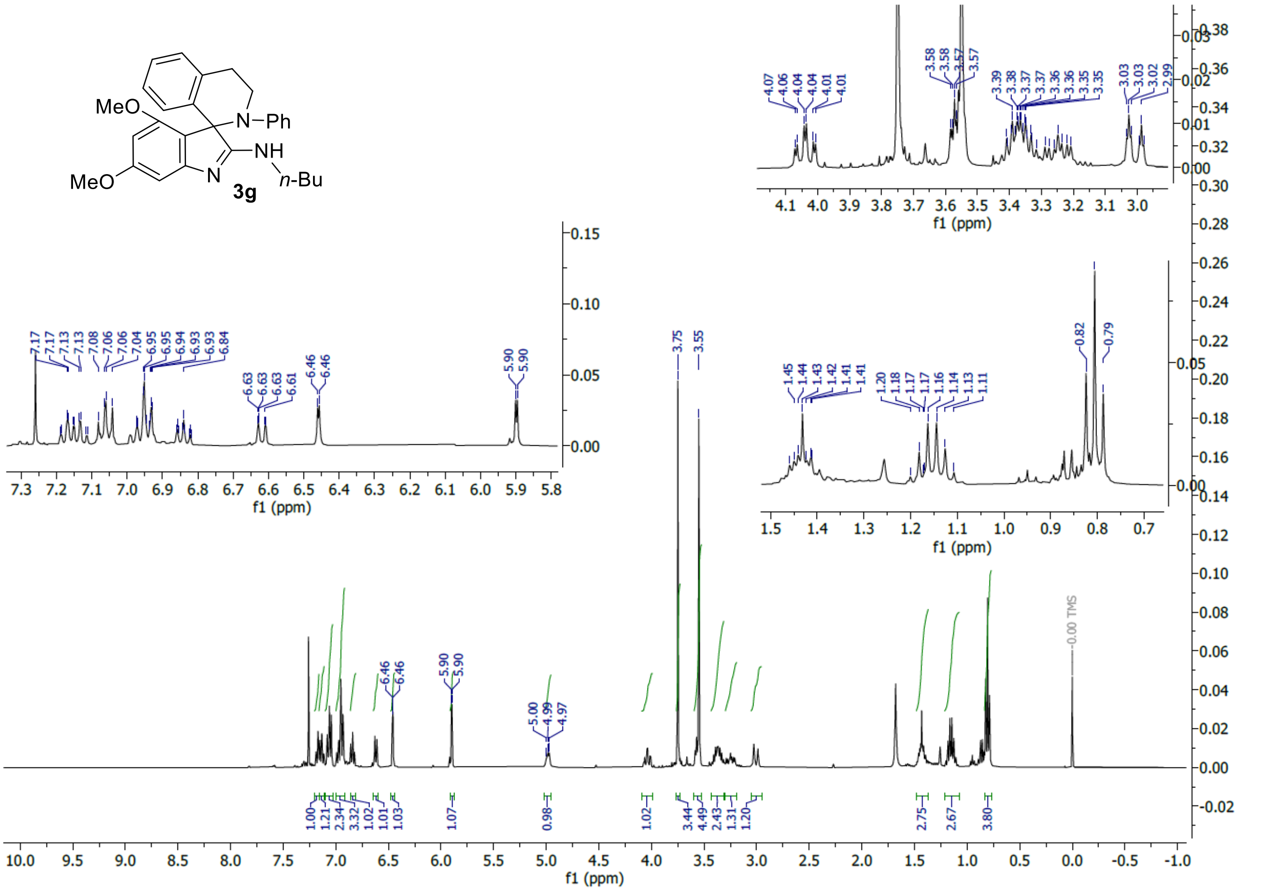
MeO
N−Ph
NH
MeO
N
3g
n-Bu
f1 (ppm)

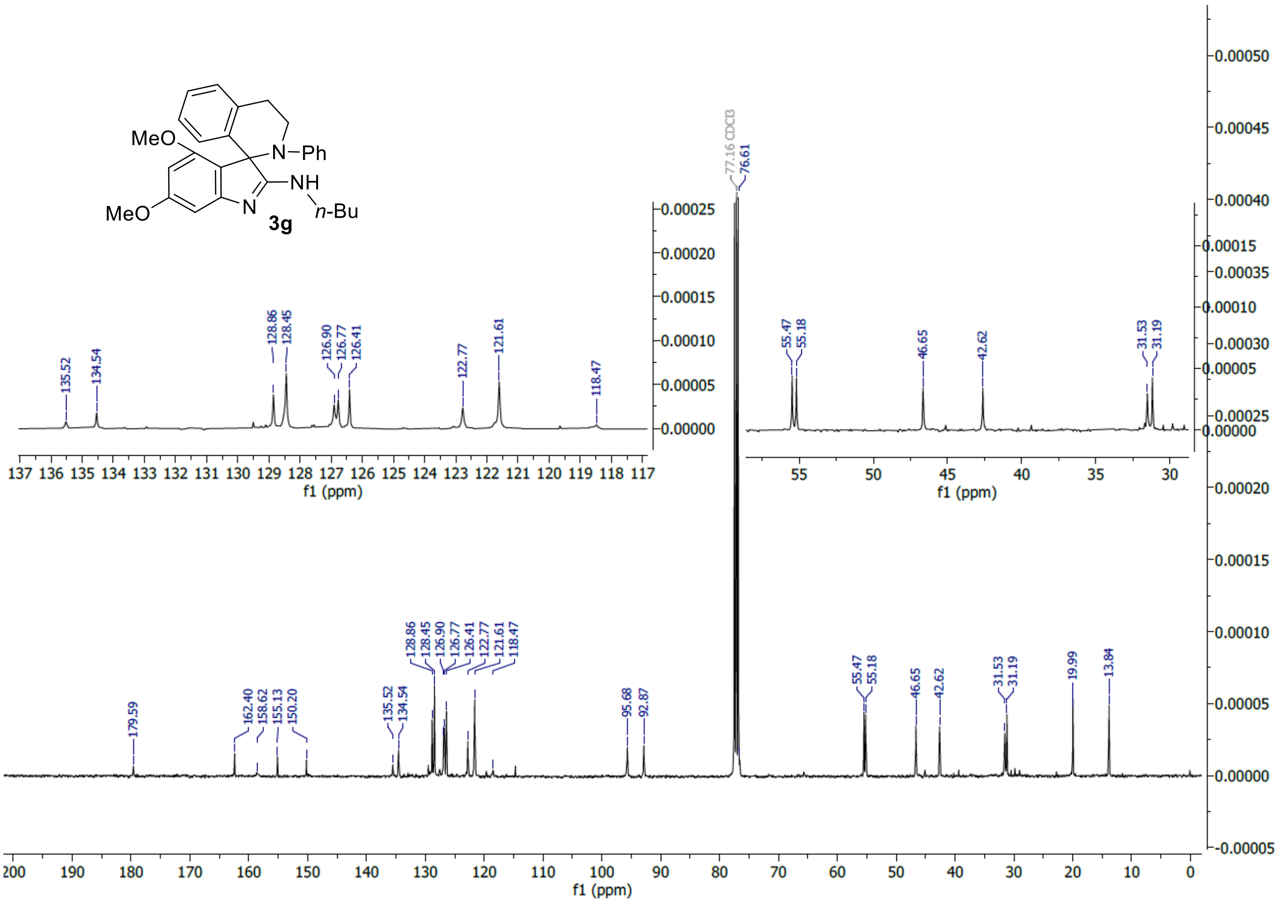
MeO
N–Ph
NH
MeO
N
3g
n-Bu
77.16 CDCl3
76.61
f1 (ppm)

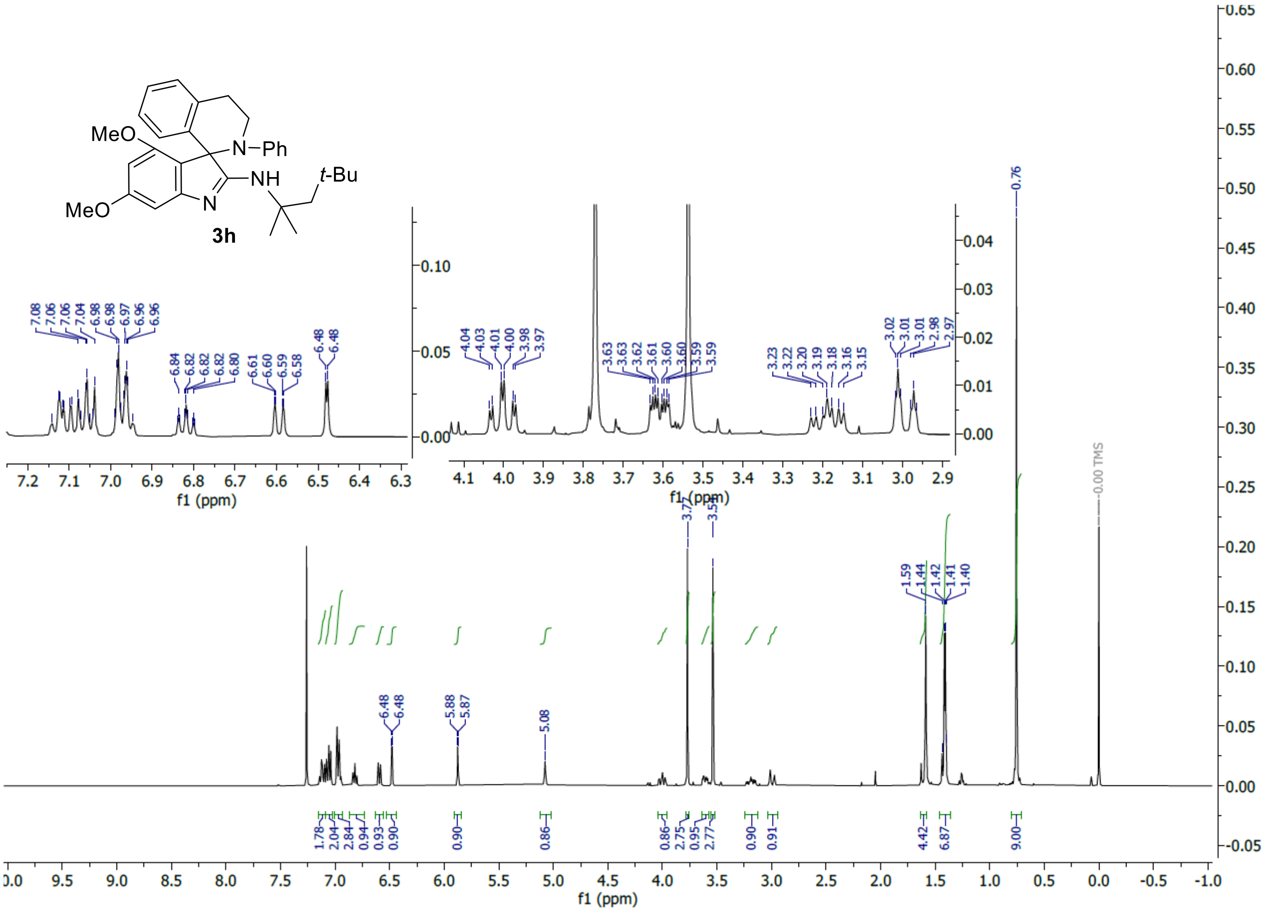
MeO
N–Ph
NH
t-Bu
MeO
N
3h
0.00 TMS
f1 (ppm)

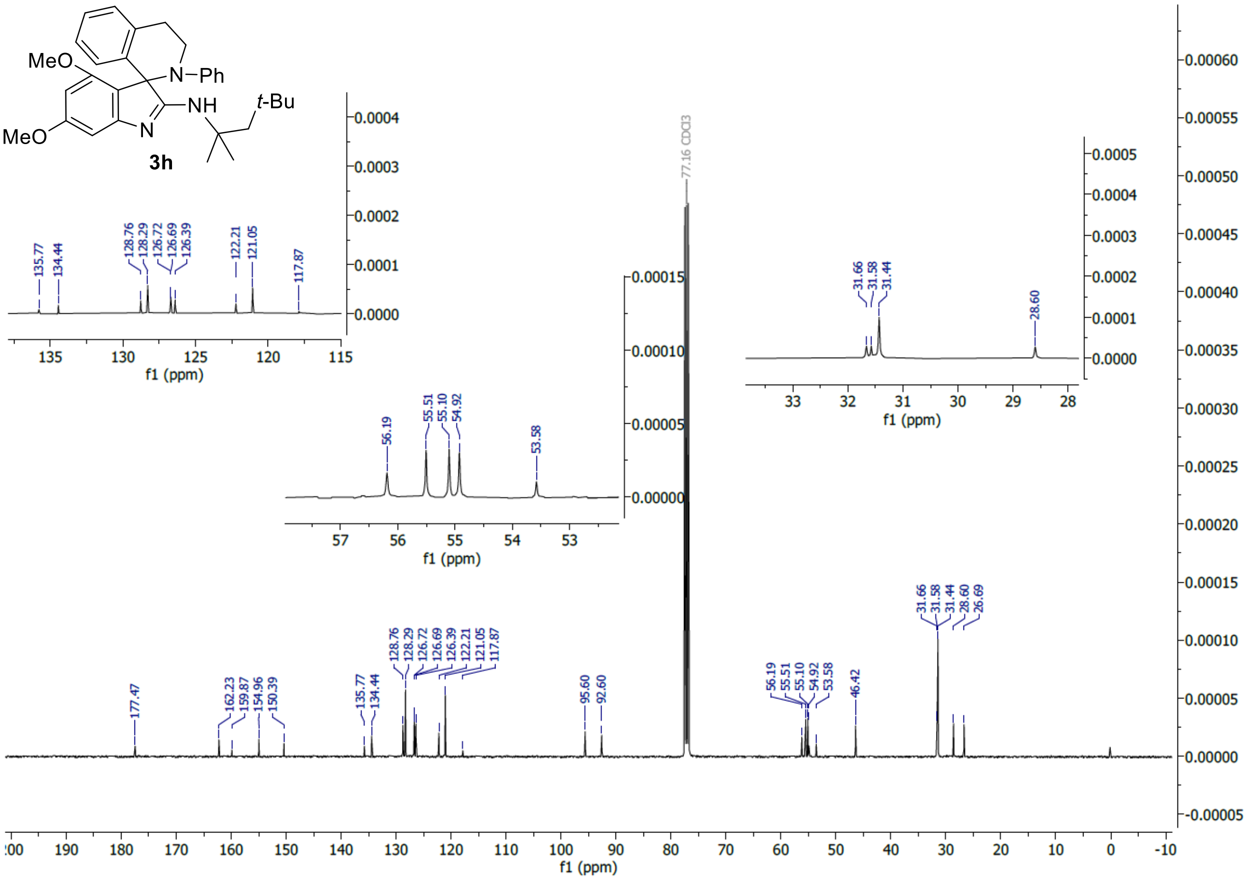
MeO
N–Ph
NH
t-Bu
MeO
N
3h
77.16 CDCl3
177.47
162.23 159.87 154.96 150.39
135.77 134.44
128.76 128.29 126.72 126.69 126.39 122.21 121.05 117.87
95.60 92.60
56.19 55.51 55.10 54.92 53.58
46.42
31.66 31.58 31.44 28.60 26.69
f1 (ppm)

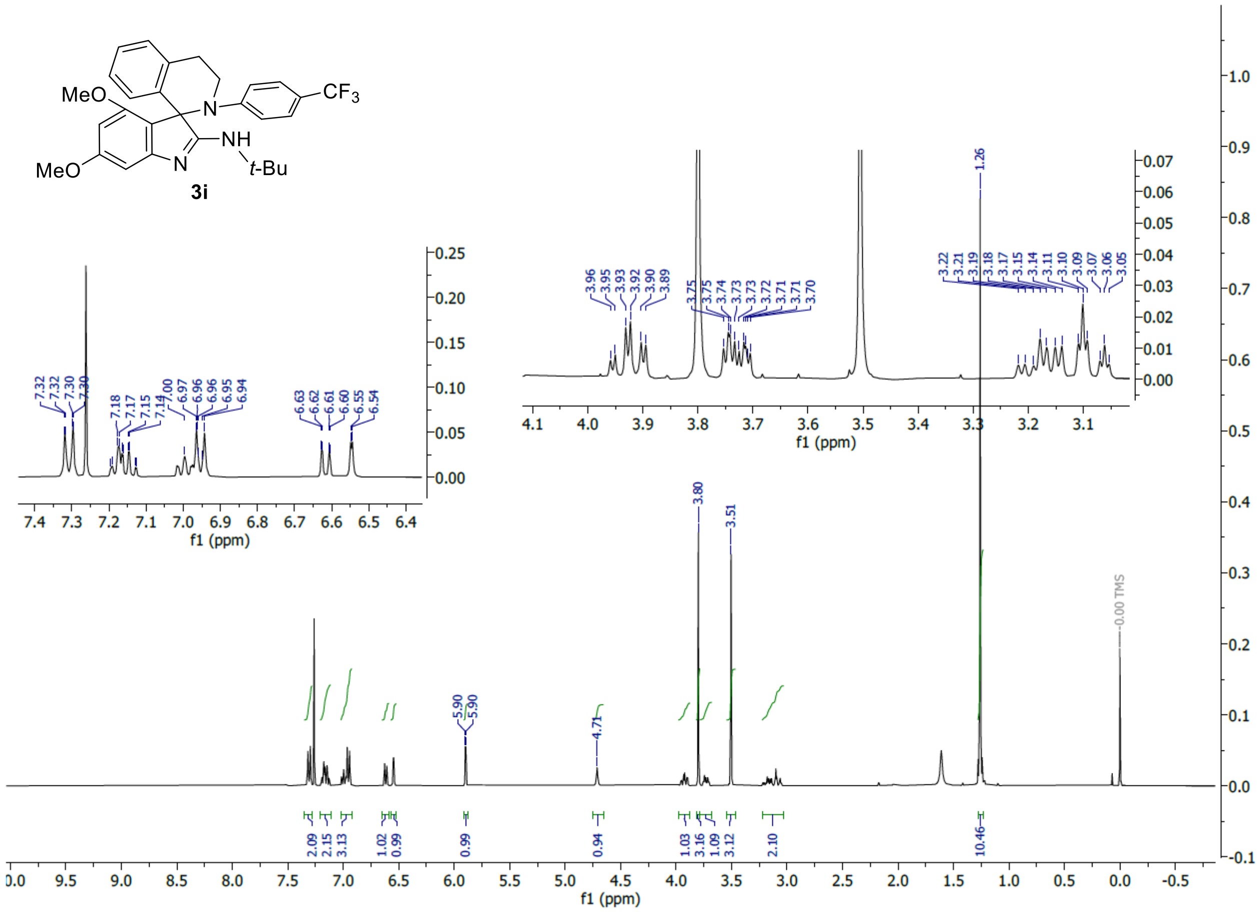
MeO
N
CF3
NH
t-Bu
MeO
N
3i
7.32 7.32 7.30 7.30 7.18 7.17 7.15 7.14 7.00 6.97 6.96 6.96 6.95 6.94 6.63 6.62 6.61 6.60 6.55 6.54
5.90 5.90
4.71
3.96 3.95 3.93 3.92 3.90 3.89 3.75 3.75 3.74 3.73 3.73 3.72 3.71 3.71 3.70
3.80
3.51
3.22 3.21 3.19 3.18 3.17 3.15 3.14 3.11 3.10 3.09 3.07 3.06 3.05
1.26
0.00 TMS
f1 (ppm)

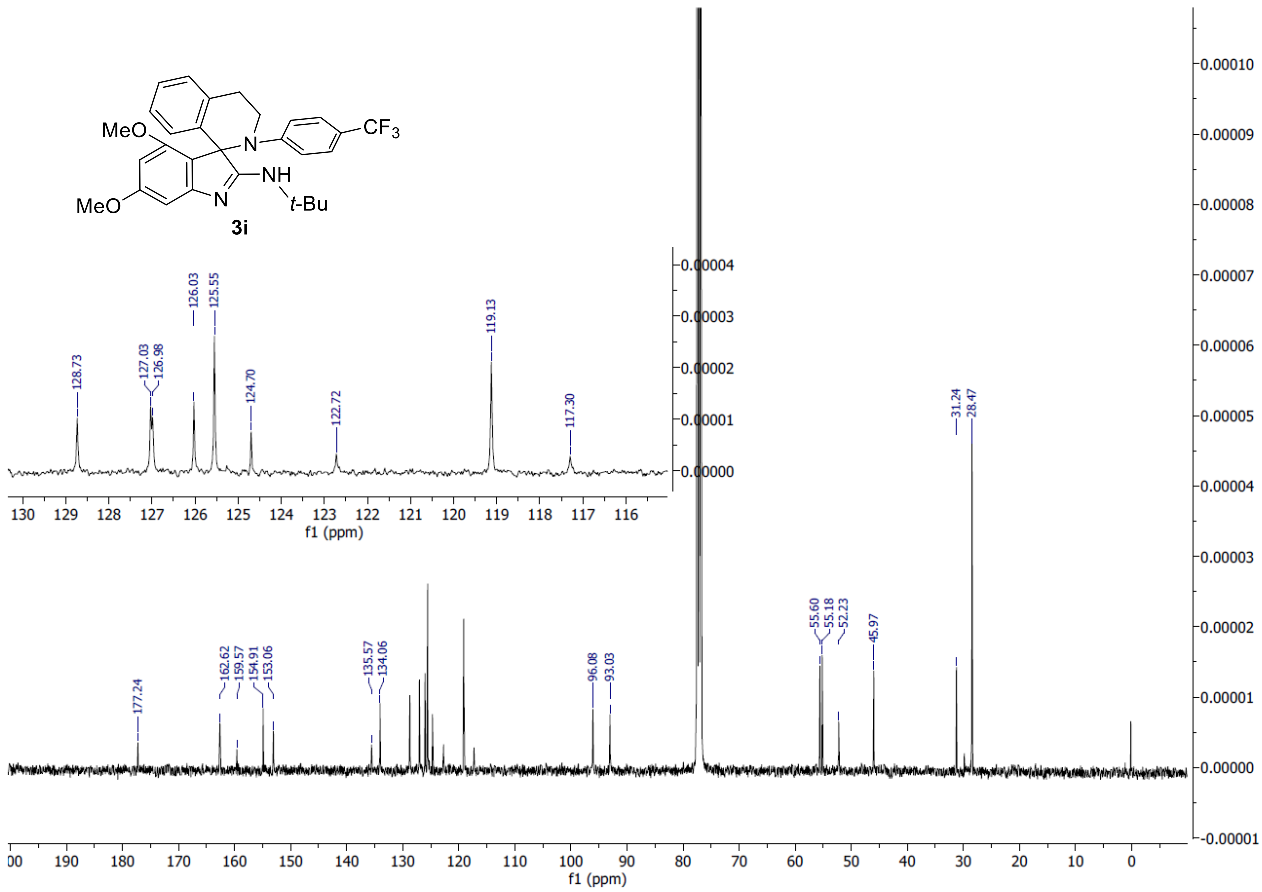
MeO
MeO
N
NH
t-Bu
CF3
3i
128.73
127.03
126.98
126.03
125.55
124.70
122.72
119.13
117.30
177.24
162.62
159.57
154.91
153.06
135.57
134.06
96.08
93.03
55.60
55.18
52.23
45.97
31.24
28.47
f1 (ppm)

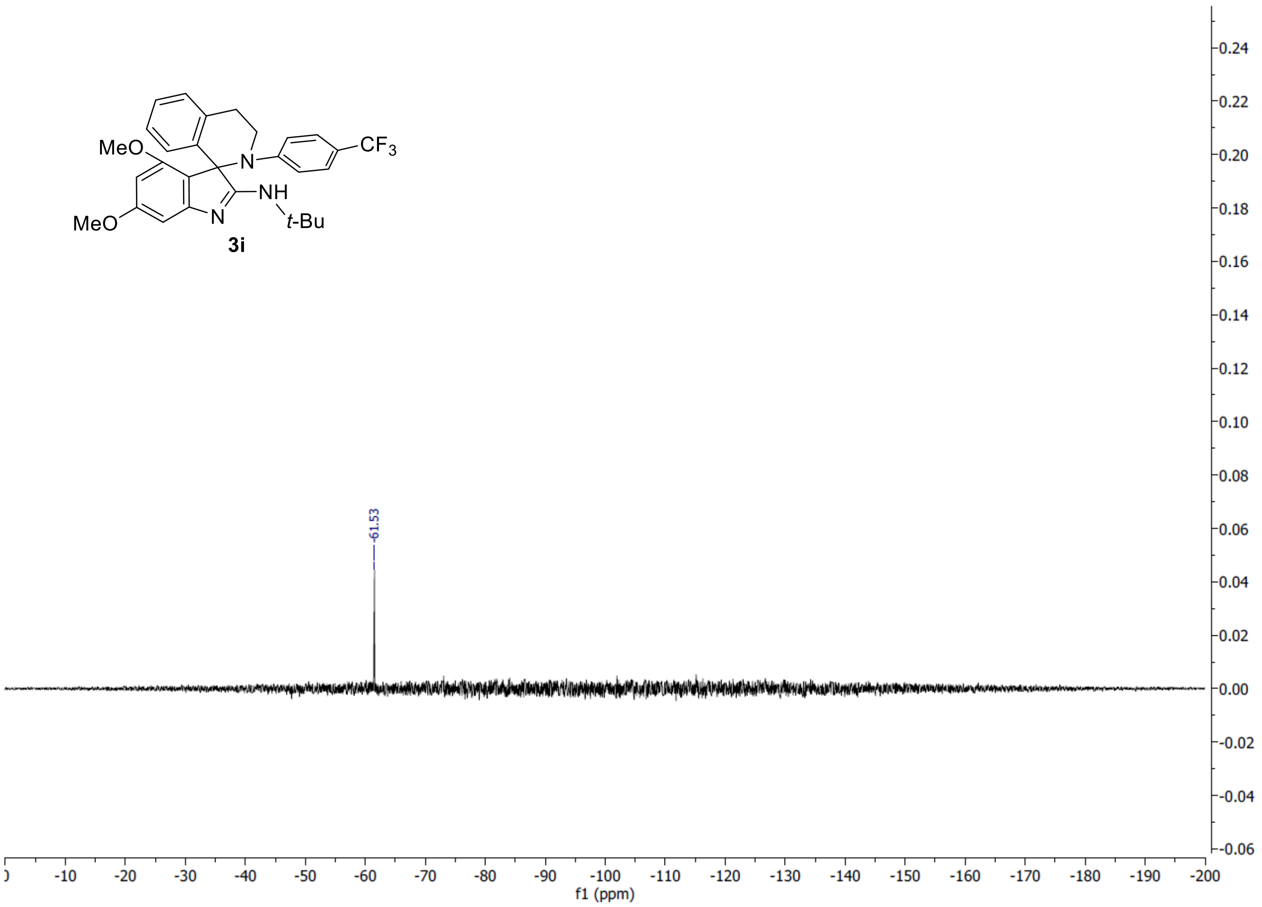
MeO
MeO
N
NH
t-Bu
CF3
3i
-61.53
f1 (ppm)

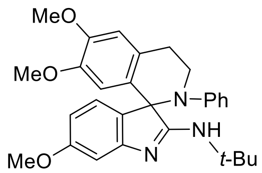
MeO
MeO
N−Ph
NH
t-Bu
MeO
N

3j

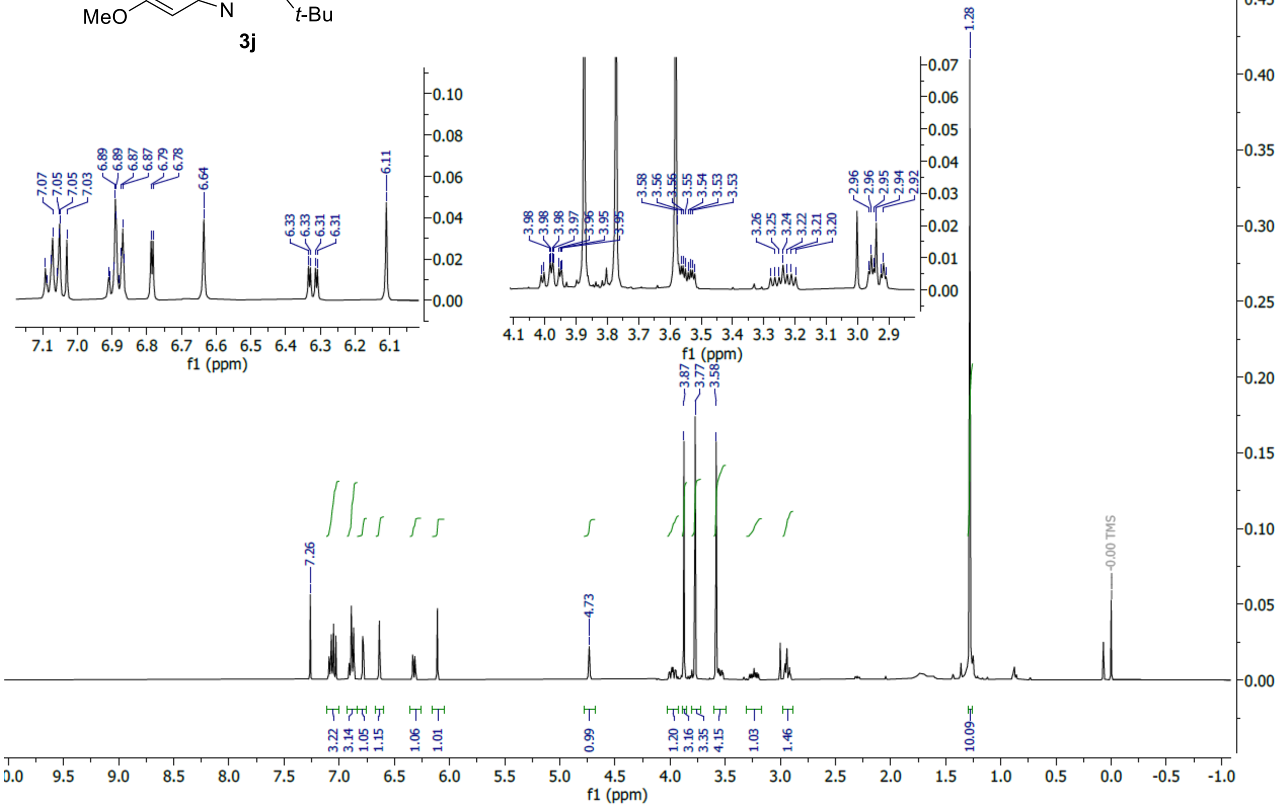
7.07
7.05
7.05
7.03
6.89
6.89
6.87
6.87
6.79
6.78
6.64
6.33
6.33
6.31
6.31
6.11
3.98
3.98
3.98
3.97
3.96
3.95
3.95
3.58
3.56
3.56
3.55
3.54
3.53
3.53
3.26
3.25
3.24
3.22
3.21
3.20
2.96
2.96
2.95
2.94
2.92
1.28
3.87
3.77
3.58
7.26
4.73
0.00 TMS
3.22
3.14
1.05
1.15
1.06
1.01
0.99
1.20
3.16
3.35
4.15
1.03
1.46
10.09
f1 (ppm)

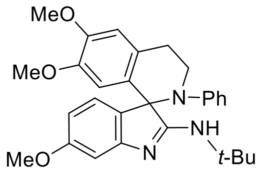
MeO
MeO
N−Ph
NH
t-Bu
MeO
N

3j

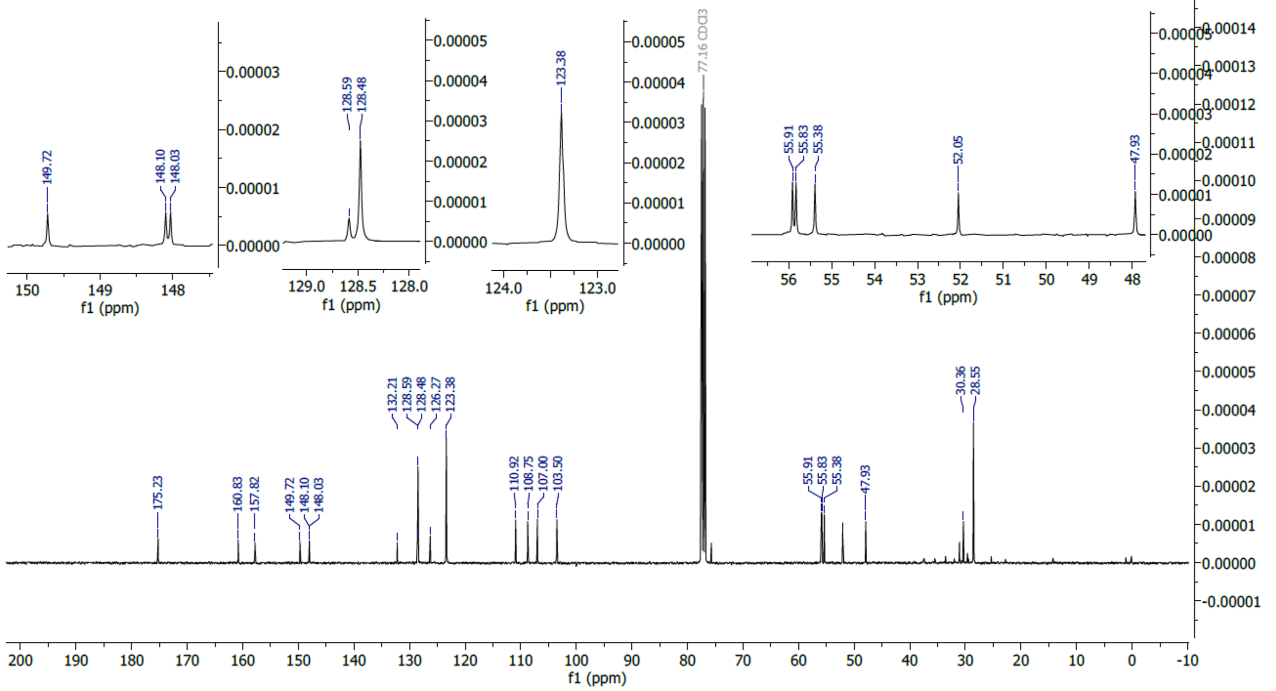
149.72
148.10
148.03
128.59
128.48
123.38
77.16 CDCl3
55.91
55.83
55.38
52.05
47.93
175.23
160.83
157.82
132.21
126.27
110.92
108.75
107.00
103.50
30.36
28.55
f1 (ppm)

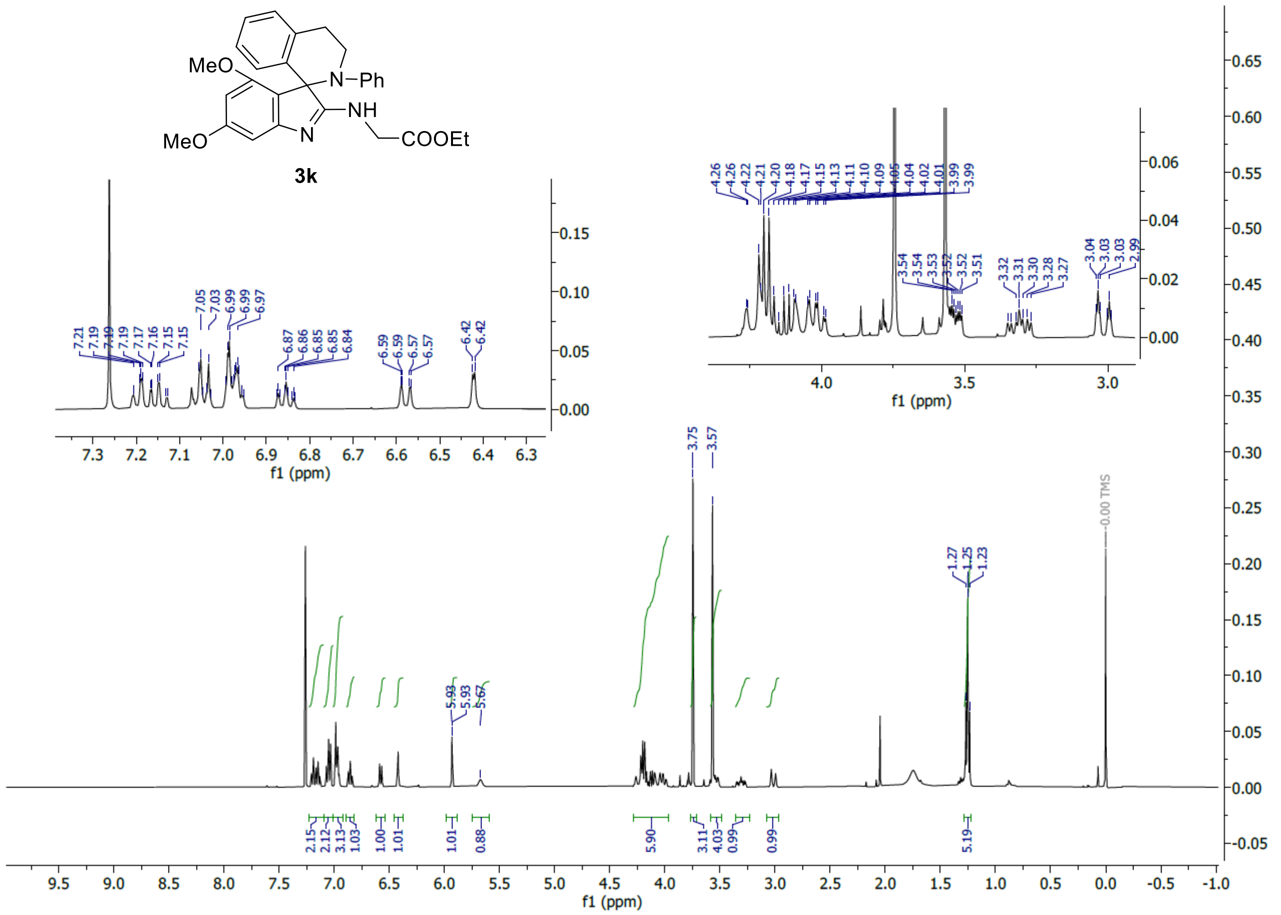

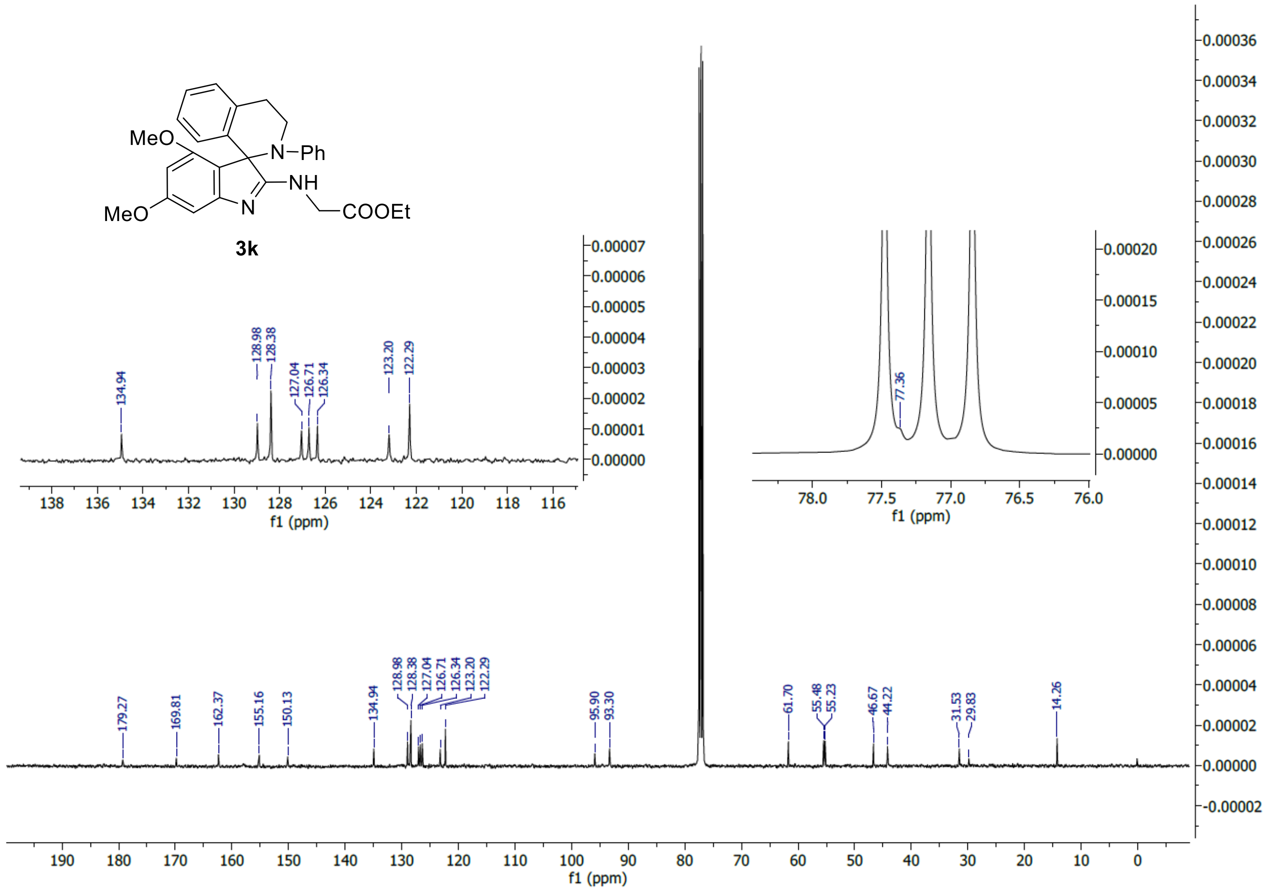

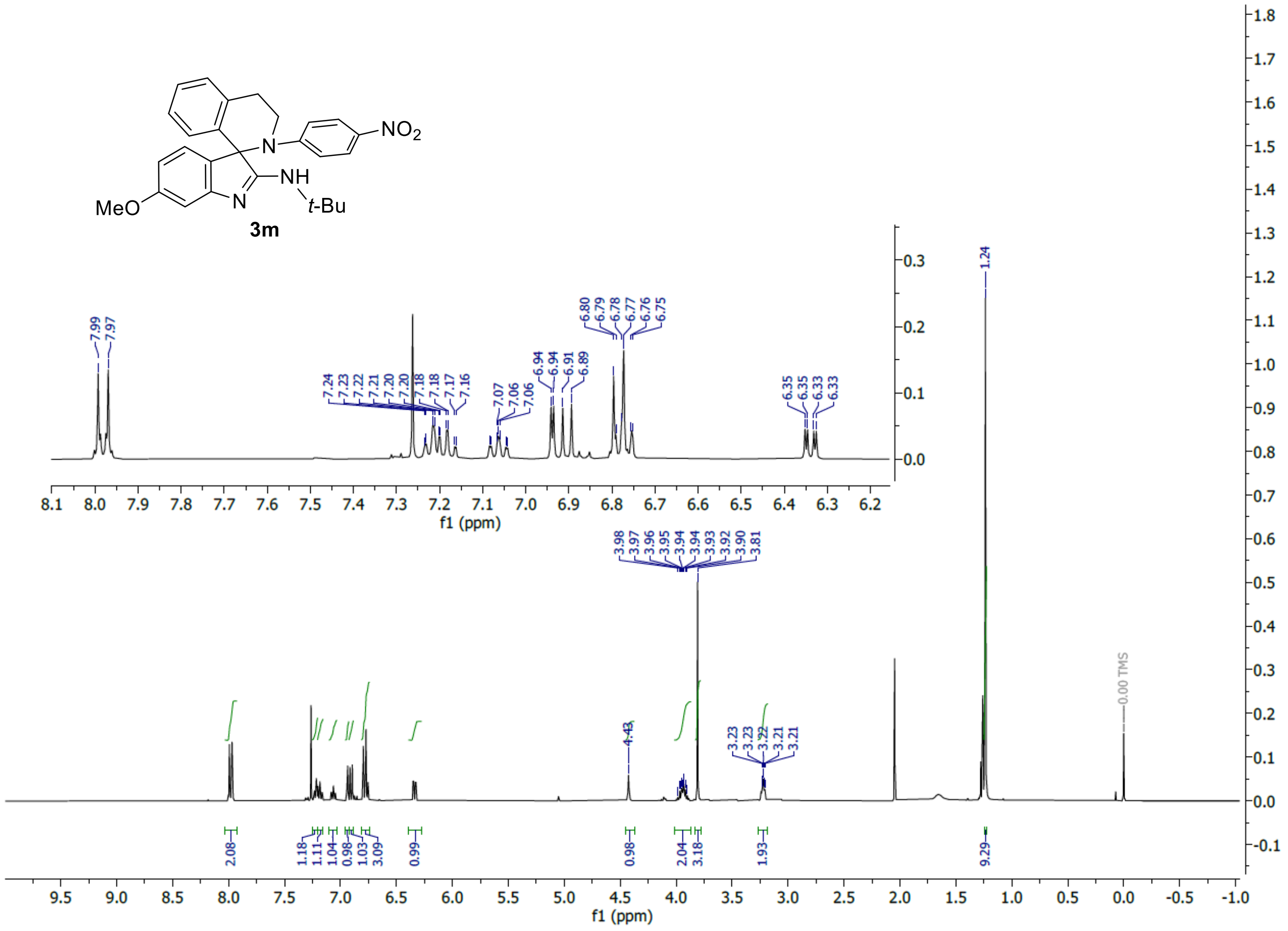
NO2
NH
MeO
N
t-Bu
3m
f1 (ppm)

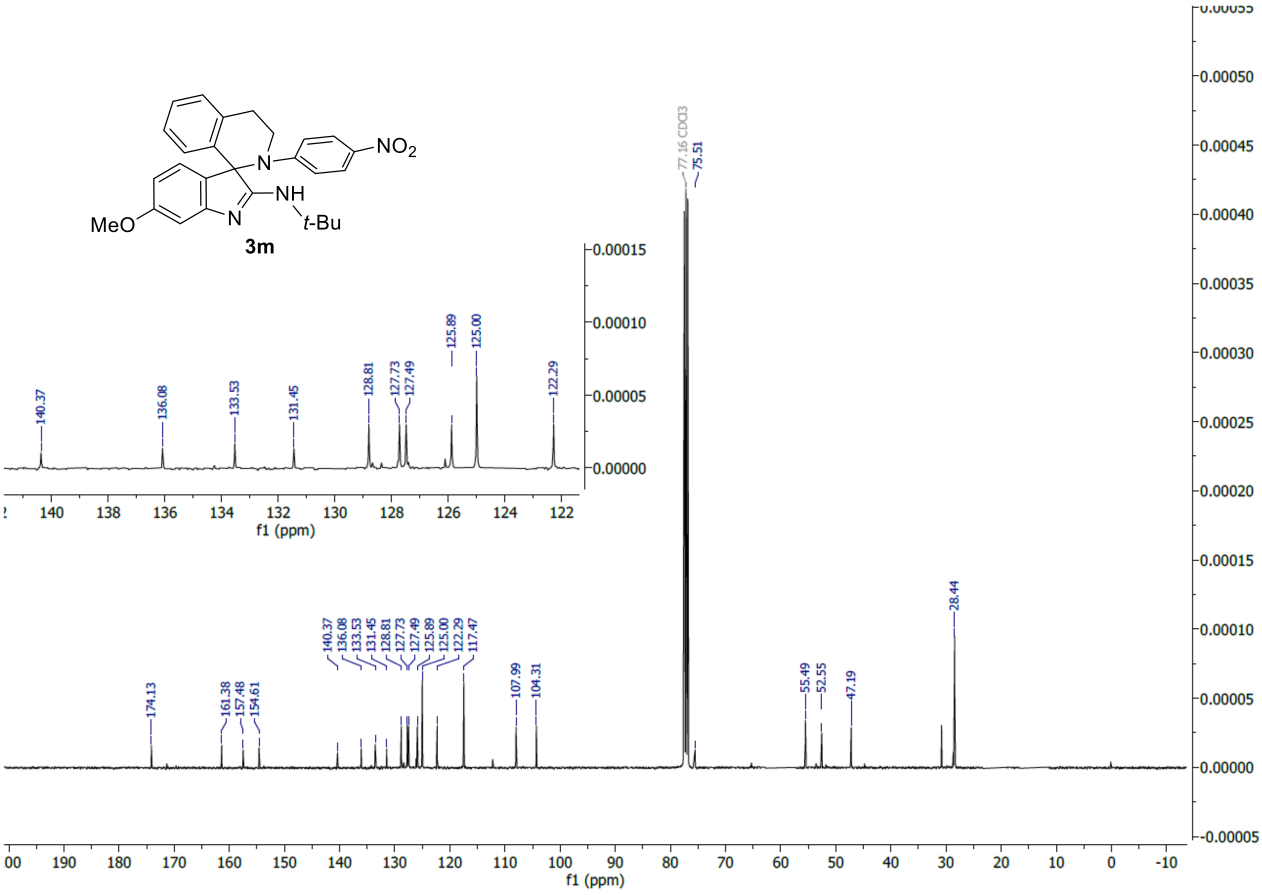
NO2
NH
MeO
N
t-Bu
3m
77.16 CDCl3
f1 (ppm)

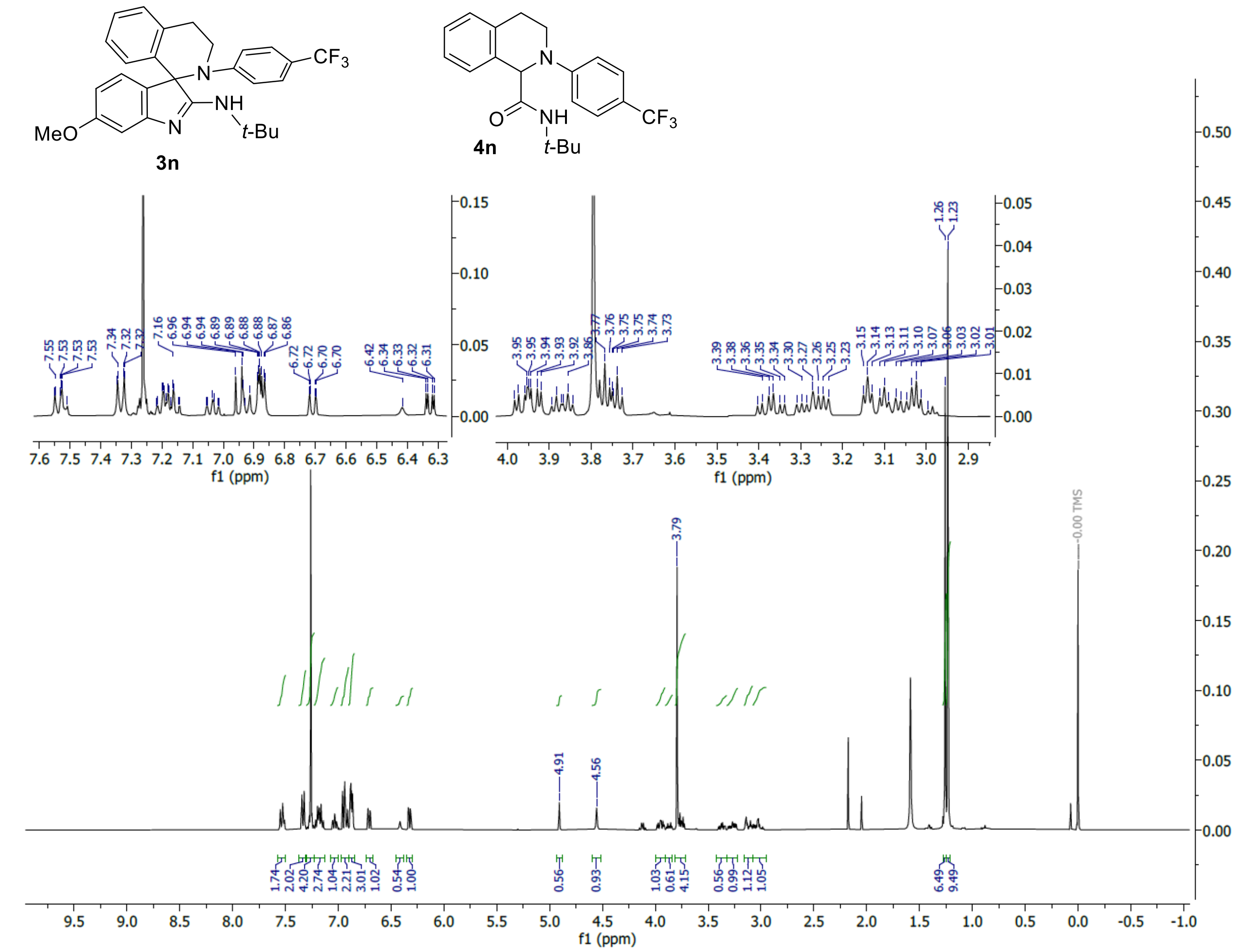
3n
4n
MeO
CF3
NH
t-Bu
N
O
f1 (ppm)
0.00 TMS

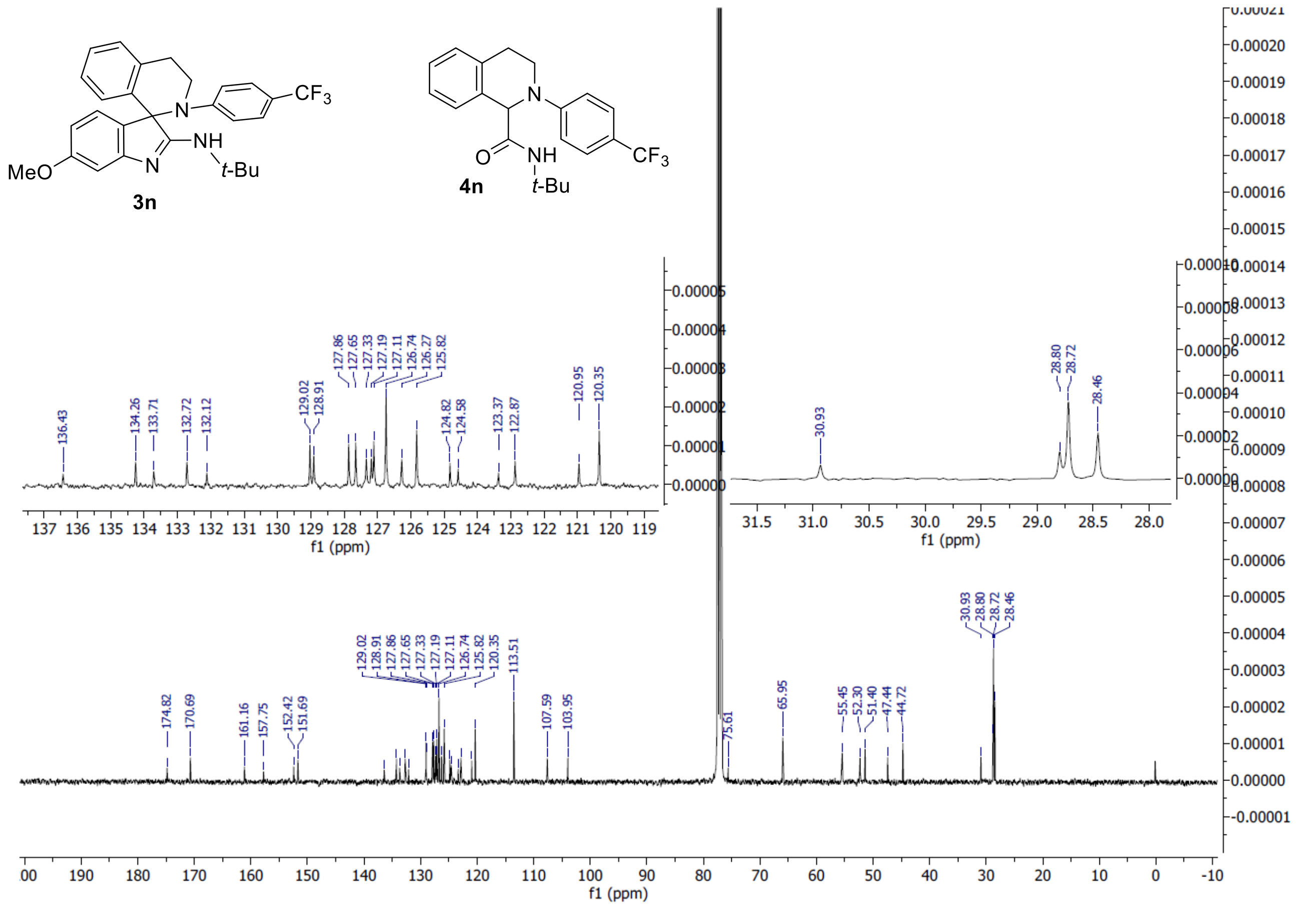
3n
4n
MeO
CF3
NH
t-Bu
N
O
f1 (ppm)

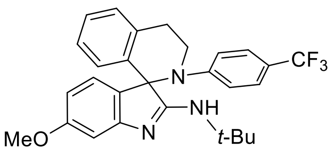

3n

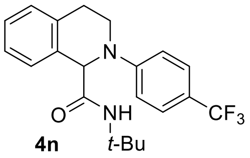

4n

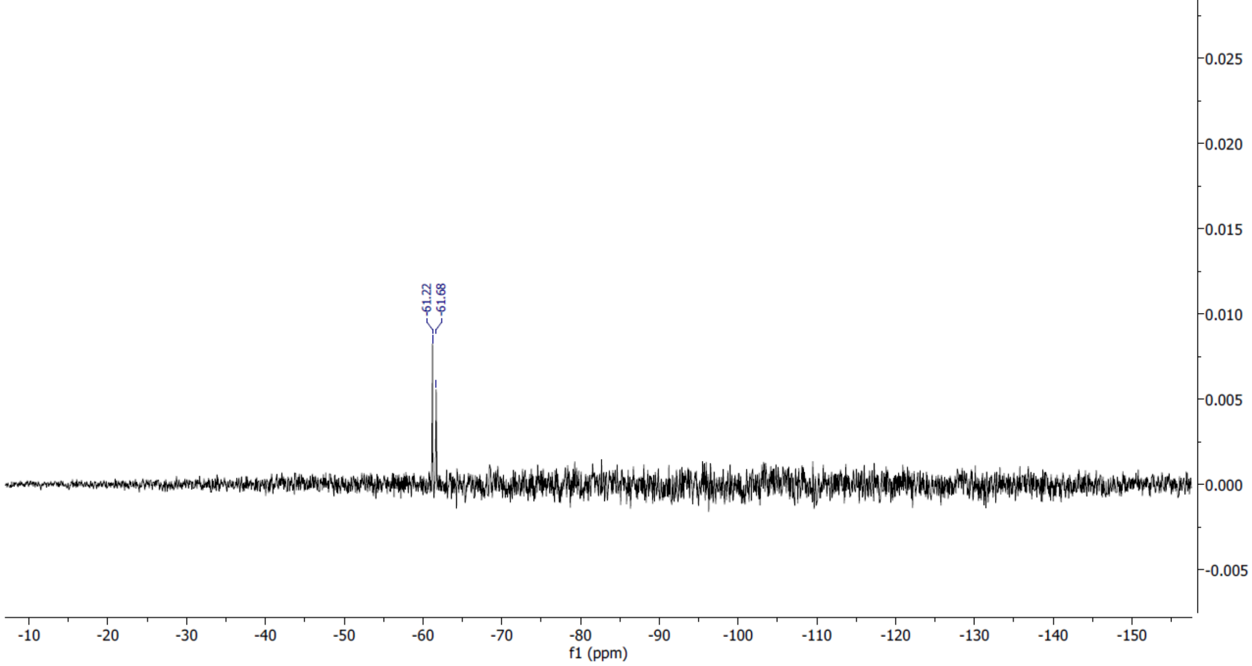

-61.22
-61.68
0.040
0.035
0.030
0.025
0.020
0.015
0.010
0.005
0.000
-0.005
-10
-20
-30
-40
-50
-60
-70
-80
-90
-100
-110
-120
-130
-140
-150
f1 (ppm)

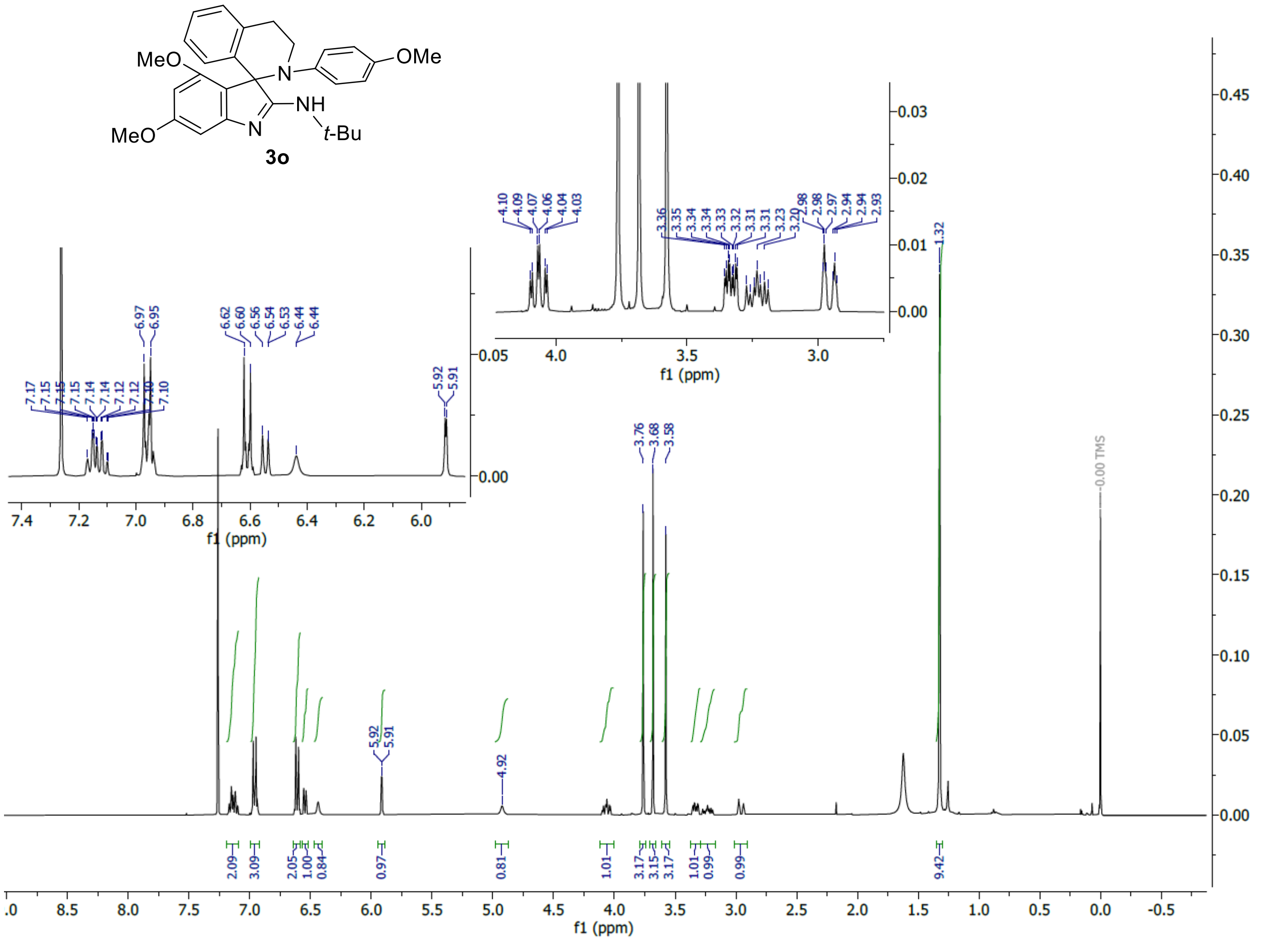

3o

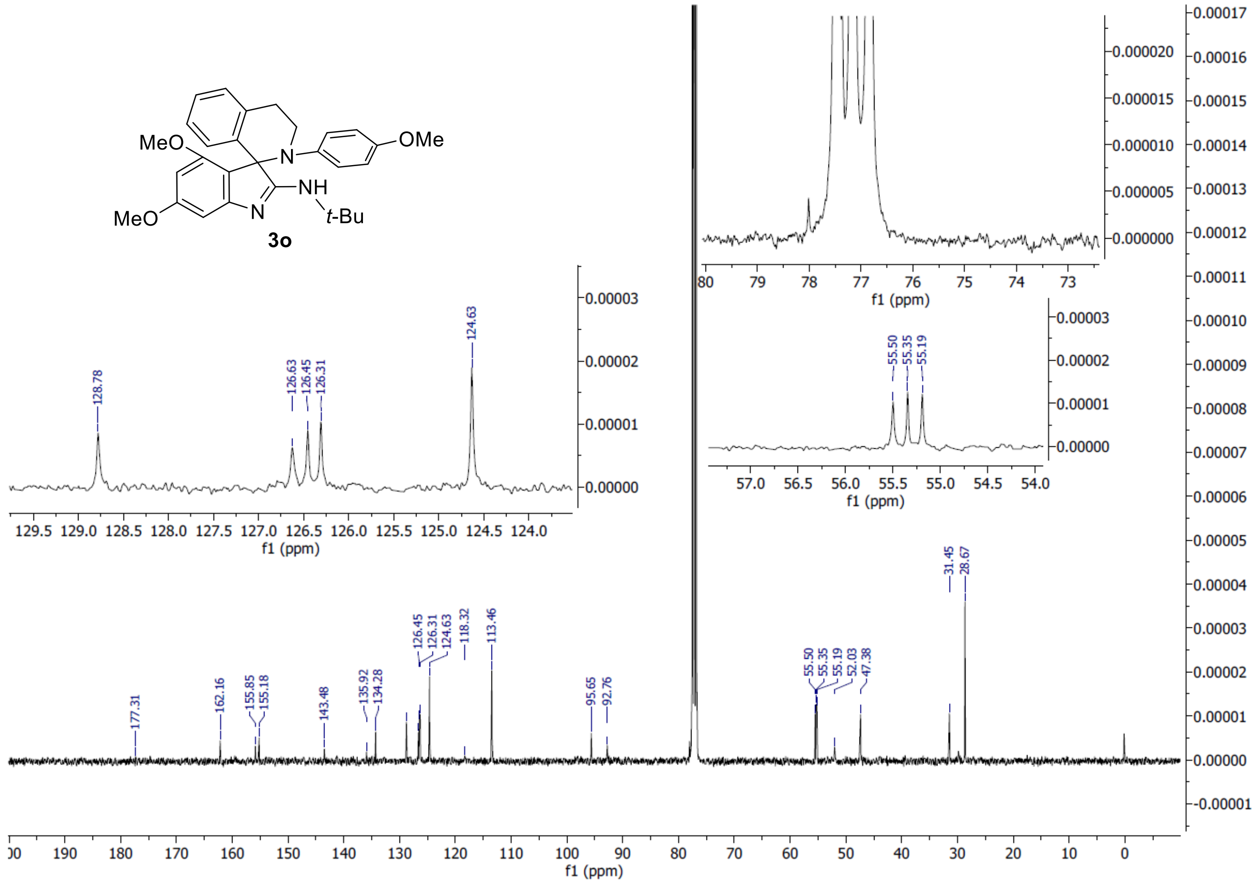

3o

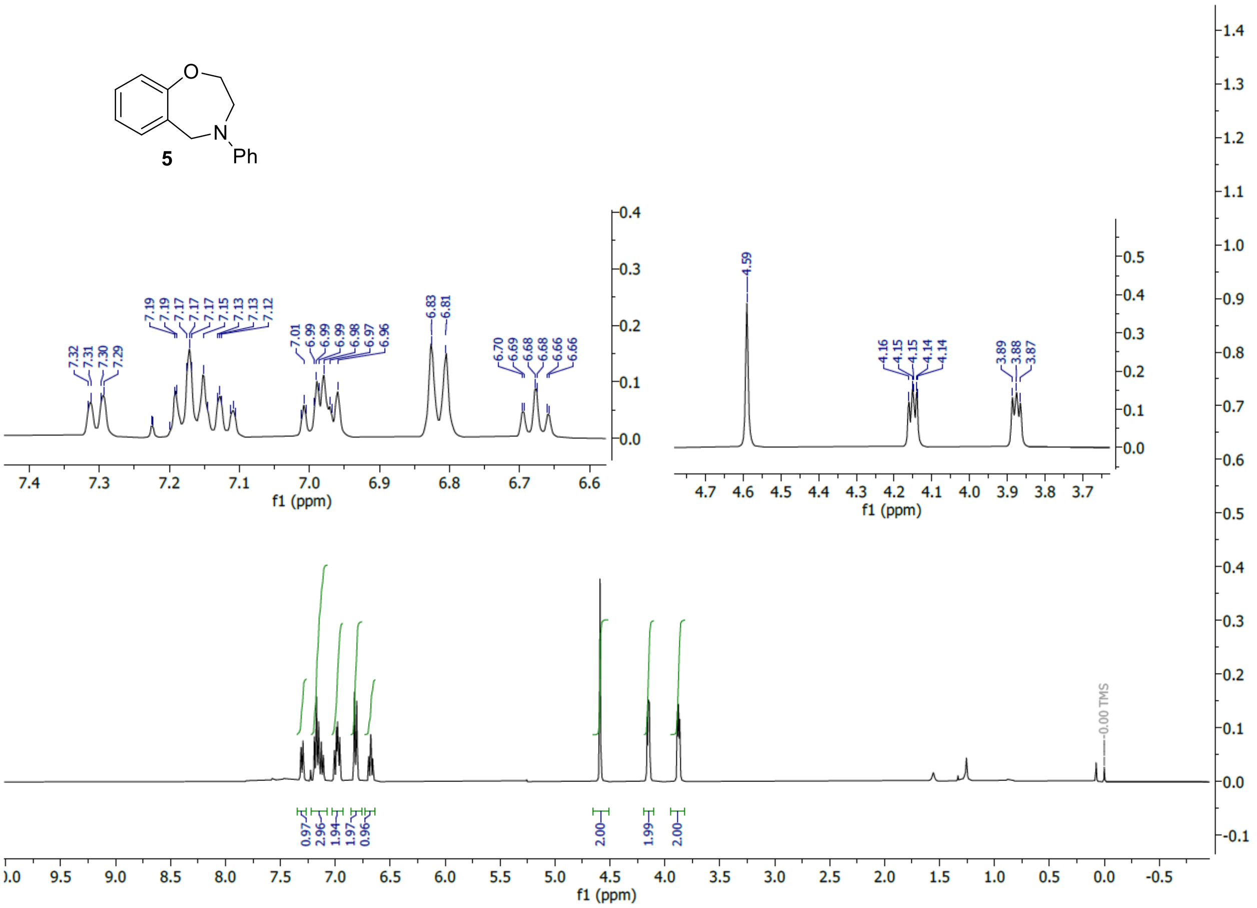

5
Ph
7.32 7.31 7.30 7.29
7.19 7.19 7.17 7.17 7.17 7.15 7.13 7.13 7.12
7.01 6.99 6.99 6.99 6.98 6.97 6.96
6.83 6.81
6.70 6.69 6.68 6.68 6.66 6.66
4.59
4.16 4.15 4.15 4.14 4.14
3.89 3.88 3.87
0.00 TMS
0.97 2.96 1.94 1.97 0.96
2.00 1.99 2.00
f1 (ppm)

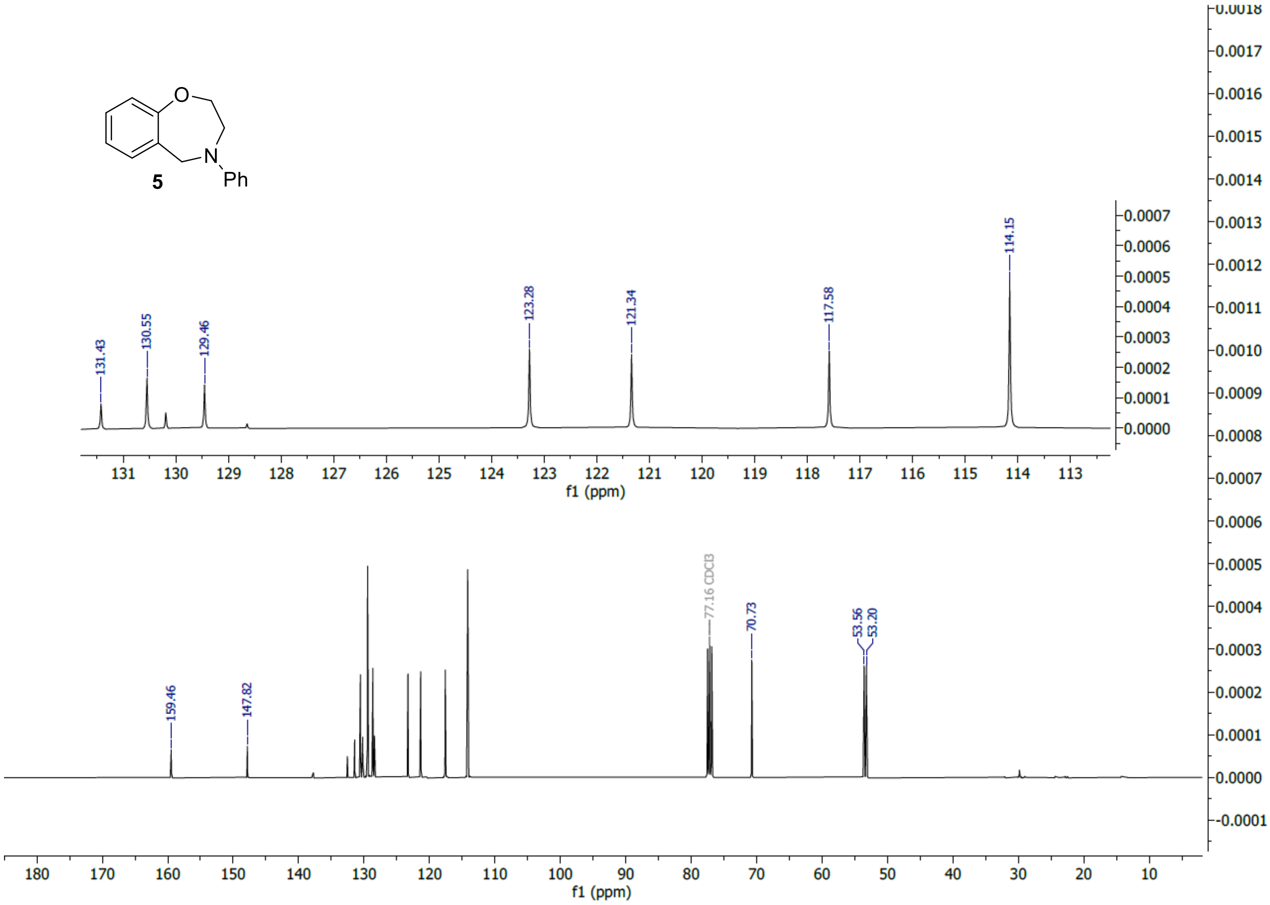

5
Ph
131.43 130.55 129.46
123.28
121.34
117.58
114.15
159.46
147.82
77.16 CDCl3
70.73
53.56 53.20
f1 (ppm)

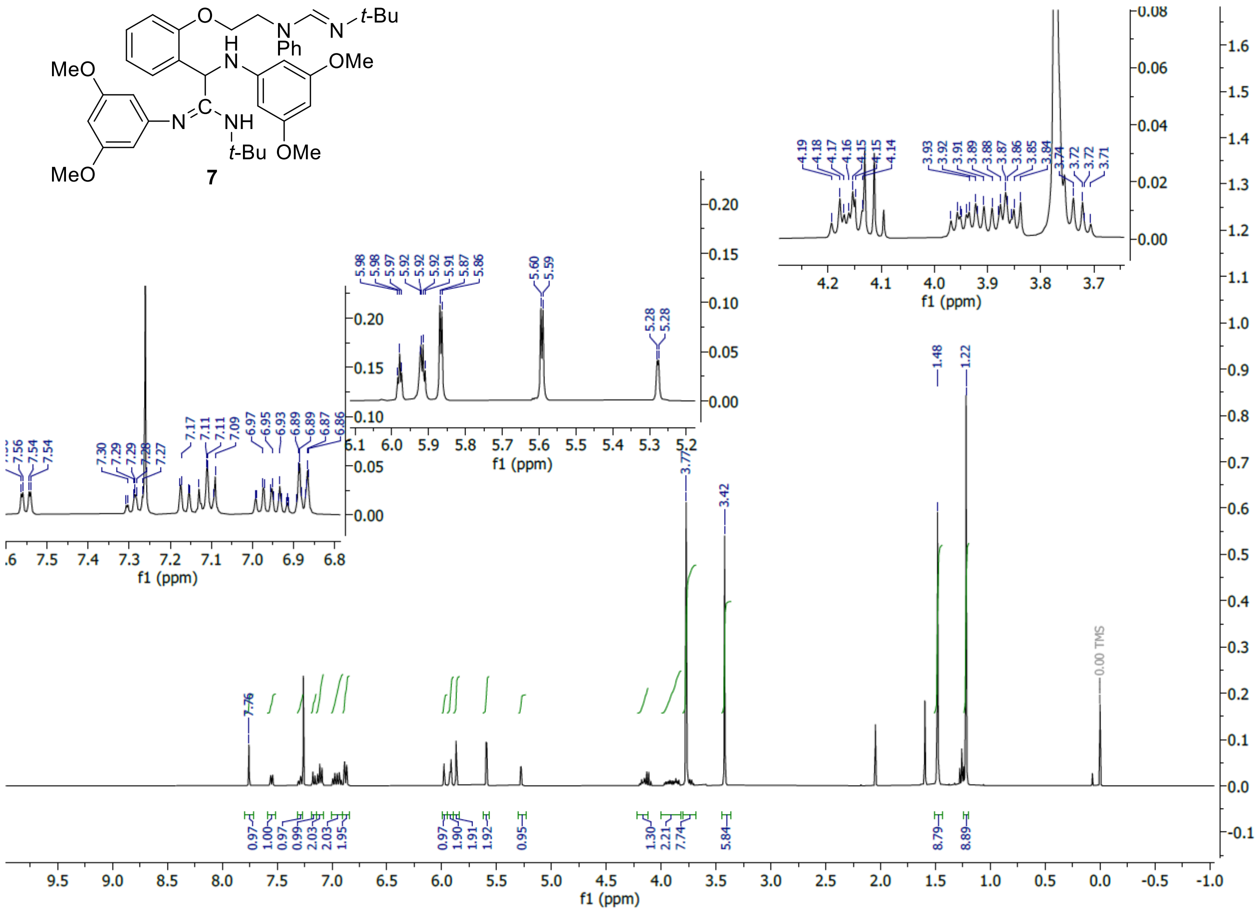

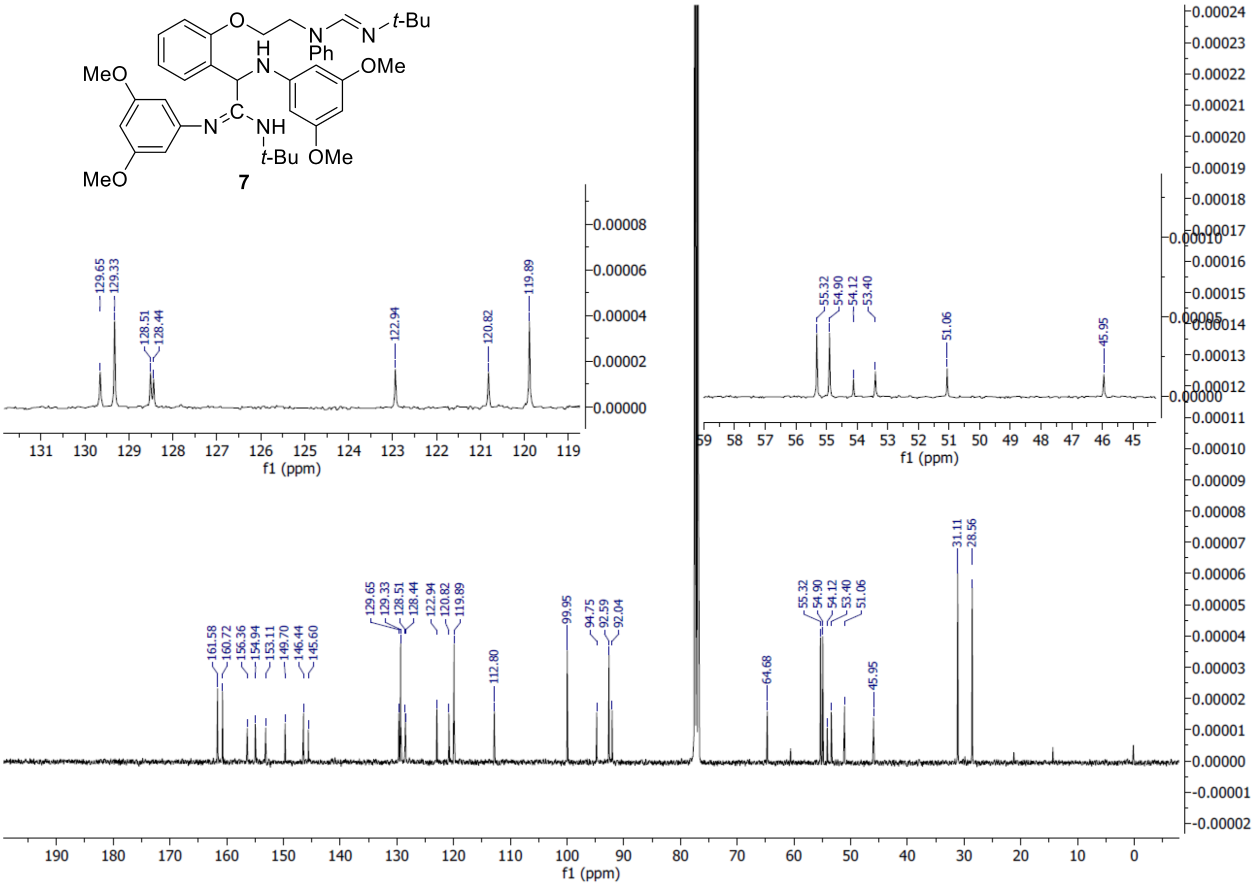

Supplement: File 1 — Additional optimization studies; description of photochemical equipment; characterization data of compounds (1H, 13C and 19F NMR spectra, IR and UV–vis spectra). [file Beilstein_J_Org_Chem-20-2722-s001.pdf]
